# Association Between Consumption of Fermented Food and Food-Derived Prebiotics With Cognitive Performance, Depressive, and Anxiety Symptoms in Psychiatrically Healthy Medical Students Under Psychological Stress: A Prospective Cohort Study

**DOI:** 10.3389/fnut.2022.850249

**Published:** 2022-03-03

**Authors:** Michał Seweryn Karbownik, Łukasz Mokros, Maria Dobielska, Mateusz Kowalczyk, Edward Kowalczyk

**Affiliations:** ^1^Department of Pharmacology and Toxicology, Medical University of Lodz, Łódź, Poland; ^2^Department of Clinical Pharmacology, Medical University of Lodz, Łódź, Poland; ^3^Students' Research Club, Department of Pharmacology and Toxicology, Medical University of Lodz, Łódź, Poland; ^4^Babinski Memorial Hospital, Łódź, Poland

**Keywords:** fermented food, probiotic, prebiotics, cognitive function, depression, anxiety, psychological stress

## Abstract

**Background:**

Gut microbiota-based therapeutic strategies, such as probiotic and prebiotic preparations, may benefit mental health. However, commonly consumed fermented and prebiotic-containing foods have not been well-tested. The aim of the present study was to determine whether consumption of fermented food and food-derived prebiotics is associated with cognitive performance, depressive, and anxiety symptoms in psychiatrically healthy medical students under psychological stress.

**Methods:**

The study protocol with data analysis plan was prospectively registered. Food consumption was evaluated with a 7-day dietary record. Cognitive performance was modeled with academic examination performance in relation to subject knowledge. Pre-exam depressive and anxiety symptoms were assessed with the Patient Health Questionnaire-9 and Generalized Anxiety Disorder-7, respectively.

**Results:**

In total, 372 medical students (22.7 ± 1.1 years of age, 66% female) completed the study. No relationship was observed between cognitive performance under stress and either fermented food (adjusted β 0.02, 95% CI −0.07–0.11, *p* = 0.63) or food-derived prebiotics consumption (adjusted β −0.00, 95% CI −0.09–0.09, *p* = 0.99). High intake of fermented food was associated with more severe depressive (adjusted β 0.11, 95% CI 0.01–0.20, *p* = 0.032) and anxiety symptoms under stress (adjusted β 0.13, 95% CI 0.04–0.22, *p* = 0.0065); however, no such link was observed for food-derived prebiotics (adjusted β 0.03, 95% CI −0.07–0.13, *p* = 0.50 and −0.01, 95% CI −0.11–0.08, *p* = 0.83, for depression and anxiety, respectively).

**Conclusions:**

Under psychological stress in medical students, consumption of fermented food and food-derived prebiotics appears to be not associated with cognitive performance. High intake of fermented food, but not food-derived prebiotics, may be associated with severity of depressive and anxiety symptoms. The safety of fermented food in this regard therefore requires further clarification.

## Introduction

Cognitive functioning is a major keystone of humanity. A specific constellation of our cognitive abilities (including attention, memory, problem solving, reasoning, or planning, etc.) enabled functioning in social groups, adopting to ever-changing environment and have driven our civilization to unprecedented development (1). However, human cognition is often endangered in multiple domains: seniors may suffer from dementia, and neuropsychiatric patients manifest some degree of cognitive decline, which frequently accompanies depression, bipolar and anxiety disorders, schizophrenia and epilepsy. Even children may be affected by cognitive decline associated with attention deficit and autism spectrum disorders (2). In fact, virtually all pathologies appear to be linked to cognitive dysfunctions to some extent (3–5), including coronavirus disease 2019 (COVID-19) (6).

Cognitive abilities may also deteriorate in healthy people experiencing psychological stress and anxiety (7–9), which are common in everyday life (10). Notably, a threat response to a stressor is capable of impairing memory retrieval, as highlighted in a cold pressor test in healthy young adults (11), and attention being redirected toward the stressor (12, 13). This decrease in cognitive functioning may be harmful for people operating in challenging conditions such as emergency department staff or students taking an examination. The latter group, may suffer diminished academic achievements and frustrated future success in professional career (14, 15). There is a clear need to identify and implement strategies to improve cognitive outcomes and reduce anxiety.

One promising strategy for restoration and maintenance of cognitive functioning is the modulation of gut microbiota (16–19). This proposal has been supported by a range of evidence including animal interventional studies (20–28) along with human correlational (29–36) and interventional research involving probiotic (37–40) and prebiotic preparations (41) as well as well-defined culture-dependent fermented foods (42–47). For example, memory dysfunction was observed in germ-free or enteric pathogen-infected previously healthy mice exposed to acute stress; importantly, the memory decline was prevented by the use of a commercially-available probiotic combination (22). Elsewhere, motor speed and attention, and the organization of the brain microstructure in the amygdala and thalamus, as revealed by fractional anisotropy in magnetic resonance imaging, were found to be associated with the relative abundance of *Actinobacteria* phylum in the gut of healthy people (31). In addition, a randomized controlled trial reported healthy but stressed people to demonstrate improvement regarding perceived stress, anxiety, inflammatory markers and, notably, cognitive traits manifested by improved verbal learning and memory, as well as social emotional cognition, following probiotic supplementation (38).

Despite the presence of numerous positive individual studies, a recent systematic review with a meta-analysis found probiotic, prebiotic or well-defined culture-dependent fermented food to have a negligible but marginally significant effect on global cognitive functioning and did not support the use of such microbiota-targeting interventions for cognitive outcomes (48). As a result, further research is needed. In an era of increasing nutritional awareness (49, 50), research efforts may focus more on common but not necessarily standardized fermented food (kefir, yogurt, cheeses, sauerkraut, or other regionally pickled vegetables) (51, 52) and prebiotic-containing food (cereals, onions and some vegetables and fruits) (53, 54); these foodstuffs have not been well-studied in terms of their pro-cognitive effects. Remarkably, fermented food consumption alters the composition of gut microbiota and the host metabolome signature (55–57), also raising hopes for better brain function.

Modulation of the gut microbiota may beneficially affect not only cognitive function, but also mood and anxiety symptoms. Numerous interventional studies have found probiotic and prebiotic preparations to have somewhat positive overall effects on alleviation of depressive and anxiety symptoms in humans (58–63). Commonly-consumed fermented food has been also linked with lowering depressive (64) and anxiety (65) symptoms. As anxiety may deteriorate cognitive function (7, 9), it is possible that gut microbiota-based strategies may improve cognition by alleviating anxiety symptoms.

The aim of the present study was to investigate whether consumption of fermented food and food-derived prebiotics is associated with cognitive performance under stress in psychiatrically healthy medical students. If such a link was detected, the study would also assess whether these effects are mediated by depressive or anxiety symptoms. Otherwise, the study would have examined whether consumption of fermented food and food-derived prebiotics is associated with depressive or anxiety symptoms under stress.

## Materials and Methods

### Ethical Considerations, Study Design, and Data Anonymization

The study was approved by the Bioethics Committee of the Medical University of Lodz, Poland (RNN/111/20/KE, received on 02 April 2020). Expressing informed consent in an electronic manner was mandatory to take part in the study. The study protocol with the data analysis plan was prospectively registered on 04 June 2020 in the public repository Open Science Framework at https://osf.io/ny2vf/ (66); a few minor changes to the preregistered study protocol were made after the study commencement, which are presented and discussed in Supplementary Material 1.

**Figure 1:**
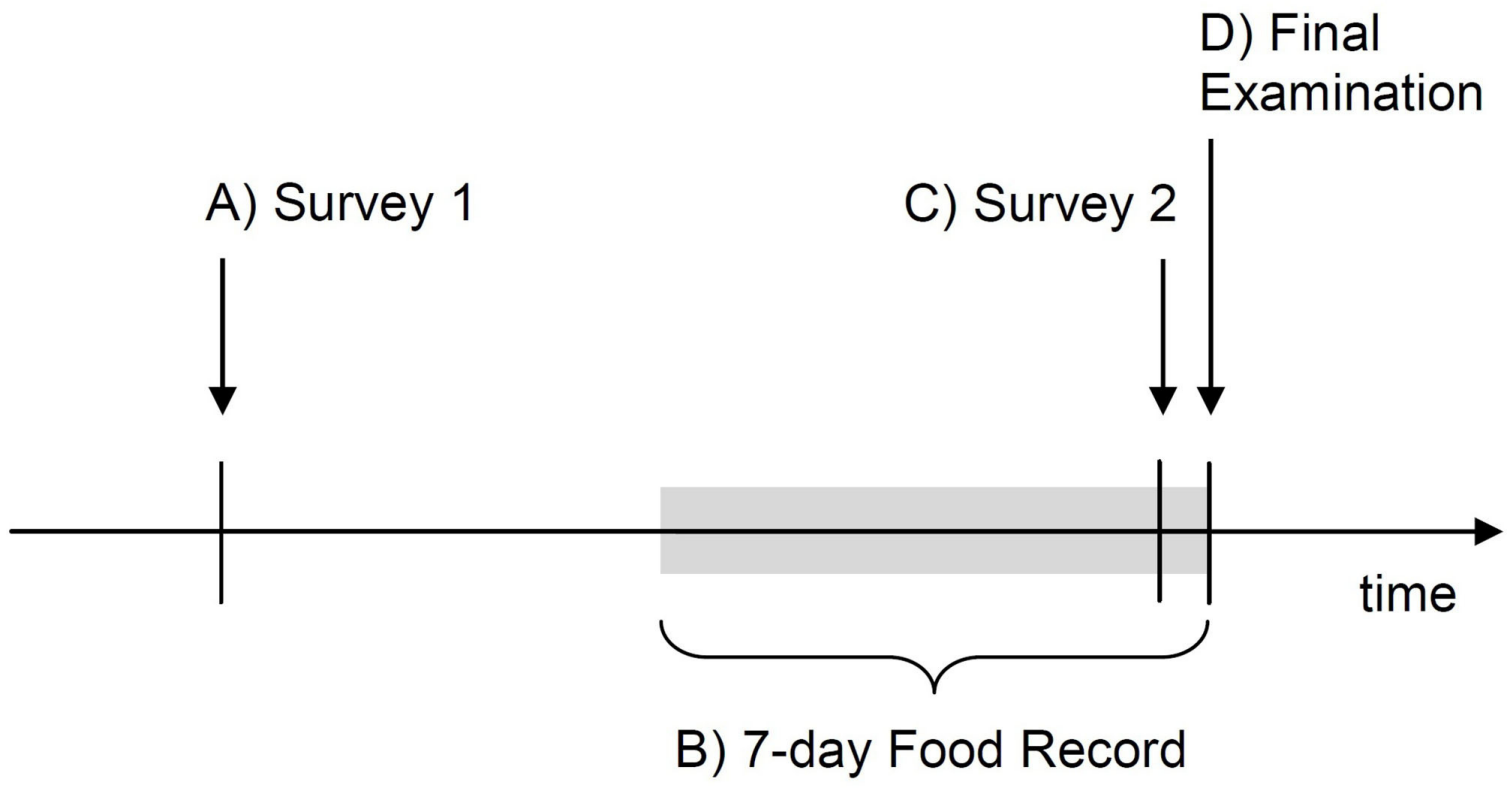
Timeline of the study procedures. Four steps of the study are indicated with letters A-D.

This was a prospective cohort study including a group of third-year psychiatrically-healthy medical students of the Medical University of Lodz, Poland. The study was questionnaire-based and was carried out online; no biological marker was assessed due to the COVID-19-related lockdown (67) that prevented biological samples collection. At the beginning of the study, the participants completed Survey 1 (Step A) to provide sociodemographic data and baseline characteristics. For the seven days preceding a final subject exam, which served as a model of real-life stress, consumption of selected foods and beverages was self-recorded (Step B) to calculate intake of fermented food and food-derived prebiotics (predictor variables). One day before the final exam, Survey 2 was completed (Step C) to assess depressive and anxiety symptoms (mediation or outcome variables), among other characteristics. Survey 2 also included a subject knowledge test with no academic consequences. The scores in the final exam (Step D), adjusted for pre-exam subject knowledge assessed a day before under unstressful conditions, were assumed to represent cognitive performance under stress (outcome variable). The study timeline is presented in Figure 1.

Volunteers were informed that the subject of the study was “Association between *lifestyle factors* and cognitive performance under stress.” The expression “consumption of fermented food and food-derived prebiotics” was replaced with a general term of “lifestyle factors” to not disclose the exact aim of the study and thus minimize possible observer bias. Students were also not informed about registration of the study protocol (66).

All the surveys and forms across the study were signed with a student identity card (ID) number so that they could be matched to the final exam scores. The final exam scores, including student ID numbers, were processed by a university administrator permitted to process student personal data who was not involved in the study; these results were extracted and handed to researchers. Hence, the researchers had no access to students' personal data other than ID numbers. After combining the data of surveys and forms with the exam results, student ID numbers were permanently removed from the final database. Volunteers were informed about this data anonymization procedure.

The present paper was outlined according to the STROBE (STrengthening the Reporting of OBservational studies in Epidemiology) guidelines for reporting cohort studies (68).

### Participants

The population of medical students was selected for this study as the group experiences high level of psychological stress and associated depressive and anxiety symptoms (69), which may interfere with their cognitive functioning. As a result, medical students were hypothesized to substantially benefit from consumption of fermented food and food-derived prebiotics with little “ceiling effect” (70).

Third-year medical students (Faculty of Medicine and Faculty of Military Medicine) of the Medical University of Lodz, Poland, were invited to participate. The only exclusion criterion was a formal inability to sit the first attempt of the final examination in Pharmacology (the model of stress). However, after study completion, the records of participants who declared being diagnosed with any psychiatric disease or taking any psychotropic drug (<3 months preceding the study), or these who took systemic antimicrobial agents or were hospitalized (<30 days before the start or during the study), were excluded from the analysis. These criteria were not stated as exclusion criteria to avoid indicating the exact aim of the study.

In order to demonstrate the effect size of β regression coefficient of 0.2 with a statistical power of 0.8 and statistical significance of 0.05, minimal sample size was estimated to be 193, as indicated by G^*^Power, version 3.1.9.2 (71). Assuming no more than 20% of drop-outs and no more than 20% of participant records being excluded due to above-mentioned reasons, the goal was to recruit at least 301 volunteers.

### Procedure and Research Instruments

Students were invited to join the study Microsoft (MS) Teams group (Microsoft; Redmont, WA, USA) to participate in the pre-study online meeting, to get any information relevant to the study (information for the participants, any training materials and study calendar) and to access the surveys. The study was carried out in four steps from A to D (Figure 1).

#### Step A

Survey 1 (Google Form; Google, Mountain View, CA, USA) was made available in the MS Teams group 1.5–3 weeks before the final exam in Pharmacology and could be filled not later than a week before the exam; each participant was asked to complete the survey once. The survey was to gather and evaluate some basal characteristics:

- general information:
  ◦ sociodemographic (sex, year of birth, socioeconomic status, number of inhabitants in a place of family residence),
  ◦ body mass and height (to calculate body-mass index, BMI);
- fact of suffering from chronic diseases (allergic, cardiological, endocrine/metabolic, gastroenterological, immune, infectious, neoplastic, neurological, and psychiatric) and taking psychotropic mediations;
- personality traits—assessed with the use of Polish version of the Big Five Inventory-Short (BFI-S) questionnaire (72, 73); BFI-S assesses five dimensions of personality with the use of three items to each, participants are asked to express the extent of their agreement with each of the items in a 7-point Likert scale;
- health-related behaviors:
  ◦ current cigarette smoking (including e-cigarettes use),
  ◦ physical activity—assessed as a general habit with the use of single-item 5-point semantic differential scale partially validated (74) against the Polish version of the International Physical Activity Questionnaire Short form (75, 76),
  ◦ general quality of diet—assessed with Polish version of the Starting the Conversation (STC) scale (74, 77); STC is composed of eight questions relevant to consumption of selected foods and beverages, participants indicate frequency of their consumption over several previous months in a 3-point Likert scale,
  ◦ mode of eating (ready-made *vs*. home-made products)—assessed as a general habit with single-item 7-point semantic differential scale—own tool;
- baseline depressive and anxiety symptoms—assessed with the use of Polish versions of the Patient Health Questionnaire-9 (PHQ-9) scale (78, 79) and the Generalized Anxiety Disorder-7 (GAD-7) scale (80, 81), respectively; the questionnaires could be validly applied on-line (82); PHQ-9 and GAD-7 assess mental health symptoms based on nine- and seven-symptom lists, respectively; the participants indicate frequency of experiencing each of the symptoms within previous 2 weeks with a 4-point Likert scale.

No forced answering option was used in any survey item. Survey 1 was pre-tested in a group of medical students not involved in the study and junior doctors (*n* = 6) and modified according to their feedback before the final version was constructed. A detailed description of the variables within the survey and operationalization of the constructs are available in the Study Protocol at https://osf.io/ny2vf/ (66). Screenshots from the original version of Survey 1 and its English translation are available in Supplementary Material 2.

#### Step B

The 7-day selective dietary record (83) was used to monitor pre-exam consumption of fermented and prebiotic-containing food. An electronic open-ended form of the Food Record (FR; Google Form) was constructed to gather information regarding consumption of 34 selected food items categorized in five classes. In this list, not only fermented and prebiotic-containing food items were included, but also many others to maintain the image that the study was examining “lifestyle factors” in general, and to thus minimize observer bias. Each of the 34 foodstuffs in the FR was described in detail and was illustrated with several photographs of different food portions or household measures, together with their masses in grams; this was intended to enable estimation of the mass of consumed food product to be entered to the FR form. Masses of food portions and household measures were established according to a Polish web-based dietary service: Ilewazy.pl (Edipresse Polska; Warsaw, Poland). The following 34 foods were included in the FR:

1. Meat, sausages, fish, seafood

a) red meat
b) white meat
c) fatty fish
d) other fish
e) fish oil
f) seafood

2. Dairy and eggs

a) milk
b) cottage cheese
c) cheese
d) yogurt, kefir, soured milk
e) eggs

3. Grain products

a) light bread
b) wholemeal bread, graham
c) cereal, groats, whole grain noodle
d) muesli
e) white rice
f) unpasteurised kvass and beer
g) wholemeal flour

4. Vegetables

a) potatoes
b) carrots, parsley, celery
c) beetroot
d) raw cucumber
e) pickled cucumber and pickling juice
f) cabbage
g) sauerkraut and pickling juice
h) other fermented vegetables and their pickling juice
i) onion, leek, garlic
j) leguminous vegetables
k) all other vegetables

5. Fruits and nuts

a) apples
b) citrus
c) bananas
d) all other fruits
e) nuts

The FR form was pre-tested in a group of medical students not involved in the study and junior doctors (*n* = 6) and modified according to their feedback before the final version was constructed. The form (both original and English translation) is presented in Supplementary Material 3.

During the pre-study online meeting, students were instructed on how to use the FR form to perform food mass assessment. Also, a few examples of food mass estimations were presented in a separate instructive document. A training version of the FR was made available in the MS Teams group to participants a few days before the true food record started.

Participants were instructed to complete the FR form each time they eat a meal or drink a beverage by entering the estimated mass (in grams) of each foodstuff consumed over seven days preceding the final exam. Seven-day period of FR was chosen as gut microbiota qualitative changes may evolve for at least 4 days following dietary shift (55, 84). As the seventh day of food recording was the day of the exam, participants were asked to record their food consumption until the moment of the start of the exam, but not the whole day (this made a total of about 6.25 days recorded). To improve adherence to food recordings, each day a funny picture story encouraging the participants to complete the FR was published in the MS Teams group. Although the method of recording individual meals in separate FR forms was highly recommended, in order to reduce the burden posed on participants (83) and lower possible loss to follow-up, 24-hour food recall using the same FR form was allowed as a last resort; in this case participants were asked to memorize (ideally by doing notes) food items consumed throughout the day and complete a single FR form at the end of the day. To remain in the study, a participants had to submit at least one FR form each day for at least five out of the 7 days. For the participants who missed FR for 1 or 2 days, the missing values were imputed: the quantity of food actually reported was multiplied by a factor corresponding to the number of missing days.

The fermented foodstuffs listed in the FR were: 2c (cheese), 2d (yogurt, kefir, soured milk), 3f (kvass and unpasteurised beer), 4e (pickled cucumber and pickling juice), 4g (sauerkraut and pickling juice), and 4h (other fermented vegetables and their pickling juice). The total mass of all the reported fermented foodstuffs consumed within the recording period was calculated for each participant and multiplied by 7/6.25 days (=1.12) to report the full 7-day consumption. The use of probiotic dietary supplements and medicinal products was also included in the measure of fermented food consumption (see details in Step C of the Materials and Methods section). Total consumption of fermented food served as a predictor in the analysis.

The prebiotic-containing foodstuffs listed in the FR that were used to calculate the total quantity of consumed food-derived prebiotics were: 3b (wholemeal bread, graham), 3c (cereal, groats, whole grain noodle), 3d (muesli), 3g (wholemeal flour), 4i (onion, leek, garlic), and 5c (bananas). Inulin and fructooligosaccharides (IN&FOS), naturally-occurring prebiotics with highly bifidogenic properties (53), were the only considered prebiotics in this study. IN&FOS consumption was estimated based on their food content, as reported by Moshfegh et al. (54). IN&FOS consumption was calculated by multiplying the quantity of each consumed prebiotic-containing food by a factor reflecting IN&FOS content, as presented in Supplementary Material 4. Following this, total IN&FOS consumption for each participant was multiplied by 7/6.25 days (=1.12) to report the full 7-day consumption. The intake of some rarely consumed prebiotic vegetables and use of prebiotic dietary supplements were also included in the measure of food-derived prebiotics consumption (see details in Step C of the Materials and Methods section). Total consumption of food-derived prebiotics served as a predictor in the analysis.

#### Step C

Survey 2 (Google Form) was made available in the MS Teams group a day before the final exam in Pharmacology and could be completed no later than 10 h before the exam; each participant was asked to complete the survey once. The survey was intended to gather and evaluate:

- pre-exam depressive and anxiety symptoms—assessed with the same PHQ-9 and GAD-7 measures as the basal symptoms reported in Survey 1; the instruction for completing the questionnaires was modified to assess the symptoms experienced over the previous seven days; pre-exam depressive and anxiety symptoms were to be used as mediators in the analysis or, in case of insignificant result for cognitive performance, as one of the outcome measures;
- some pre-exam health-related behaviors were examined using the same respective tools as in Survey 1, but the introductions were modified to assess the period of previous seven days:
  ◦ physical activity,
  ◦ general quality of diet (STC),
  ◦ mode of eating;
- 7-day consumption of some prebiotic-containing foodstuffs (asparagus, chicory root, dandelion leaves, globe artichoke and Jerusalem artichoke) as well as probiotic, prebiotic, and some other dietary supplements or medicinal products—assessed in any quantity units (mass, household measure units, number of doses) in open-ended questions; these products were not evaluated in FR because these vegetables are rarely consumed in Poland and the products are highly suggestive of the real aim of the study to the participants; consumption of these products multiplied by factors reflecting their IN&FOS content was added to the total IN&FOS consumption (Supplementary Material 4);
- adherence to 7-day FR—the questions were asked regarding the number of days in which: FR form was completed right after individual meals, whether paper notes were used for completing the 24-h (or shorter time) recall, inaccurate or selective reporting; moreover, participants were asked to indicate the method they used to estimate the foodstuff mass, e.g., weighing, using Ilewazy.pl service, using the illustrations presented in the FR, or doing it “by eye;” they were also asked to estimate the fraction of foodstuffs missed and not reported at all in the 7-day period;
- history of recent hospitalization and systemic antimicrobial drug use (<30 days before the start or during the study)
- pre-exam pharmacology test—the test comprised 40 yes/no questions; as it was not the formal academic assessment, it was assumed to reflect the actual pre-exam subject knowledge with no effect of stress; the test content was found highly relevant for the final examination by two independent academic pharmacology teachers not involved in the study.

No forced answering option was used in all the survey items. Survey 2 was pre-tested in a group of medical students not involved in the study and junior doctors (*n* = 6) and modified according to their feedback before the final version was constructed. A detailed description of the variables within the survey and operationalization of the constructs are available in the Study Protocol at https://osf.io/ny2vf/ (66). Screenshots from the original Survey 2 and its English translation are available in Supplementary Material 5.

The number of administered doses of probiotic and prebiotic dietary supplements or medicinal products, as assessed in Survey 2, was added to the total fermented food and food-derived prebiotic consumption evaluated with the 7-day FR, respectively. This was done based on an assumption that a single probiotic dose corresponds to 100 g of fermented food and a single prebiotic dose to 0.2 g of IN&FOS; these assumptions were legitimated by microbiological standards for fermented milk products (85) and characteristics of other fermented foods (52), as well as the mean probiotic and prebiotic content in some popular Polish dietary supplements (authors' own market analysis).

#### Step D

The final examination in Pharmacology was carried out without the influence of researchers. The exam was performed in a remote mode, in accordance with the guidelines in force during the first-wave COVID-19 lockdown (86). The exam consisted of 60 multiple-choice questions with five possible answers each; one point was awarded for each correct answer, no points were for an incorrect answer. The exam was available online through the Moodle platform (Moodle HQ; Perth, Australia). Additionally, the students were inspected visually and auditory by an academic staff (one teacher for 12–24 students) through MS Teams: the surroundings of randomly selected students were visually inspected before the exam started and the cameras and microphones of all the students needed to be turned on throughout the exam. Due to restricted stability of the electronic examination platform and the limited availability of teaching staff, the exam was performed in several rounds. Another set of questions was chosen for each exam round.

The points credited from the final exam in relation to the pre-exam test scores (Step C) and adjusted for the examination round constituted a measure of cognitive performance under stress—the primary outcome measure of the study. Such outcome measure has been used previously (87).

The final exam in Pharmacology for medical students of the Medical University of Lodz has previously been shown to evoke an anxiety response in terms of psychometrically measured state anxiety, hypothalamic-pituitary-adrenal axis activation and heart rate response (87), thus the exam could serve as an adequate model of real-life psychological stress (88). Furthermore, due to its remote nature and the COVID-19 first-wave lockdown that time, the event was likely even more stressful for students, as assessed in a pre-study interview (*n* = 7). In fact, the general anxiety level among Polish residents was found to be high during the first COVID-19 lockdown (80).

### Data Analysis

Data analysis plan was pre-specified in the Study Protocol available at https://osf.io/ny2vf/ (66). Missing values within the database were reported and imputed under a *missing at random* assumption with multivariate imputation by chained equations (89). General linear modeling (GLM) was used to evaluate the link between consumption of fermented food or food-derived prebiotics to cognitive performance under stress as expressed by the number of points granted in the final exam in pharmacology adjusted for pre-exam test scores and examination round. If any of these associations were statistically significant, mediation analysis would be performed including the pre-exam depressive and anxiety symptoms as potential mediating variables (90). Otherwise, association between the pre-exam depressive and anxiety symptoms with consumption of fermented food or food-derived prebiotics would have been evaluated. All the analyses were additionally adjusted for sex, socioeconomic status, number of inhabitants in a place of family residence, BMI, current cigarette smoking/use, overall diet quality (pre-exam STC score), physical activity, each of five personality traits (BFI-S), and morbidity (allergic, endocrine/metabolic, and gastroenterological diseases). Other analyses were treated as ancillary or exploratory. *P*-values below 0.05 were considered statistically significant. The analyses were performed using STATISTICA Software version 13.3 (Statsoft; Tulsa, OK, USA) and R Software version 4.0.0, package “mice” version 3.8.0 (R Foundation for Statistical Computing; Vienna, Austria). The underlying raw data has been made publicly available through the Mendeley Data repository (http://dx.doi.org/10.17632/kdxv4k6f5c.1).

## Results

### Study Timeline

The study commenced when Survey 1 was made available for participants; these dates were 04 June 2020 for students of Faculty of Medicine and 09 June 2020 for Faculty of Military Medicine. The final exam was carried out on 15 and 30 June 2020 at the respective Faculties. Survey 1 was completed in a median of 11 (1st−3rd quartiles: 10–12) days before the final exam.

### Volunteers, Dropouts, and Exclusions

Out of 597 eligible students, 490 volunteered for the study (82.1%). Of these, 46 participants dropped out during the course of the study (not providing food records for at least 3 days or not filling Survey 2) accounting for 9.4% of dropout rate. Among the 444 students who completed the study, 55 (12.4%) reported suffering from psychiatric disease or taking psychiatric medication for 3 months prior to enrolment, and a further 17 (4.4%) declared taking systemic antimicrobial medication or being hospitalized for 30 days prior to enrolment or during the study; these students were excluded. The final number of participants included in the analysis was 372; this number was sufficient based on minimal sample size estimation.

### Missing Data

Missing data comprised 67/34,596 (0.19%) of the values in the database. Survey 2 accounted for more missing values than Survey 1 (0.32 vs. 0.09%); this difference may be due to the survey burden experienced as a result of filling multiple food records, and the growing stress before the upcoming subject examination. Two variables in the database with the highest proportion of missingness were related to extent of adherence to food records asked in Survey 2 (both 8/372, 2.2% of missingness), followed by an STC item related to consumption of sweet drinks asked in Survey 2 (5/372, 1.3% of missingness). All the missing values were imputed before further analysis.

### Basal Characteristics of the Study Participants

According to their reports, the mean age of the 372 participants who completed the study was 22.7 years, with a little over one-third of them being male. The participants were relatively diverse in terms of their place of family residence, dietary quality and pattern and physical activity, but not so much in socioeconomic status. Of the participants, 12% reported being overweight (BMI ≥ 25 and <30), 2% obese (BMI ≥ 30) and 7% reported cigarette smoking or use. A little more than one-third reported suffering from a chronic disease, with the highest morbidity being allergic diseases, followed by endocrine/metabolic and gastroenterological. The overall state of mental health was worrisome, with more than two-thirds of the students reporting at least mild anxiety symptoms [GAD-7 score ≥ 5 (81)] and more than three-fourths at least mild depressive symptoms [PHQ-9 score ≥ 5 (78)]. Detailed characteristics of the study participants are presented in Table 1.

**Table 1:** Basal characteristics of the study participants (*n* = 372).

**Characteristics**	**Mean (standard deviation), median (1st−3rd quartiles), or absolute number (frequency)**
**Faculty**	
Faculty of Medicine	250 (67.2%)
Faculty of Military Medicine	122 (32.8%)
**Age**	
[*years*]	22.7 (1.1)
**Sex**	
Female	245 (65.9%)
Male	127 (34.1%)
**Socioeconomic status**	
Low	2 (0.5%)
Middle	264 (71.0%)
High	106 (28.5%)
**Number of inhabitants in a place of family residence**	
Below 5,000	88 (23.6%)
5,000–50,000	120 (32.3%)
50,000–500,000	91 (24.5%)
Over 500,000	73 (19.6%)
**Anthropometry**	
Body-mass index [kg × m^−2^]	22.0 (3.1)
**Chronic diseases**	
Allergic	89 (23.9%)
Cardiological	4 (1.1%)
Endocrine/metabolic	33 (8.9%)
Gastroenterological	21 (5.6%)
Immune	6 (1.6%)
Infectious	0 (0.0%)
Neoplastic	1 (0.3%)
Neurological	4 (1.1%)
Any chronic disease	128 (34.4%)
**Personality traits^a^**	
Neuroticism	12 (9–15)
Extraversion	12 (9–15)
Openness	15 (12–17)
Agreeableness	15 (12–17)
Conscientiousness	16 (14–18)
**Health-Related behaviors**	
Current cigarette smoking/use^b^	25 (6.7%)
Physical activity^c^	3 (2–4)
General quality of diet^d^	6 (5–8)
Mode of eating^e^	6 (5–6)
**Mental health**	
Depressive symptoms^f^	7 (5–11)
Anxiety symptoms^g^	6 (4–10)

a *Expression of five personality traits (Big Five Inventory-Short questionnaire) was presented numerically in the range of 3–21, with the midpoint of 12; Cronbach's alpha assessed in the study sample for the subscales was 0.65 for Neuroticism, 0.75 for Extraversion, 0.70 for Openness, 0.54 for Agreeableness, and 0.68 for Conscientiousness*.

b *Fraction of participants reporting either traditional cigarette smoking or e-cigarette use*.

c *Physical activity was expressed in the 5-point semantic differential scale from 1 (“I have no physical activity at all”) to 5 (“I play sport intensively 5 times a week”), with the midpoint of 3*.

d *General quality of diet was expressed as the number of points in the Starting the Conversation scale in the range of 0 (maximally healthy diet) to 16 (maximally unhealthy diet), with the midpoint of 8; Cronbach's alpha assessed in the study sample was 0.55*.

e *Mode of eating was expressed in the 7-point semantic differential scale from 1 (“I only eat products bought in a bar, restaurant, or ready-made products or snacks”) to 7 (“I only eat at home, home-made products”)*.

f *Depressive symptoms were evaluated with the Patient Health Questionnaire-9 scale; range 0–27; Cronbach's alpha assessed in the study sample was 0.79*.

g *Anxiety symptoms were evaluated with the Generalized Anxiety Disorder-7 scale; range 0–21; Cronbach's alpha assessed in the study sample was 0.89*.

### Pre-exam Health-Related Behavior

During the 7-day pre-exam period, the participants reported being significantly less physically active (*p* < 0.0001) than previously. Moreover, dietary practices were also modified, as revealed by the STC scale analysis. Although the mean number of total STC points did not significantly change (Wilcoxon signed rank test, *p* = 0.14), the scale demonstrated low internal consistency (Cronbach's alpha of 0.55 and 0.50 in basal and pre-exam assessment, respectively), indicating than each food category could be studied separately. The separate item analysis found that the consumption of vegetables (*p* = 0.0008), beans, chicken or fish (*p* = 0.0020) as well as margarine, butter or meat fat (*p* = 0.0072) significantly decreased in the pre-exam period. Also, the mode of eating significantly changed toward eating at home of home-made products (*p* < 0.0001).

### Pre-exam Mental Health and Subject Examination Scores

During the pre-exam period, students exhibited significantly more anxiety symptoms (median pre-exam GAD-7 score: 7, 1st−3rd quartiles: 4–12) than before (*p* = 0.0023); however, the median increase in the anxiety score was 0 (1st−3rd quartiles: −2–3). On the other hand, depressive symptoms did not significantly change (*p* = 0.17) in the pre-exam period (median pre-exam PHQ-9 score: 8, 1st−3rd quartiles: 4–12). Not much increase of mental health symptoms in the pre-exam period may suggest that depression and anxiety experienced by the students were more chronic and not simply attributed to the final exam.

In the test performed a day before the final exam to assess participants' subject knowledge, median score was 32 out of 40 points (1st−3rd quartiles: 30–34) (mean ± SD: 31.6 ± 3.9). In the final exam, the median score was 45.5 out of 60 points (1st−3rd quartiles: 41–51) (mean ± SD: 45.5 ± 6.8).

### Pre-exam Consumption of Fermented Food and Food-Derived Prebiotics

During the 7-day time period of pre-exam dietary monitoring a total of 7,904 valid FR were submitted by the participants who completed the study, which accounted for a median of 18 (1st−3rd quartiles: 13–22) FR forms for a person. Only 59 (15.9%) students skipped 1 or 2 days during dietary recording. As assessed in Survey 2, after 5 days of FR, 219 people (58.9%) reported recording FR directly after each consumed meal for at least 4 days, while 306 (82.3%) reported performing dietary recall (collective reporting of a few meals) for only no more than a single day. Also, 289 students (77.7%) reported omitting no more than 10% of consumed food, while only 18 (4.8%) omitted more than 20%.

The participants reported variable consumption of fermented food and food-derived prebiotics in the 7-day pre-exam period, as presented in Table 2. On average, the most widely-consumed categories of fermented food were “yogurt, kefir, soured milk” followed by “cheese;” together, these categories accounted for a mean of more than three fourths of total fermented foodstuffs consumption. Although the most consumed prebiotic-containing food category was “cereal, groats, whole grain noodle,” followed by “bananas” and “wholemeal bread, graham,” the most significant source of dietary prebiotic was “onion, leek, garlic,” accounting for a mean of more than a third of IN&FOS consumption.

**Table 2:** Pre-exam 7-day consumption of fermented and prebiotic-containing food, and food-derived prebiotics (number of subjects assessed = 372).

**FR item number**	**Food product**	**7-day food consumption [gram]**	
		**Median (1st−3rd quartiles)**	**Mean (standard deviation)**
**Fermented food**			
2c	Cheese	142.8 (56.0–236.6)	173.0 (151.8)
2d	Yogurt, kefir, soured milk	280.0 (47.6–560.0)	425.9 (511.4)
3f	Kvass and unpasteurised beer	0.0 (0.0–0.0)	52.4 (214.3)
4e	Pickled cucumber and pickling juice	0.0 (0.0–84.0)	72.8 (155.0)
4g	Sauerkraut and pickling juice	0.0 (0.0–0.0)	33.0 (98.9)
4h	Other fermented vegetables and their pickling juice	0.0 (0.0–0.0)	7.6 (58.0)
S2^a^	Probiotic dietary supplements or medicinal products^b^	0.0 (0.0-0.0)	5.9 (48.3)
	Total fermented food	609.7 (324.8–1,024.8)	771.4 (642.4)
**Prebiotic-containing food**			
3b	Wholemeal bread, graham	151.2 (0.0–392.0)	253.6 (294.4)
3c	Cereal, groats, whole grain noodle	280.0 (74.2–560.0)	407.5 (481.6)
3d	Muesli	0.0 (0.0–90.4)	72.0 (139.6)
3g	Wholemeal flour	0.0 (0.0–120.4)	87.6 (152.6)
4i	Onion, leek, garlic	67.2 (11.2–173.6)	127.6 (166.0)
5c	Bananas	179.2 (0.0–403.2)	267.3 (302.7)
S2^a^	Asparagus, chicory root, dandelion leaves, globe artichoke, and Jerusalem artichoke	0.0 (0.0–0.0)	46.7 (137.1)
	Total prebiotic-containing food	1,132.3 (708.9–1,625.1)	1,255.7 (778.6)
S2^a^	Prebiotic dietary supplements or medicinal products^c^	0.0 (0.0–0.0)	0.009 (0.094)
	Total IN&FOS prebiotics	25.6 (16.4–41.7)	30.9 (20.6)

*FR, food record; IN&FOS, inulin and fructooligosaccharides*.

a *Source of data: Survey 2*.

b *The result displayed after conversion to grams of fermented food (1 dose = 100 g of fermented food)*.

c *The result displayed after conversion to grams of IN&FOS (1 dose = 0.2 g of IN&FOS)*.

### Association Between Consumption of Fermented Food and Food-Derived Prebiotics With Cognitive Performance, Depressive, and Anxiety Symptoms Under Psychological Stress

**Figure 2:**
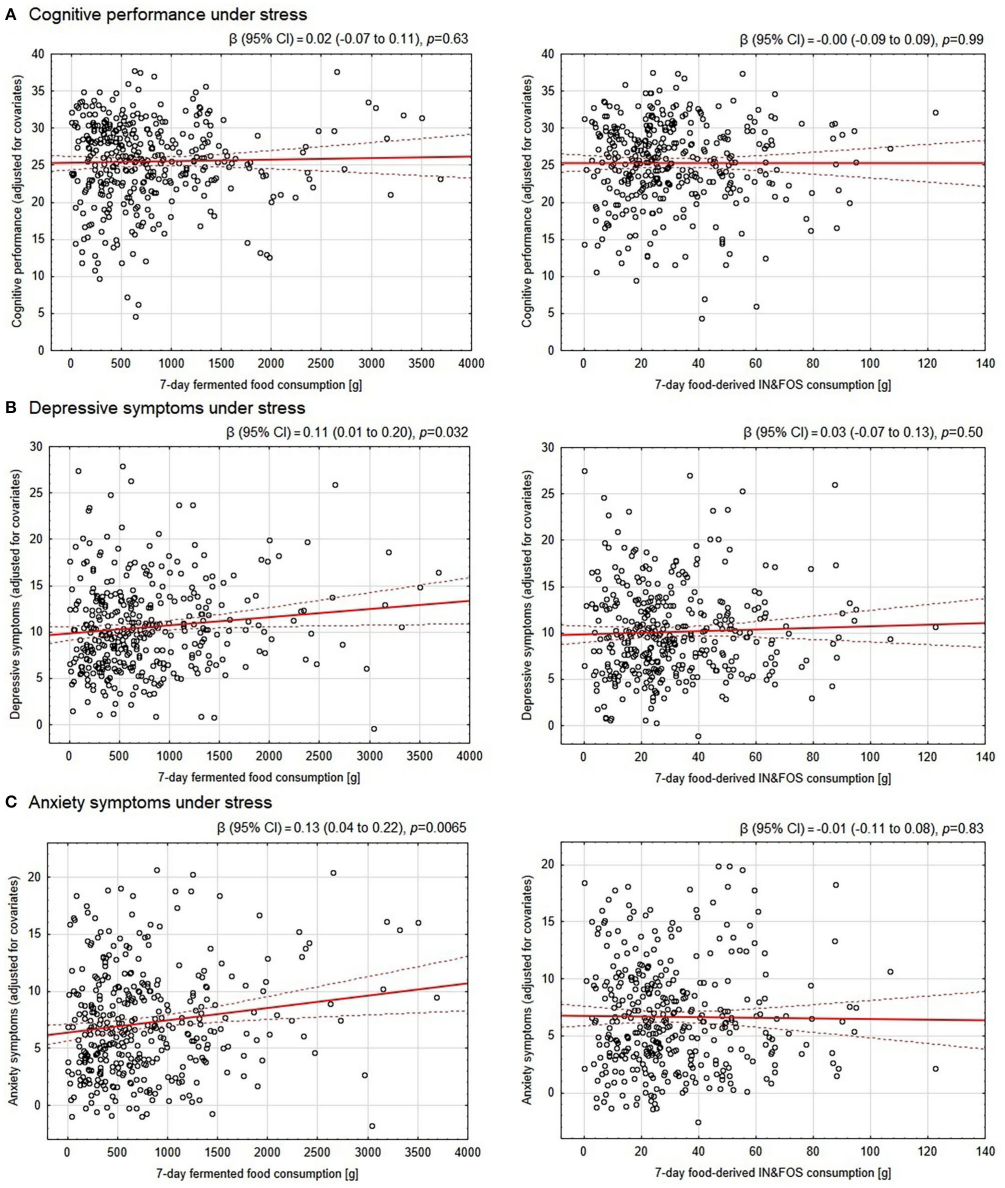
Association between the 7-day consumption of fermented food and food-derived prebiotics with cognitive performance, depressive and anxiety symptoms under psychological stress. The OY axes of the scatter plots represent: **(A)** scores in the final exam adjusted for subject knowledge assessed a day before the final exam under non-stressful conditions as well as the final exam round (cognitive performance under stress) and further adjusted for covariates, **(B)** pre-exam Patient Health Questionnaire-9 scores (depressive symptoms) adjusted for covariates, **(C)** pre-exam Generalized Anxiety Disorder-7 scores (anxiety symptoms) adjusted for covariates. The covariates included in the analyses were: sex, socioeconomic status, number of inhabitants in a place of family residence, BMI, current cigarette smoking/use, overall diet quality (pre-exam Starting the Conversation score), physical activity, each of five personality traits (Big Five Inventory-Short scales) and morbidity (allergic, endocrine/metabolic and gastroenterological diseases). The red solid line depicts the linear regression line, whereas the dotted ones indicate the 95% confidence intervals. IN&FOS—inulin and fructooligosaccharides.

Neither consumption of fermented food nor food-derived prebiotics was found to be significantly associated with cognitive performance under stress in either the raw or adjusted analyses. Therefore, no mediation analysis was performed. Nevertheless, our hypothesized link between fermented food and food-derived prebiotic intake and depressive and anxiety symptoms under stress was evaluated. Although the results were insignificant in the raw analyses, after adjustment for potential confounders, consumption of fermented food, but not food-derived prebiotics, was found to be significantly positively associated with the severity of pre-exam depressive and anxiety symptoms. The effect size for these associations was small however; only 1.3 and 2.1% of the variance in depressive and anxiety symptoms, respectively, as revealed by partial eta-squared, could be attributed to amount of fermented food consumed. Detailed results of the associations are presented in Table 3 and further illustrated in Figure 2.

**Table 3:** The association between consumption of fermented food and food-derived prebiotics with cognitive performance, depressive, and anxiety symptoms under psychological stress (*n* = 372).

**Product/substance consumed**	**β** **(95% CI)**, ***p*****-value, partial eta-squared**	
	**Raw analysis**	**Adjusted analysis^a^**
**Cognitive performance**		
Fermented food	0.02 (−0.07 to 0.10), *p* = 0.72, pη^2^ = 0.0%	0.02 (−0.07 to 0.11), *p* = 0.63, pη^2^ = 0.1%
Food-Derived prebiotics	−0.01 (−0.09 to 0.08), *p* = 0.88, pη^2^ = 0.0%	−0.00 (−0.09 to 0.09), *p* = 0.99, pη^2^ = 0.0%
**Depressive symptoms**		
Fermented food	0.08 (−0.02 to 0.18), *p* = 0.14, pη^2^ = 0.6%	0.11 (0.01 to 0.20), *p* = 0.032, pη^2^ = 1.3%
Food-derived prebiotics	−0.04 (−0.14 to 0.06), *p* = 0.44, pη^2^ = 0.2%	0.03 (−0.07 to 0.13), *p* = 0.50, pη^2^ = 0.1%
**Anxiety symptoms**		
Fermented food	0.08 (−0.02 to 0.18), *p* = 0.14, pη^2^ = 0.6%	0.13 (0.04 to 0.22), *p* = 0.0065, pη^2^ = 2.1%
Food-derived prebiotics	−0.10 (−0.20 to 0.00), *p* = 0.057, pη^2^ = 1.0%	−0.01 (−0.11 to 0.08), *p* = 0.83, pη^2^ = 0.0%

*pη^2^, partial eta-squared*.

a *Adjusted for sex, socioeconomic status, number of inhabitants in a place of family residence, BMI, current cigarette smoking/use, overall diet quality (pre-exam Starting the Conversation score), physical activity, each of five personality traits (Big Five Inventory-Short scales) and morbidity (allergic, endocrine/metabolic, and gastroenterological diseases)*.

### Sensitivity Analyses

The sensitivity association analyses performed in subgroups of participants who declared high adherence to the FR procedure, i.e., those missing no single day of food recording (*n* = 313) or no more than 10% of consumed foodstuffs (*n* = 289), were in line with the primary analyses. Fermented food and food-derived prebiotics consumption was not significantly linked to cognitive performance under stress. However, fermented food consumption was associated with depressive and anxiety symptoms in both the adjusted and raw analyses; the effect size of most significant associations exceeded 3%. Although the consumption of food-derived prebiotics was significantly negatively associated with pre-exam anxiety in the raw analysis in one of the subgroups, the association did not survive adjustment for covariates.

The sensitivity association analyses were also performed with the use of 7-day fermented food consumption calculated excluding probiotic dietary supplements and medicinal products. This was done in order to eliminate products with proven probiotic properties as the daily consumed fermented foods largely fall short of the definition of probiotic (91). The results obtained with such a modified measure of fermented food consumption were consistent with the original ones.

In addition, the associations of interest were tested with the use of pre-exam 3-day data on consumption of fermented food and food-derived prebiotics to account for the time period closest to the final exam and food consumption variability. The results were much in line with that of 7-day consumption. Fermented food and food-derived prebiotics intake during these three pre-exam days was substantially correlated with that of 7-day.

Detailed results of sensitivity analyses are presented in Supplementary Material 6.

### Ancillary and Exploratory Analyses

Exploratory analyses were performed to test whether the associations of interest were stable across multiple participant characteristics (such covariates as sex, BMI, physical activity, quality of diet, smoking, personality traits, depressive and anxiety symptoms, or subject knowledge in pharmacology). The analyses were carried out by evaluating the interactions between the covariates and 7-day consumption of fermented food and food-derived prebiotics in predicting cognitive performance, depressive and anxiety symptoms. None of the analyses indicated that the interaction has significant effect in predicting cognition, suggesting no link was present between the consumption of fermented food or food-derived prebiotics and cognitive performance under stress across the people of various characteristics. However, people declaring no physical activity at all in the 7-day pre-exam period and non-smokers exhibited higher effect size (up to 8% of variance explained) for positive link between consumption of fermented food and depression and anxiety. Moreover, people with lower than median *openness to experience*, as evaluated by the personality traits inventory, exhibited higher effect size (i.e., more than 5% of variance explained) for a positive link between consumption of fermented food and depression, but not with anxiety. Further detail is given in Supplementary Material 7.

Testing the 7-day consumption of each fermented and prebiotic-containing foodstuff separately for the association with cognitive performance under stress revealed that consumption of none of the products linked to cognition. On the other hand, high consumption of kvass or unpasteurized beer was significantly associated with severity of depressive and anxiety symptoms, whereas high consumption of asparagus, chicory root, dandelion leaves, globe artichoke and Jerusalem artichoke was linked to severity of anxiety. More detail is given in Supplementary Material 8.

## Discussion

Gut microbiota-based strategies have the potential to positively affect human cognition (16–19). Well-defined probiotic and prebiotic preparations and foodstuffs have been studied in this area (48), but little attention to date has been paid to fermented and prebiotic-containing foods that may be consumed on a daily basis but are not necessarily standardized. Even so, a link has been identified between such food intake and quality of gut microbiota (55–57). The present study applied the 7-day dietary record to determine the amount of fermented food and food-derived prebiotic consumption in medical students under psychological stress. The results generally indicate an insignificant link between consumption and cognitive functioning under stress; however, a positive association was found between the consumption of fermented food with severity of depressive and anxiety symptoms, with a small effect size.

It might be not surprising that consumption of fermented food and prebiotics derived from food does not appear to be associated with cognition. First of all, the studied population of psychiatrically healthy medical students, with no underlying cognitive impairment, although much stressed (69), may exhibit a “ceiling effect” (70) to their cognitive performance, which may be not much altered by pre-exam anxiety (8). In such situations, none of the factors could further boost cognitive outcome. Second, the fermented food evaluated in the present study, although containing potentially probiotic microorganisms (51), cannot be classified as such (91), and exhibits remarkably variable levels of microbial cells, ranging from none to more than a billion of colony forming units (CFUs) per gram of a foodstuff (52, 92). As a result, the evaluated consumption may not correspond to the intake of microorganisms. Third, the quality of microbes present in the examined fermented food has not been characterized, which could blur its health benefits even further (93). Similarly, prebiotic foods demonstrate variable contents of inulin and fructooligosaccharides (54) that may be affected e.g., by food processing (94). Moreover, fructooligosaccharides may produce relatively modest biomolecular alterations and mental health benefits compared to the more bifidogenic bimuno-galactooligosaccharides (95). Fourth, our results are in line with the overall negligible effect of probiotic, prebiotic and fermented food on cognition as revealed by a recent systematic review with meta-analysis (48). The cognitive outcome assessed in the present study was modeled based on academic examination performance under stress. The parameter was expressed as a subject final examination score in relation to the test score performed in non-stressful condition. This outcome is not related to single cognitive domain as it is characterized by knowledge retrieval under stress complicated by easy distraction, loss of coherent thoughts and difficulties in reading and understanding questions (8). As such, the applied cognitive measure is complex enough to not be affected by gut microbiota-related factors (48). Finally, the potential association between fermented food consumption and cognition might have been canceled by a possible relationship between high levels of fermented food consumption with severity of depressive and anxiety symptoms; this could in turn reduce academic performance as reflected by detrimental “test anxiety phenomenon” (8, 13).

Our data indicates that high consumption of fermented food may be associated with more severe depressive and anxiety symptoms. The link is likely independent of overall diet quality and other potentially confounding factors. This result is in contrast with the majority of probiotic intervention studies (58–63) and correlational research assessing the frequency of fermented food intake (64, 65). Although such result is intriguing, it is not completely isolated. Wolfe et al. (96) found higher consumption of cheese to be associated with an increased risk of depressive symptoms in men, but not in women, and Yu et al. (97) found very frequent yogurt consumption to be associated with increased depressive symptoms in a small Chinese population of adults. If the finding of a positive link reported herein between consumption of fermented food and depressive and anxiety symptoms is not simply a result of type I error, it calls for explanation. The discovered association may reflect two possible causality directions (not to mention more complicated interrelations): either consumption of fermented food contributes to depressive and anxiety symptoms or the students presenting these mental health problems tend to eat more fermented foodstuffs.

The former possibility has some literature support. Although probiotic preparations present some mental health benefits (58–62), fermented food is not necessarily probiotic (91). Fermented food may contain pathogenic microorganisms and viruses with opposite roles, and the adverse effects of fermented food may be under-reported (98). A portion of fermented dairy products, even these manufactured from pasteurized milk in developed countries, are of unsatisfactory microbiological quality. In a study from United Kingdom, 2% of a large sample of retail cheeses made of pasteurized milk failed to adhere to European Commission recommendations and contained considerable counts of *Staphylococcus aureus* and *Escherichia coli* (99); the presence of the latter may be indicative of fecal contamination (100). Traditionally-made products could be of much worse microbiological quality, with up to a third of them reported to contain potentially pathogenic bacteria (101, 102). Fermentation processes may also lead to the accumulation of unwanted microbial metabolites such as bacterial and mycotoxins, and biogenic amines. These problems led to sporadic foodborne infection outbreaks (93, 103, 104). This is unlikely that participants of the current study collectively experienced food poisoning, however, some of them could be exposed to small amounts of deleterious microbes and their metabolites. This is possible particularly as the weather was the enemy of food quality in this study: the research was performed in June and temperatures at that time were relatively high in central Poland (105). Exposure to fermented food-derived toxins could have evoked a low-level inflammatory response (106–109). Notably, both “dietary inflammation” (110) and foodborne infections (111) have been suggested to adversely affect mental wellbeing by evoking excessive depressive and anxiety symptoms.

The latter assumption, that increased fermented food consumption occurs as a result of a depressive and anxiety state secondary to psychological stress, is also possible. Psychological stress-induced glucocorticoids release can increase appetite and energy intake (112). In an interventional study, women highly reactive to stress in term of salivary cortisol response consumed more calories after exposure to stress and presented more preference for sweet food (113). Another human interventional study found more intake of sweet and fatty foods following exposure to a stressor (114). In terms of fermented food consumption the evidence is very limited and restricted to cross-sectional study design (97, 115), making it impossible to establish a cause-and-effect relationship. However, a Polish study found female patients suffering from clinical depression to consume more cheese (but not yogurt) than healthy controls (116). In most patients in their study, the diagnosis of depression was set long enough before the consumption was assessed to consider dietary behavior secondary to depression; however, the study tested multiple hypotheses with no adequate correction to control false discovery rate, which could imply simply a false-positive finding (116). As psychological stress may redirect food preferences to sweet and high-energy foodstuffs in stress-reactive people (113, 114), consumption of yogurt could increase as many such products contain a substantial amount of sugar or artificial sweeteners (97). Also, the intake of cheeses, which are highly caloric products, could grow although no preference for salty flavor following stress exposure was noted (113). A shift to cheese consumed as a snack could also occur simply as a result of lack of time experienced by anxious participants to prepare and consume a full meal (117). Finally, whatever the direction of causation, the main analysis of association between fermented food intake and depressive and anxiety symptoms in our study yielded a small effect size that explained no more than 2% of variability in mental health; thus it likely presents negligible overall impact at the populational level.

It appears as all the tested fermented food categories (cheese, fermented milk products, kvass, pickled vegetables) contributed to the positive association between total consumption of fermented food and depressive and anxiety symptoms: the regression coefficients were positive for all instances apart from probiotic dietary supplements or medications (Supplementary Material 8). The exception for the latter may suggest that “pure probiotics,” without the discussed above potential admixtures of harmful metabolites, may be at least neutral regarding mental health. In turn, further studies may benefit from differentiating probiotics from fermented food, particularly the so-called *wild* or *uncontrolled* ferments. On the other hand, kvass and unpasteurised beer were the only fermented foodstuffs whose consumption to demonstrate a statistically significant and consistent positive correlation with both depressive and anxiety symptoms; this could result from particularly low microbiological quality and possible contamination of the products (118). In fact, the microbiological safety criteria for kvass production have not been established (119). Moreover, it cannot be excluded that some participants may have mistakenly reported any beer (or even any alcoholic beverage) in the food record category of “kvass and unpasteurised beer.” Indeed, considering that the propensity of alcohol consumption to manage psychological stress predicts depression and anxiety symptoms (120, 121), it would not be surprising to see a relatively strong association beween “kvass and unpasteurised beer” consumption and deterioration of mental health. Assuming incorrect reporting in the category of “kvass and unpasteurised beer,” total fermented food was recalculated with the exclusion of this category (Supplementary Material 8). Even so, this modified analysis was significantly positively associated with anxiety symptoms, and presented a trend toward association with depressive symptoms, thus supporting the robustness of the obtained results for fermented food.

The analysis of interactions between consumption and covariates revealed that the positive association observed between consumption of fermented food and depressive and anxiety symptoms persisted in many subgroups of participants (Supplementary Material 7). However, physical activity, cigarette smoking and personality trait of openness to experience appeared to modulate the extent of reported association. The association between fermented food consumption and symptoms of depression and anxiety was stronger in people reporting no physical activity in the assessed 7-day time period and in non-smokers. On the other hand, an insignificant association was observed for physically-active and smoking participants. This may suggest physical activity and smoking to protect from the deleterious effect of fermented food on mental health. In fact, physical exercise (122) and exposition to nicotine (123, 124), which is present in tobacco smoke, were found to exhibit anti-inflammatory properties. Consequently, exercises and nicotine could reverse the pro-inflammatory action of fermented food-derived toxins, canceling their potential detrimental effect of mental health, as discussed above. Similarly, people with low *openness to experience* presented a relatively strong positive association between consumption of fermented food and symptoms of depression, but not anxiety, whereas no such link was found for people who were highly open to experience. Openness may have a protective influence against depression (125) by modulation of psychosocial (126, 127) as well as health-related (128, 129) behaviors and beliefs. As the analysis reported in the present research was adjusted to some health-related behaviors (physical activity and general dietary quality), the psychosocial mediators may provide protection from depressive symptoms in fermented food consumers highly open to experience. Nonetheless, the discussed analysis of interactions between consumption and covariates was exploratory and no correction was made for multiple hypothesis testing. As the reported results are uncertain, the proposed interpretation may be speculative and should be regarded with caution.

The present study has both strengths and limitations. First, the major advantage is that it used a prospectively-registered study protocol with detailed data analysis plan (66). Adhering to this document allowed to avoid data-driven analysis, assured proper interpretation of statistical significance and provided research transparency and validity (130). Second, many previous papers reporting the relationship between fermented food consumption and mental health-related outcomes used food frequency questionnaires (FFQ) to assess dietary intake (64, 65, 96, 97, 116, 131, 132). In contrast, the present research adopted a food recording method, and it appears the only study in this area to use such an approach. Food records outweigh FFQ by providing accurate and quantitative measurement of individual foodstuff consumption (83), assuring high-quality data reporting. This might be a reason why our present findings are distinct from those of many FFQ-based studies. Third, when the volunteers were invited to participate in the study, its exact aim was not disclosed. The study was referred to throughout the research procedures as evaluating *lifestyle factors*, but not fermented and prebiotic foods. Not revealing the actual focus on gut microbiota-related foods likely reduced the observer bias (133) and further contributed to accuracy of food intake measure.

One limitation of the study is its correlational character, which prevented causative analysis. In order to reliably establish direction of causation, interventional controlled settings need to be applied. However, such a study design could be very challenging when dealing with commonly-consumed food in terms of identifying an appropriate placebo form and blinding procedure (134). Instead, the present study applied adjustment for several potential confounders such as sociodemographic characteristics, personality traits, anthropometry, morbidity, cigarette smoking, physical activity and overall diet quality. This allowed for less biased estimation of associations of interests. Second, monitoring of fermented food and food-derived prebiotics intake was based on frequently-consumed foodstuffs, and not all suitable food products were included in the food record questionnaire. For example fermented meat products were not covered due to their low consumption in Poland (135) and thus the risk of incorrect classification by the participants. Moreover, cottage cheese was not included; despite being made with the help of lactic acid bacteria, it is heat-treated at the final step of its production (136) and likely contains little if any live bacteria. However, a similar product, curd cheese, which is relatively frequently consumed in Poland (137), likely contains live bacteria due to its production technology (138), but no separate category was made for this foodstuff. Thus, curd cheese consumption was not included in the sum of fermented foods. Additionally, although the analysis was adjusted for general diet quality, which included sweet consumption, the intake of this foodstuff category was not monitored in detail. However, sugar consumption has been associated with lower gut microbiota quality (139). Third, the measure of cognitive functioning applied in this study was not a validated test. Although the use of validated measures is encouraged (48), the results of such tests not necessarily predict everyday cognitive performance due to existence of compensatory mechanisms (140), and more real-life approach to cognitive testing is being advocated (141). As a result, we employed a real-life cognitive proxy based on academic performance. By using such measure, we also hoped to obtain the results more applicable to academic practice. Nonetheless, failure to include any validated measure of cognitive performance under stress prevented between-studies comparison and should be considered a limitation. Fourth, the present study did not investigate dietary-relevant alterations in gut microbiome and metabolome. Such analysis could provide mechanistic explanation of the reported phenomena. The rationale for the study was based on the assumption that fermented food consumption alters gut microbiota. Such assumption is legitimate, however, based on the available literature (55–57). In general, the study provides high-quality data challenging the hypothesis of a beneficial link between consumption of fermented food and food-derived prebiotics with mental health.

## Conclusion

In psychiatrically healthy medical students under psychological stress, consumption of fermented food appears not to be associated with cognitive performance, as evaluated by scores in a stressful subject examination adjusted for subject knowledge. However, high consumption of fermented food may be associated with more severe depressive and anxiety symptoms experienced before a stressful event. Consumption of food-derived prebiotics seems to be related to neither cognitive performance, depressive nor anxiety symptoms under stress. Further research is needed: to evaluate the reported findings, to reveal the direction of causality between the tested phenomena, to determine the link in patients suffering from clinical depression and to clarify safety issues associated with fermented food.

## Data Availability Statement

The original contributions presented in the study are included in the article/Supplementary Materials. The underlying raw data has been made publicly available through the Mendeley Data repository (http://dx.doi.org/10.17632/kdxv4k6f5c.1).

## Ethics Statement

The studies involving human participants were reviewed and approved by Bioethics Committee of the Medical University of Łódź, Poland. The participants provided their electronic informed consent to participate in this study.

## Author Contributions

MSK: conceptualization, formal analysis, investigation, data curation, visualization, and project administration. MSK and ŁM: methodology and resources. MSK and EK: validation, funding acquisition, and supervision. MSK, MD, and MK: writing—original draft preparation. MSK, ŁM, MD, and MK: writing—review and editing. All authors have read and agreed to the published version of the manuscript.

## Funding

The paper has been supported by the Medical University of Lodz with the Grant No. 503/5-108-03/503-51-001-19-00 (received by EK).

## Conflict of Interest

The authors declare that the research was conducted in the absence of any commercial or financial relationships that could be construed as a potential conflict of interest.

## Publisher's Note

All claims expressed in this article are solely those of the authors and do not necessarily represent those of their affiliated organizations, or those of the publisher, the editors and the reviewers. Any product that may be evaluated in this article, or claim that may be made by its manufacturer, is not guaranteed or endorsed by the publisher.
