# Supplementary material for: Association Between Consumption of Fermented Food and Food-Derived Prebiotics With Cognitive Performance, Depressive, and Anxiety Symptoms in Psychiatrically Healthy Medical Students Under Psychological Stress: A Prospective Cohort Study
Source: Front Nutr. 2022 Mar 3;9:850249. doi: 10.3389/fnut.2022.850249 (PMC8929173; doi:10.3389/fnut.2022.850249)
Supplement: Supplementary file 1 [file Data_Sheet_1.DOCX]

***Supplementary Material 1***

***Modifications in the study protocol made after the study commencement***

The following modifications in the study protocol after the study commencement (04 June 2020) were done:

| No | Record in the original protocol | Modification | Reason for modification |
| --- | --- | --- | --- |
| 1 | Study protocol title: “Association between consumption of fermented and prebiotic-containing food and cognitive performance under stress in healthy volunteers” (pg. 1). | Manuscript title: “Association between consumption of fermented food and food-derived prebiotics with cognitive performance, depressive and anxiety symptoms in psychiatrically healthy medical students under psychological stress: a prospective cohort study” | The modification was done to better reflect the authors’ intention expressed in the Study Protocol, but not to change the original ideas:  - The study was to investigate intake of “food-derived prebiotics” rather than “prebiotic-containing food” (pg. 10).  - Not only “cognitive performance under stress” was to be evaluated. Depressive and anxiety symptoms were to be included as mediators (pg. 11). However, as cognitive performance was found to have insignificant effect, mediation analysis was useless, and depression and anxiety were evaluated as outcome variables.  - “Psychiatrically healthy” participants were to be examined, but not “fully healthy people” as expressed by exclusion criteria (pg. 9).  - The results could not be generalized to “young adults” but rather to “medical students”. |
| 2 | Steps of the study:  “7-14 days before the final exam participants will complete the 1^st^ Survey” (pg. 2 and 5). | For students of Faculty of Military Medicine the study started 21 days before the final exam. | Some students began the study earlier due to administrative reasons, what was more coherent with authors’ original plan: “1^st^ Survey (…) was originally planned to be performed 14-28 days before the final [exam], but administrative disturbances related to final exam formatting in COVID-19 pandemics occurred, and the study begins with delay” (pg. 5). |
| 3 | Figure. Timeline of the study (pg. 3):  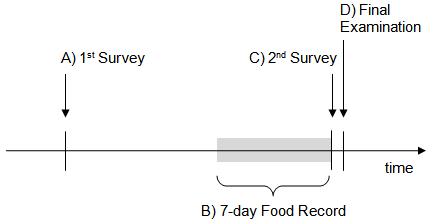 | Step B finished just before the final exam, but not before 2^nd^ Survey as presented in the original figure:  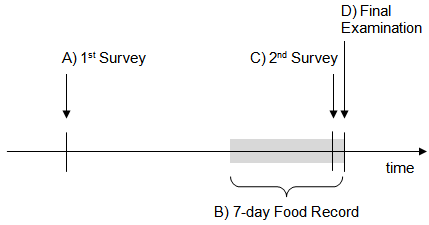 | Mistake in the Study Protocol, not according to the researchers’ intention. |
| 4 | A question to be asked in Survey 1: “Number of inhabitants in a place of residence” (pg. 4). | The content of question was in fact: “Number of inhabitants in a place of *family* residence”. | Mistake in the Study Protocol, not according to the researchers’ intention. |
| 5 | “Participants will be asked to record their food diary covering 7 days preceding the final examination in Pharmacology (including day of the exam)” (pg. 5). | In the day of the exam, participants were asked to record only their pre-exam food intake. As the exam was carried out at the morning, total food record lasted about 6.25 days. In order to estimate 7-day food consumption, total intake of each food product in all participants was multiplied by a factor of 7 days / 6.25 days (=1.12) | To get a more accurate estimate of 7-day food consumption. |
| 6 | “2. Dairy  (…)  d. yogurt, kefir, curd” (pg. 6). | It should be “yogurt, kefir soured milk” | The foodstuff “soured milk” was incorrectly translated to “curd” in the Study Protocol. |
| 7 | “Step 3 – 2^nd^ Survey” (pg. 7) – does not include the Starting the Conversation scale and the “way of eating” question. | Survey 2 also included the Starting the Conversation scale and the “way of eating” question. | To monitor overall pre-exam dietary behavior, to compare this behavior to basal and to include diet quality as a covariate in data analysis. |
| 8 | A question to be asked in 2^nd^ Survey: “Extent of adherence to food records – assessed as single-item 7-point semantic differential: from “definitely yes” to “definitely no” – operationalized as a 7-level ordinal variable” (pg. 8). | There were nine questions (but not a single one as originally planned) asked in Survey 2 related to extent of adherence to food records. | To get more extensive information regarding the extent of adherence to food records among the participants. |
| 9 | Missing data management plan: “The missing data will be managed in the following way:  a) The fraction of missing values for each variable will be calculated. (…)  c) Pattern of data missingness will be assessed” (pg. 9). | The fraction of missing values was reported only to the variables with the highest proportion of missingness. Pattern of data missingness was not assessed. | Fraction of missing values was relatively low. As a result disclosing all the variables with their missing values and examining the pattern of data missingness could be redundant and useless. |
| 10 | “Optional adjusting factors – potential confounders (that may be included in the analyses):   - sex - BMI - any current cigarette smoking/use - sociodemographic status (treated here as continuous variable) - number of inhabitants in a place of residence (treated here as continuous variable) - physical activity (treated here as continuous variable) - each of 5 personality traits” (pg. 11). | - “Sociodemographic status” was meant by the authors as “socioeconomic status”.  - Apart from the indicated adjusting factors, morbidity with more than 10 cases (allergic, endocrine/metabolic, gastroenterological diseases) and overall pre-exam diet quality (assessed with the use of Starting the Conversation questionnaire applied in the Survey 2) were used as covariates. | To obtain more covariate-independent estimation of association coefficients. All additionally included parameters are potential confounders by definition as they may affect both the predictors (consumption of fermented food and food-derived prebiotics) and outcome (cognitive performance under stress, depressive and anxiety symptoms). |

Quotations and pgs indicated in the table refer to the pre-registered Study Protocol available at: <https://osf.io/ny2vf/>
