# Supplementary material for: Association Between Consumption of Fermented Food and Food-Derived Prebiotics With Cognitive Performance, Depressive, and Anxiety Symptoms in Psychiatrically Healthy Medical Students Under Psychological Stress: A Prospective Cohort Study
Source: Front Nutr. 2022 Mar 3;9:850249. doi: 10.3389/fnut.2022.850249 (PMC8929173; doi:10.3389/fnut.2022.850249)
Supplement: Supplementary file 2 [file Data_Sheet_2.DOCX]

***Supplementary Material 2***

***Survey 1***

The screenshots of the original survey are presented below.

**
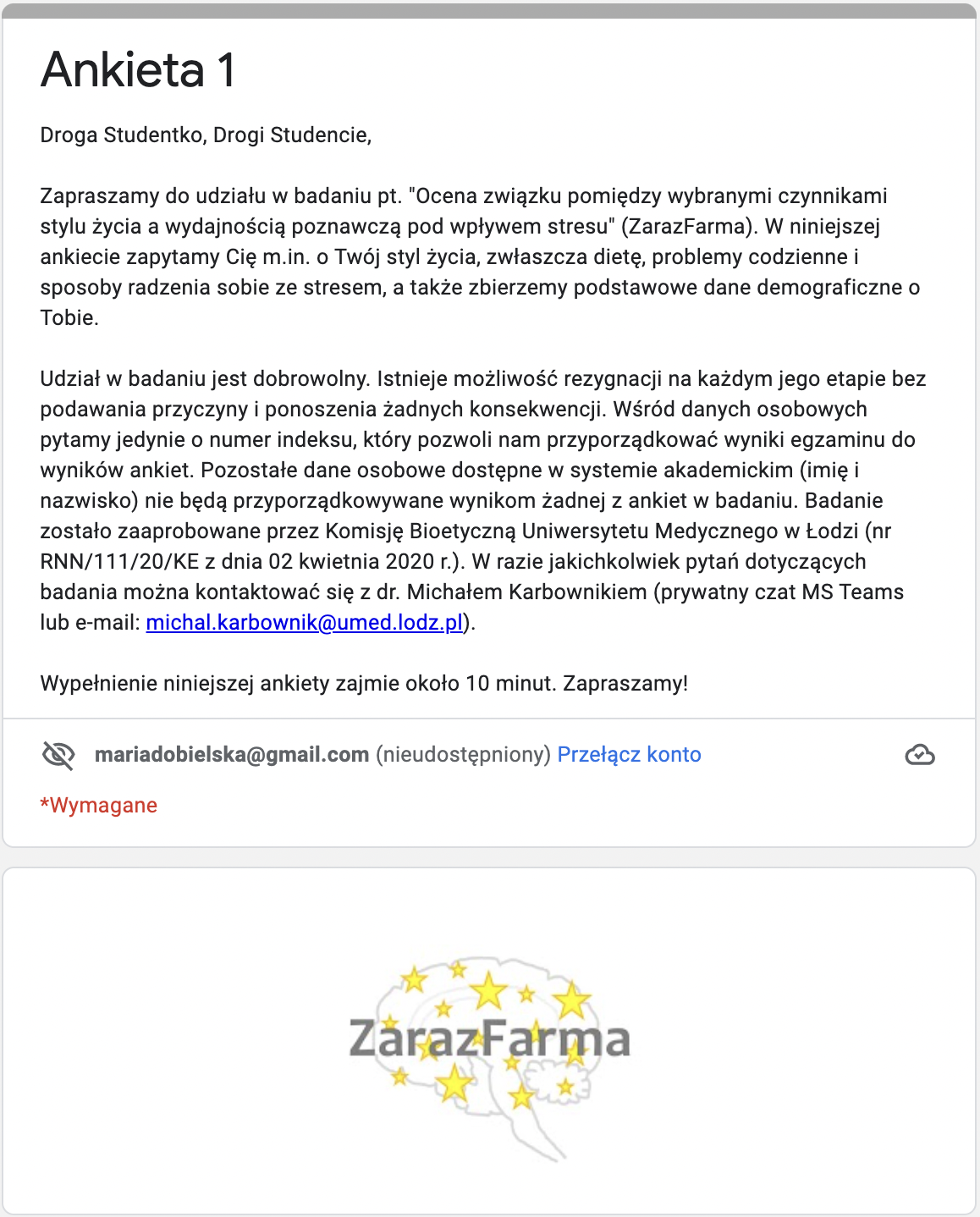
**

**
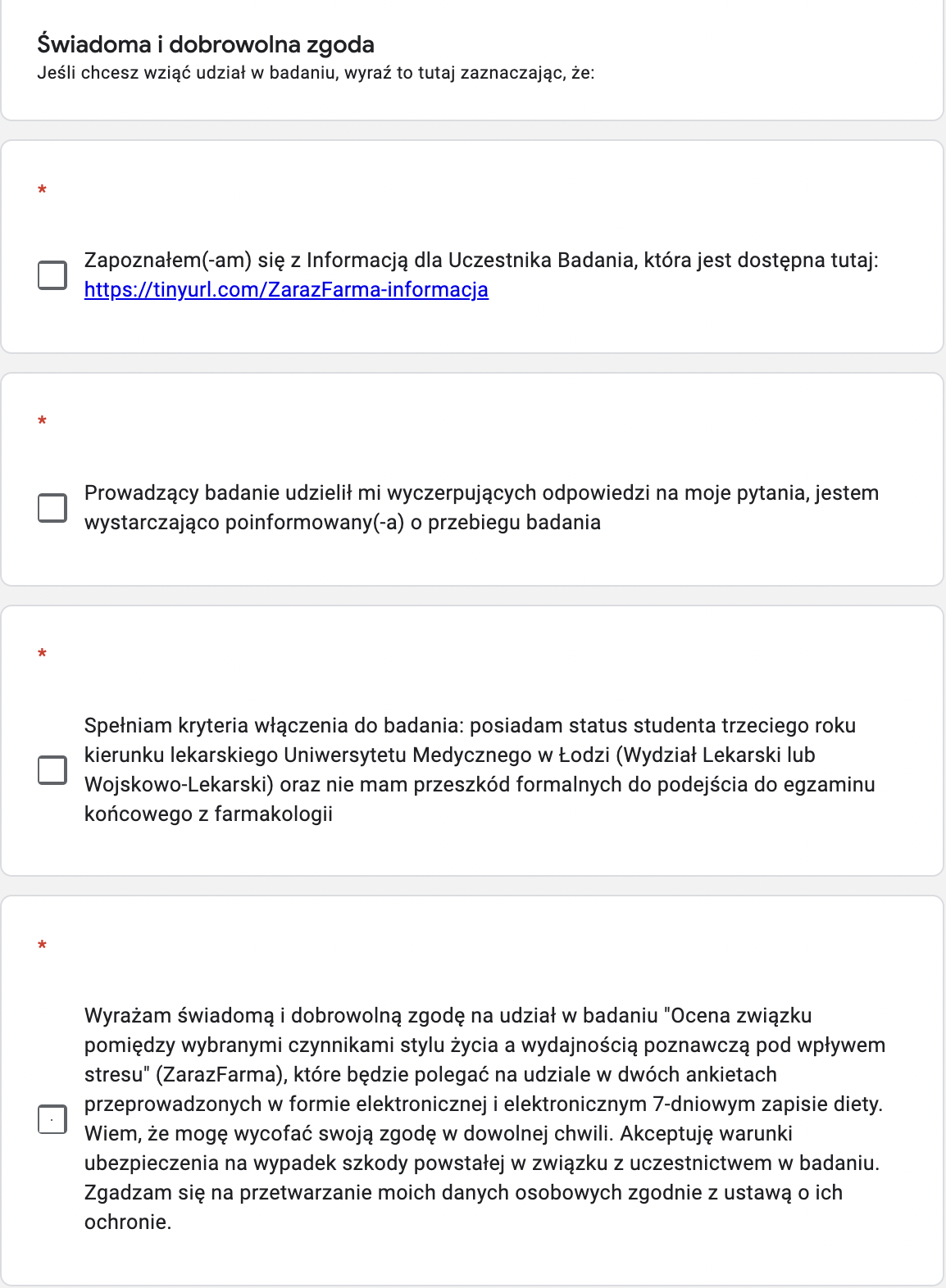
**

**
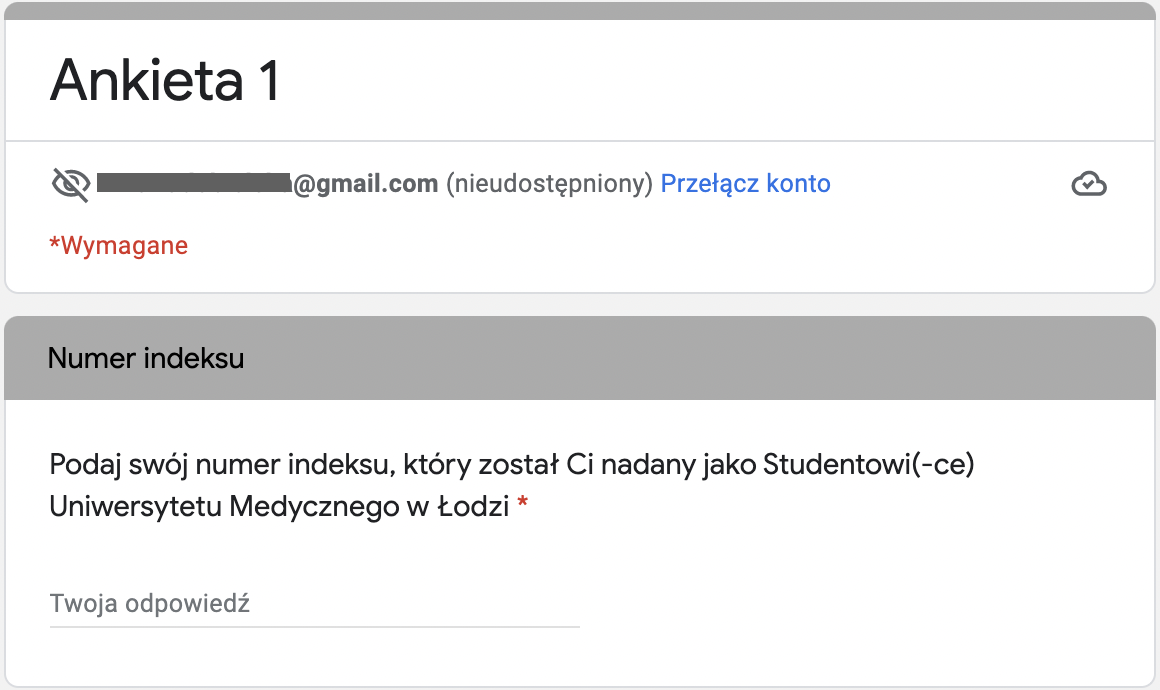
**

**
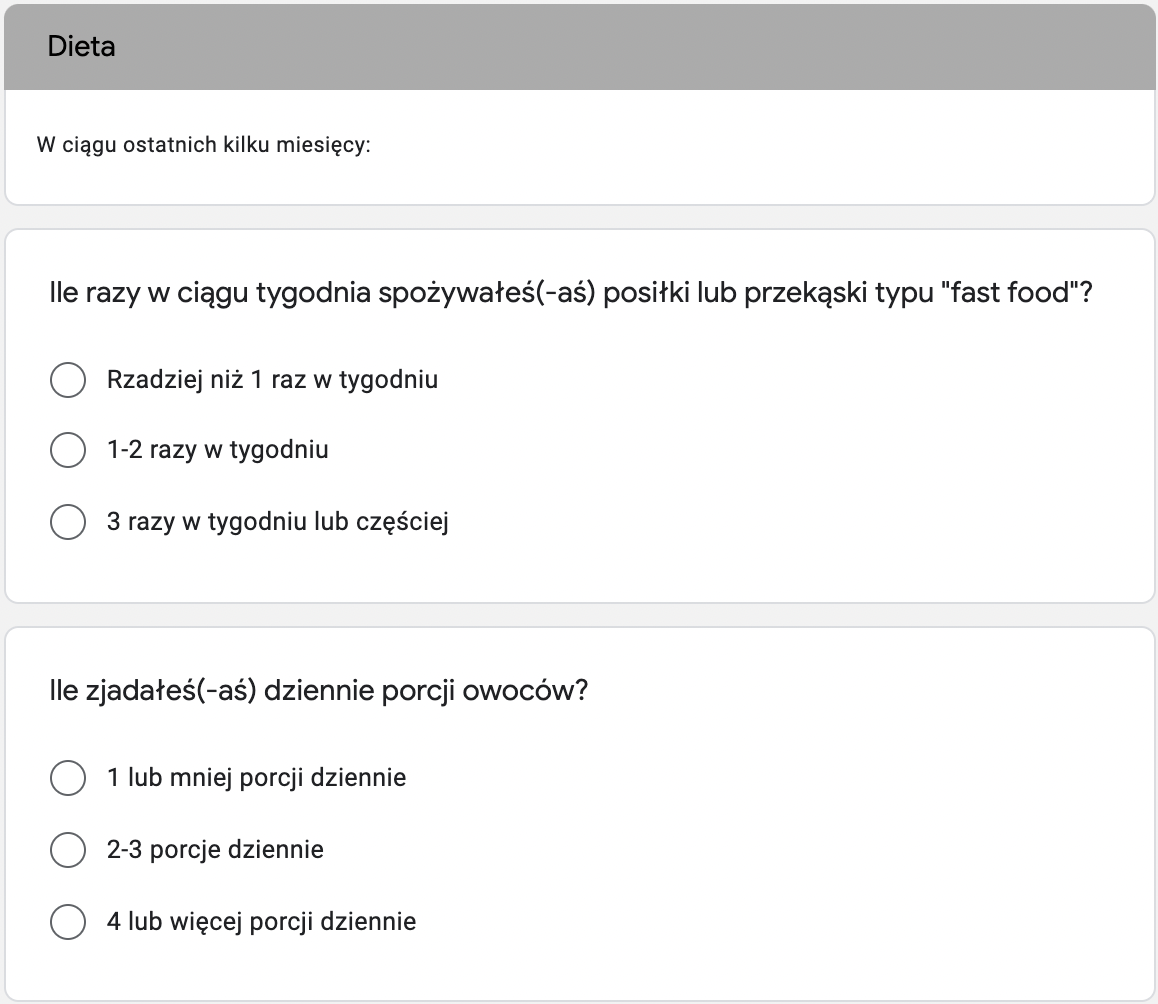
**

**
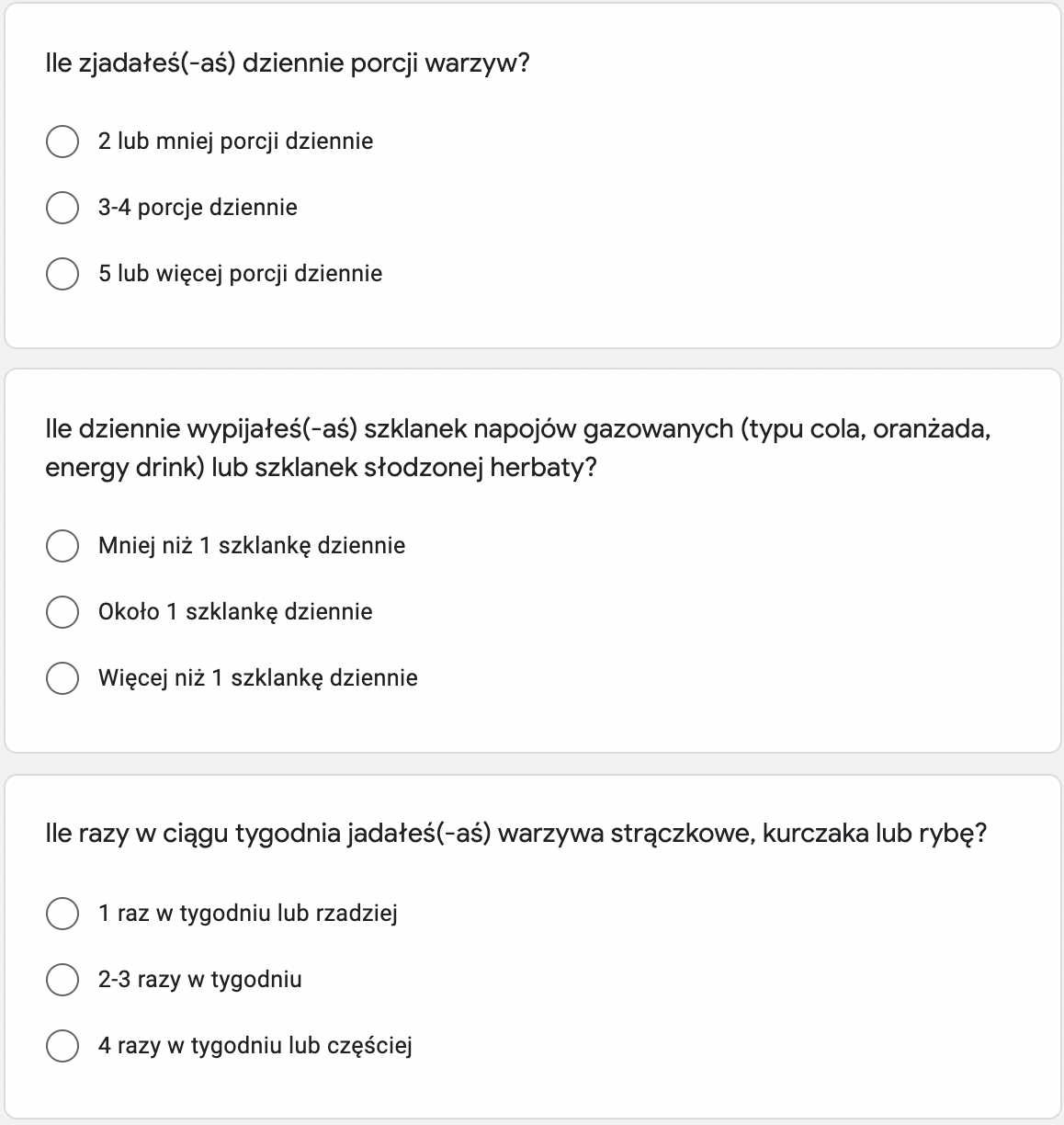
**

**
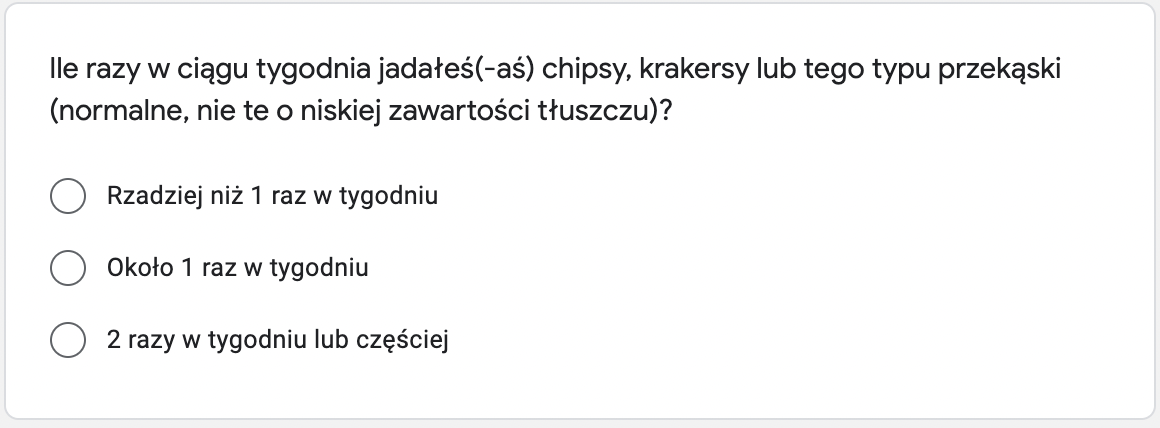
**

**
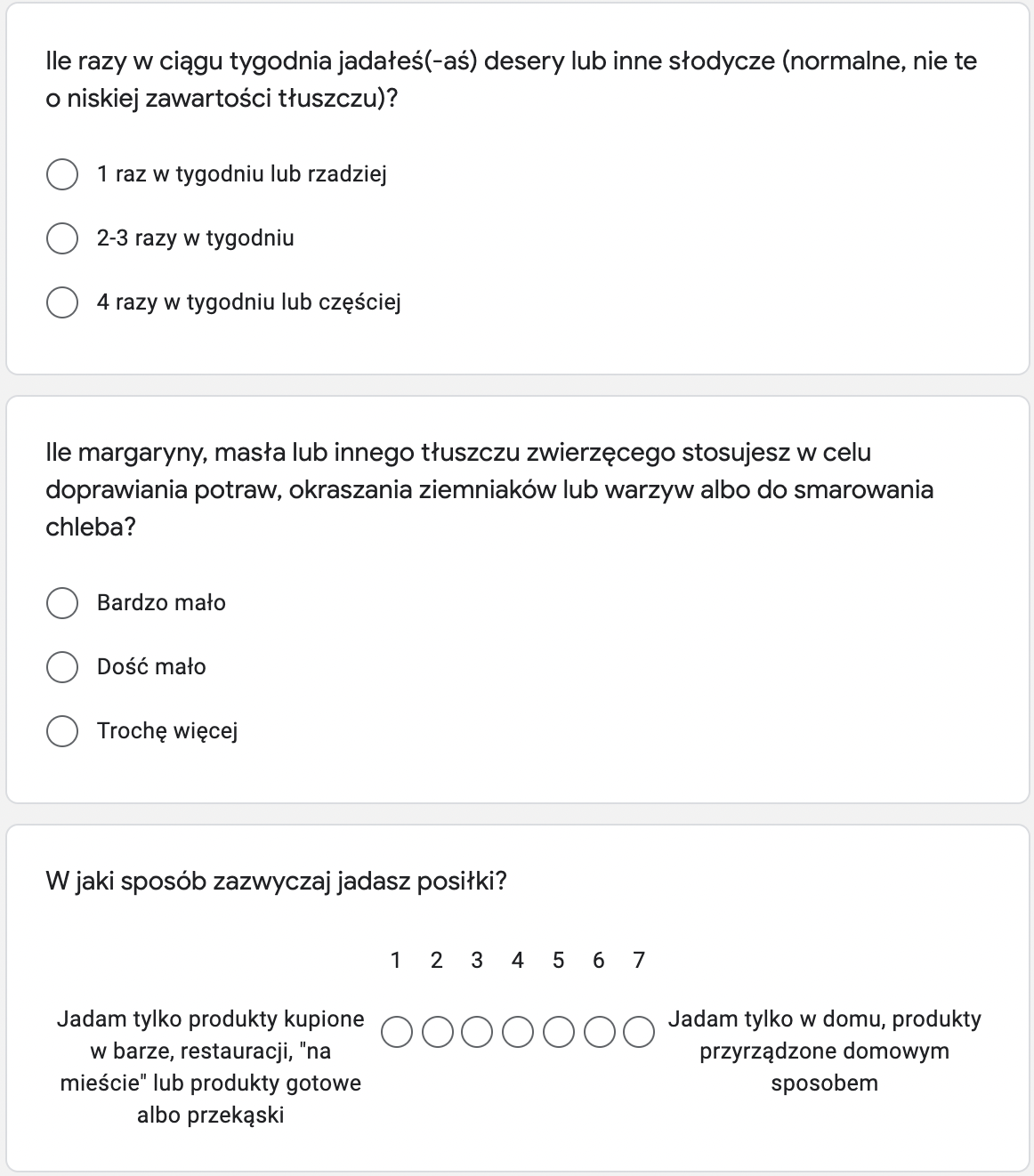
**

**
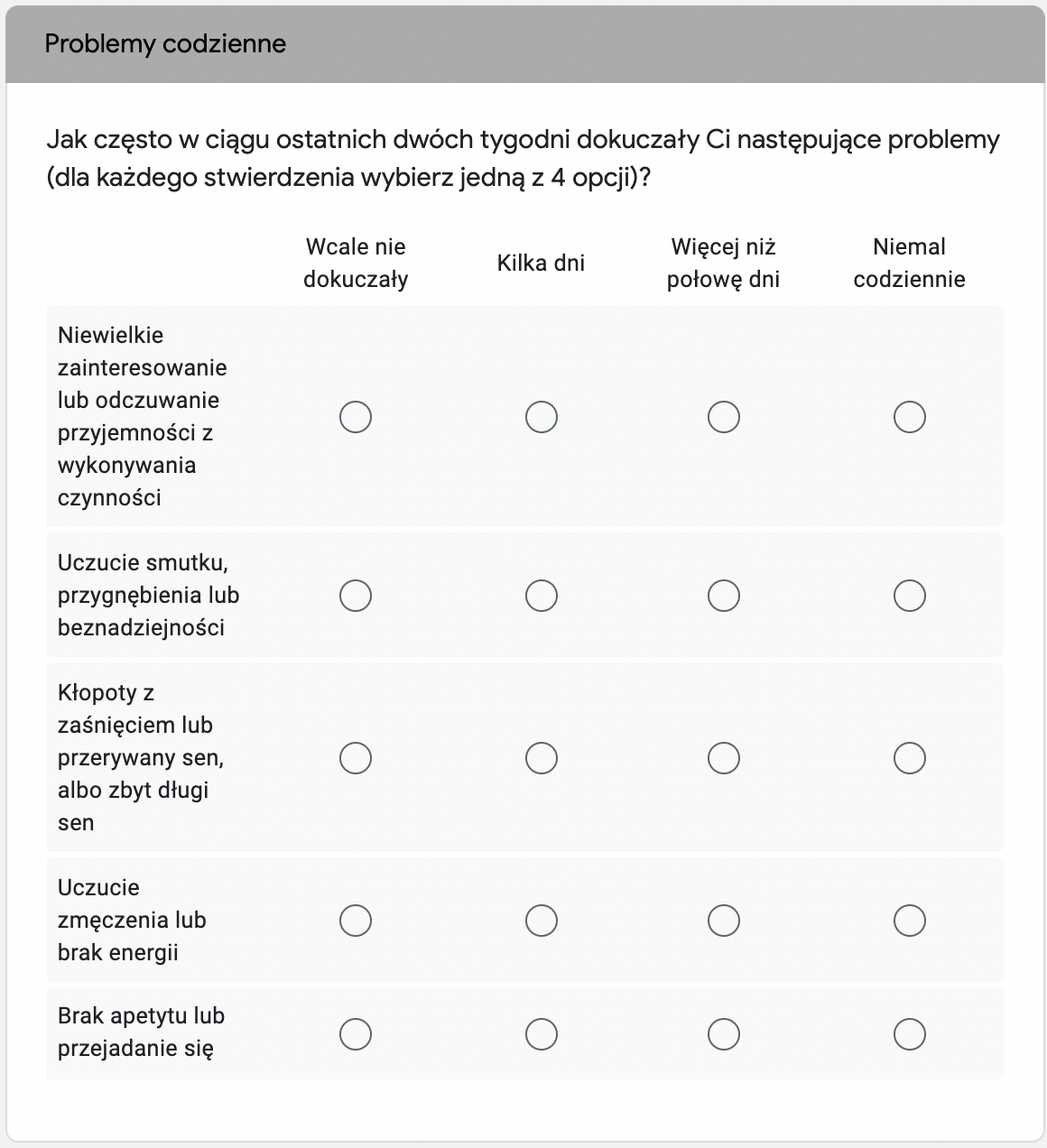
**

**
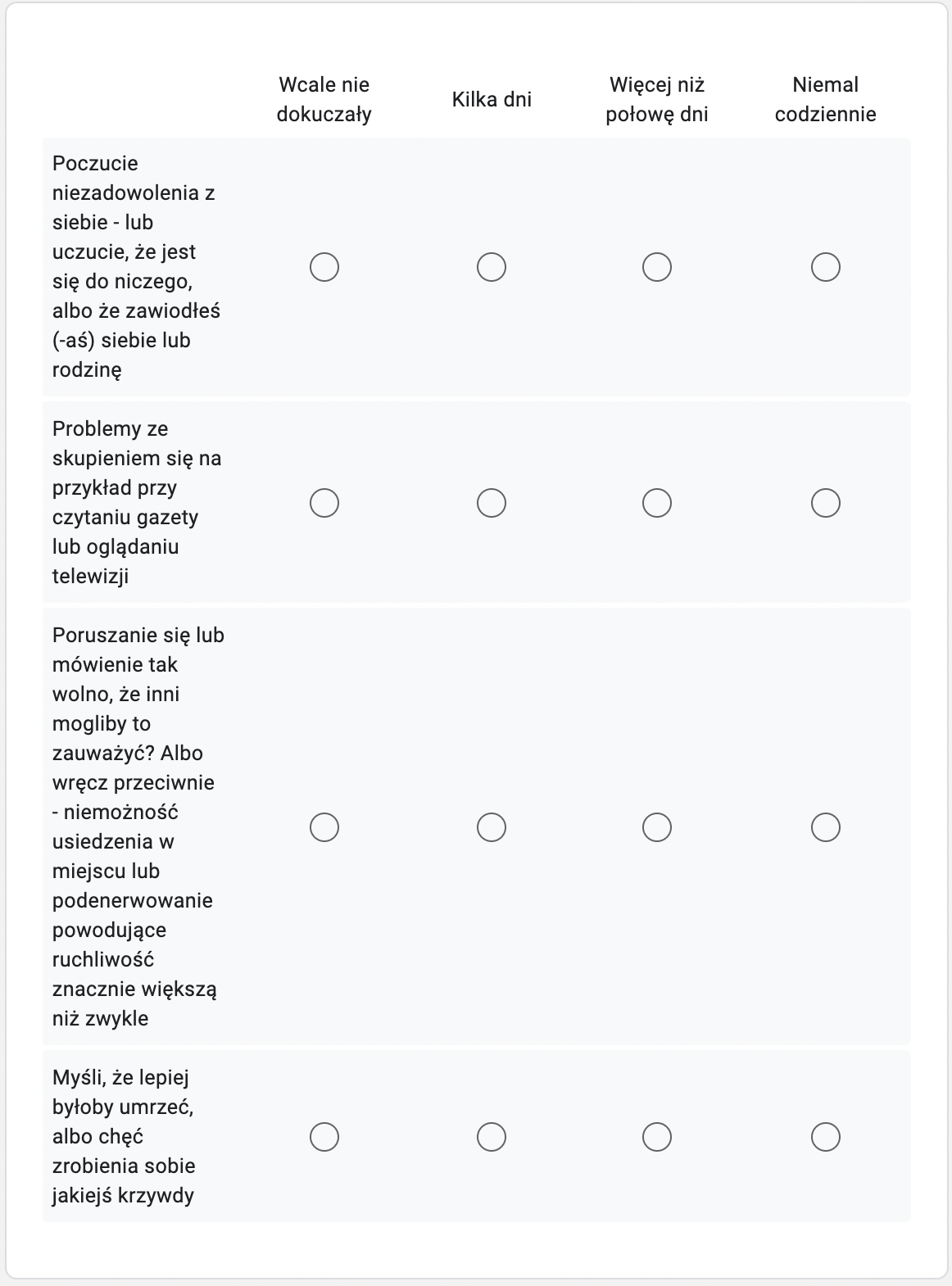
**

**
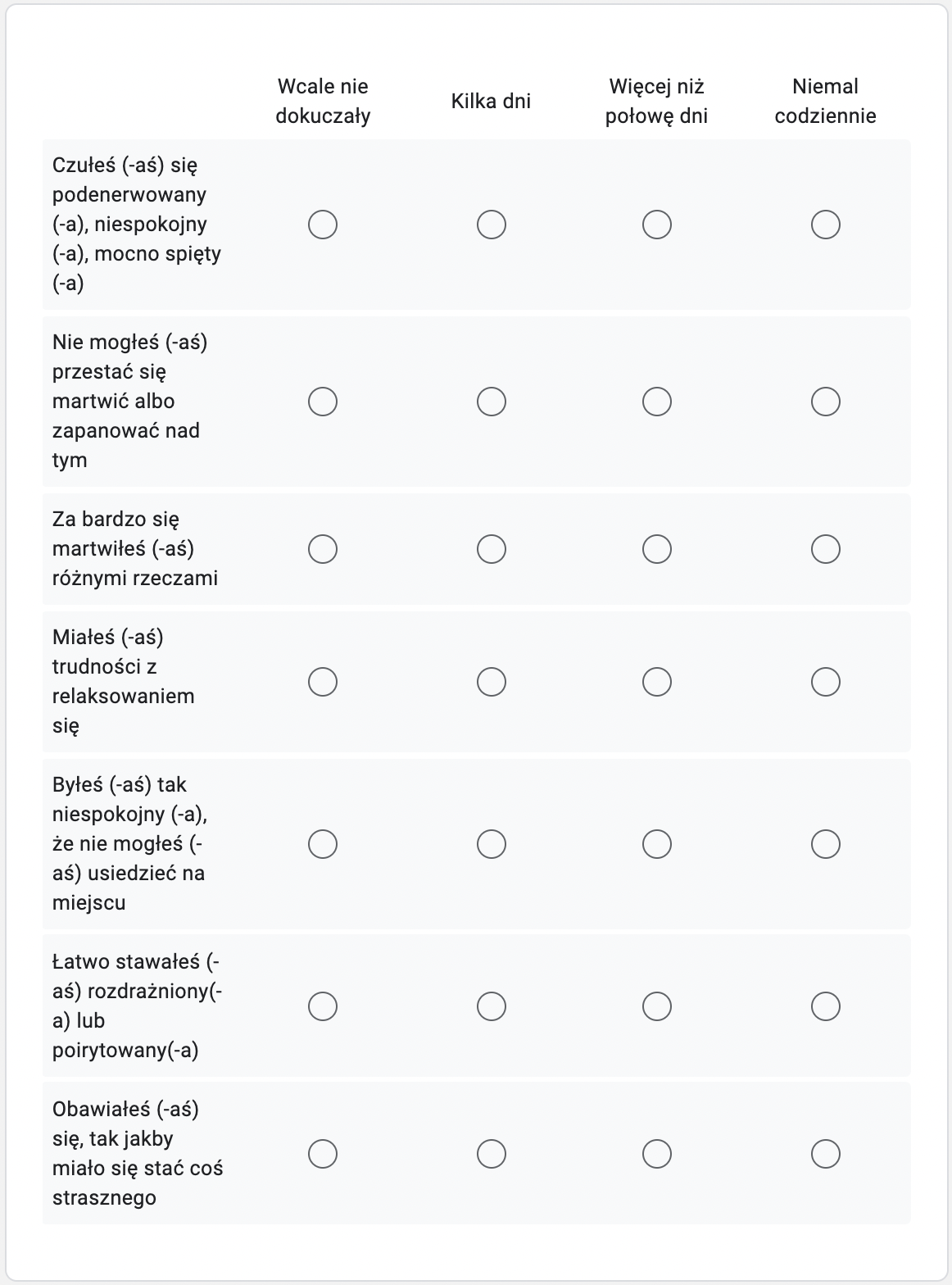
**

**
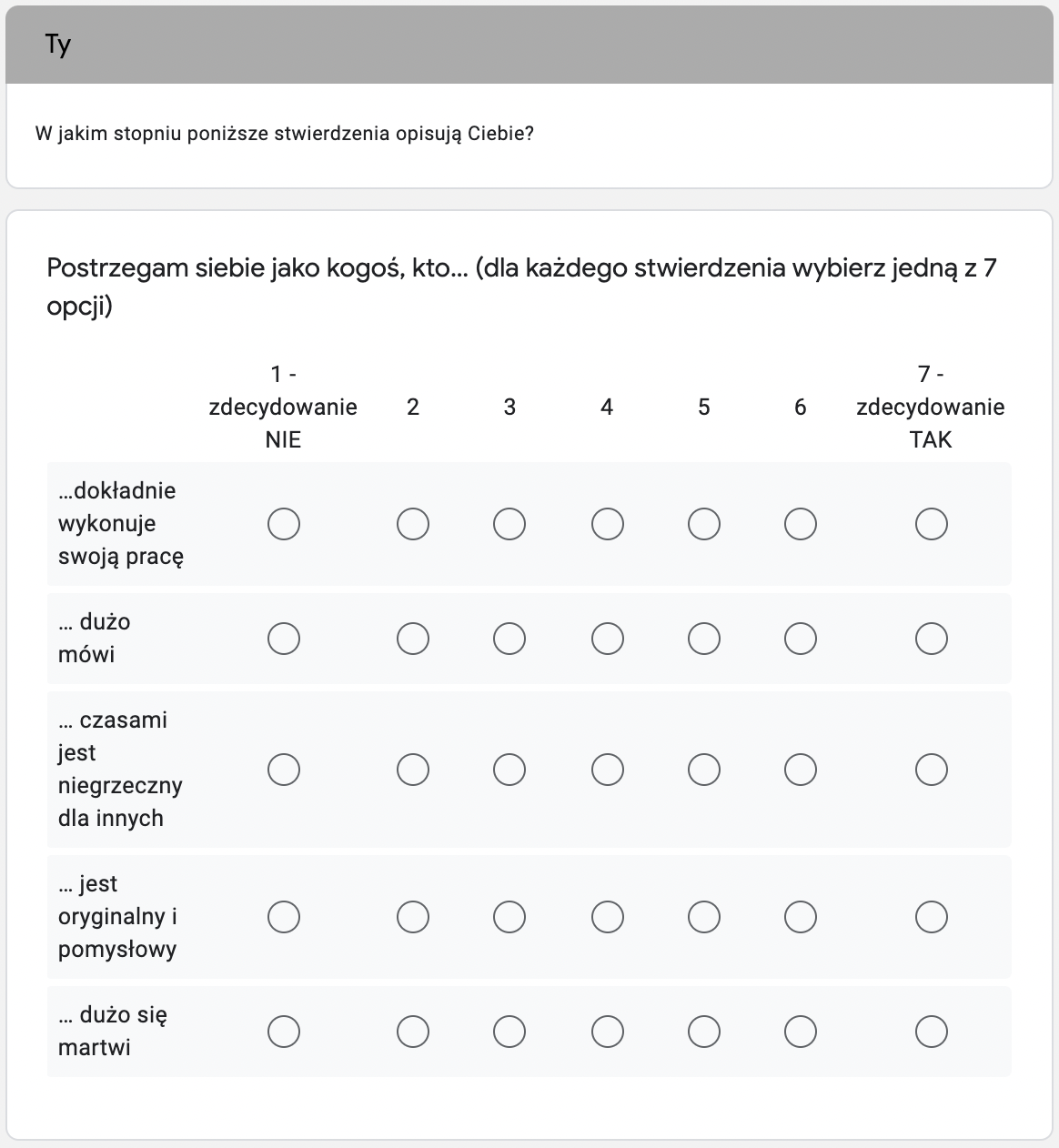
**

**
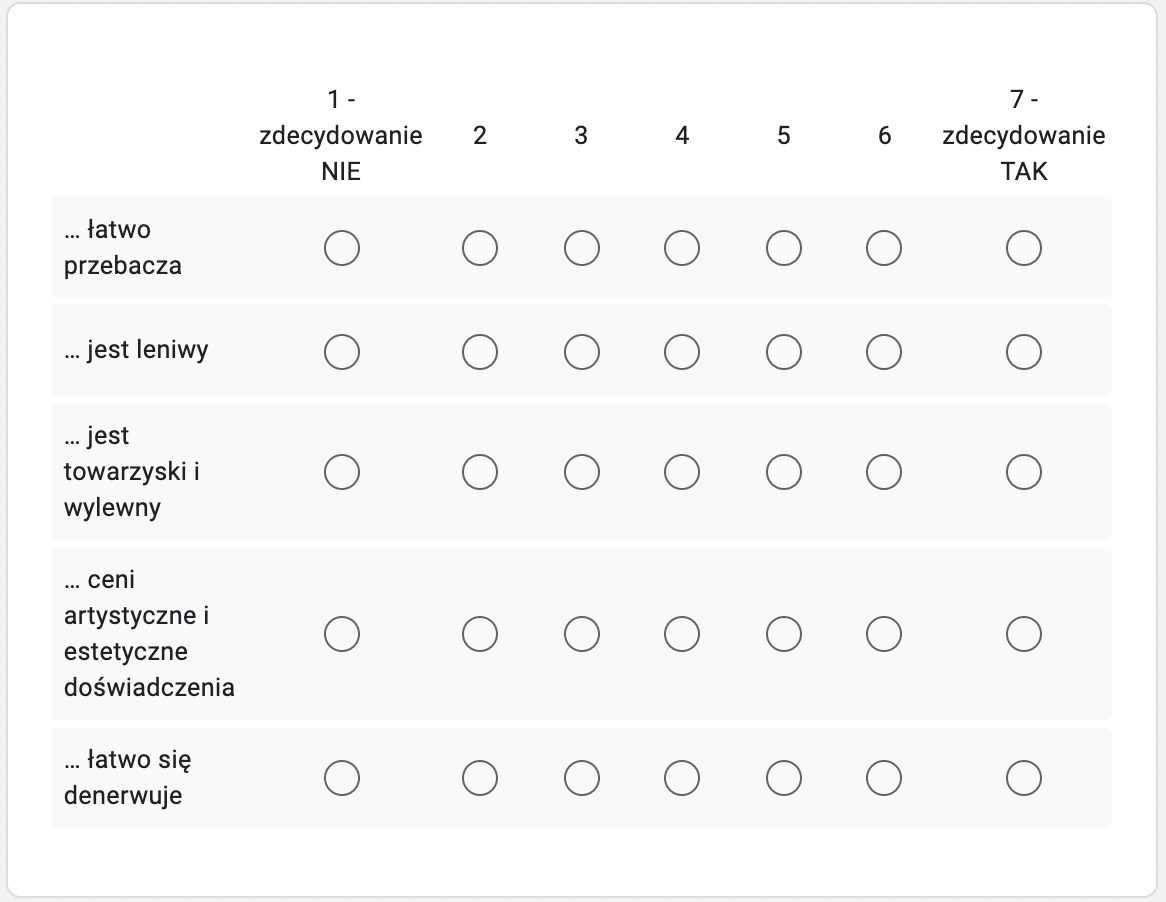
**

**
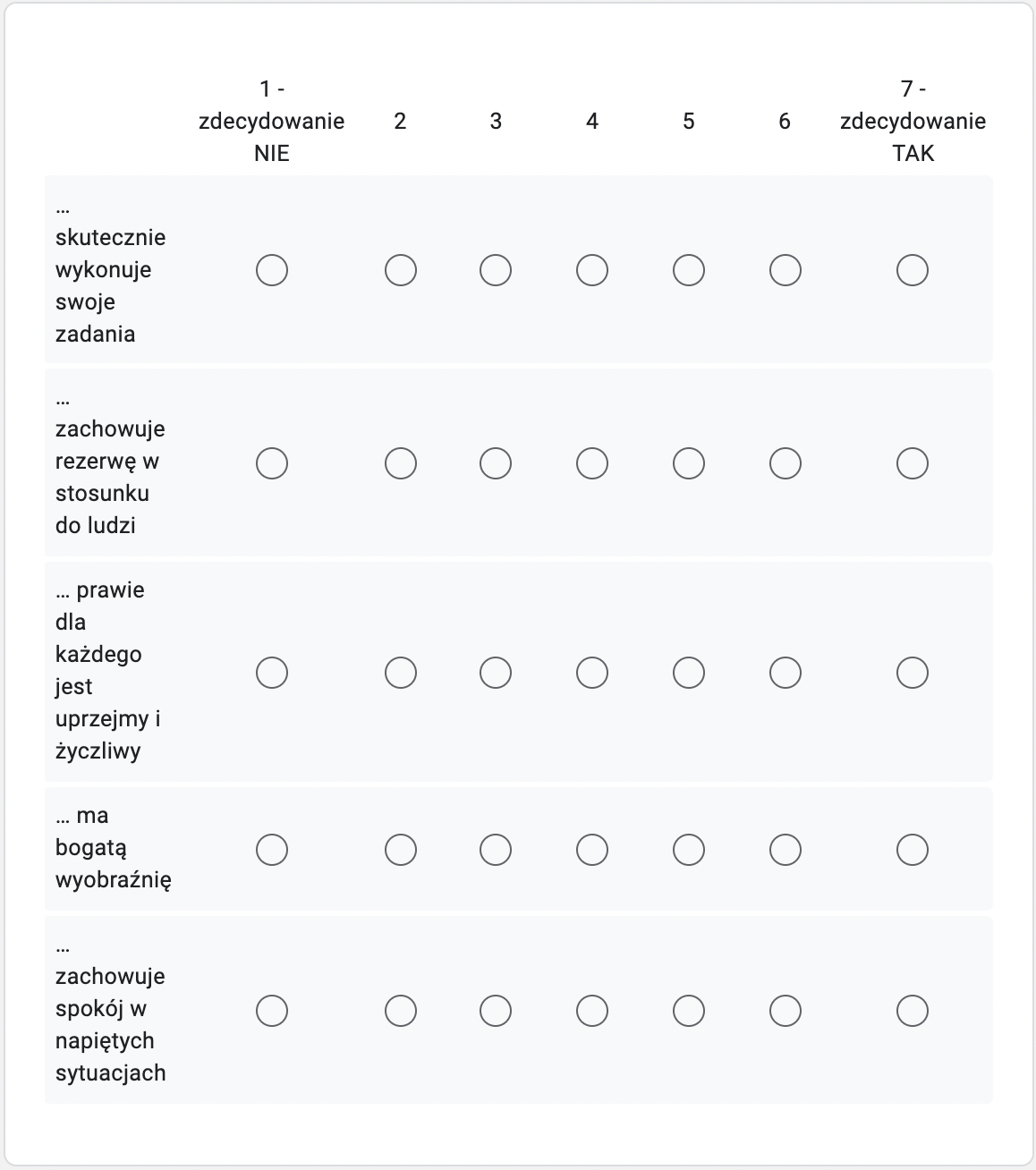
**

**
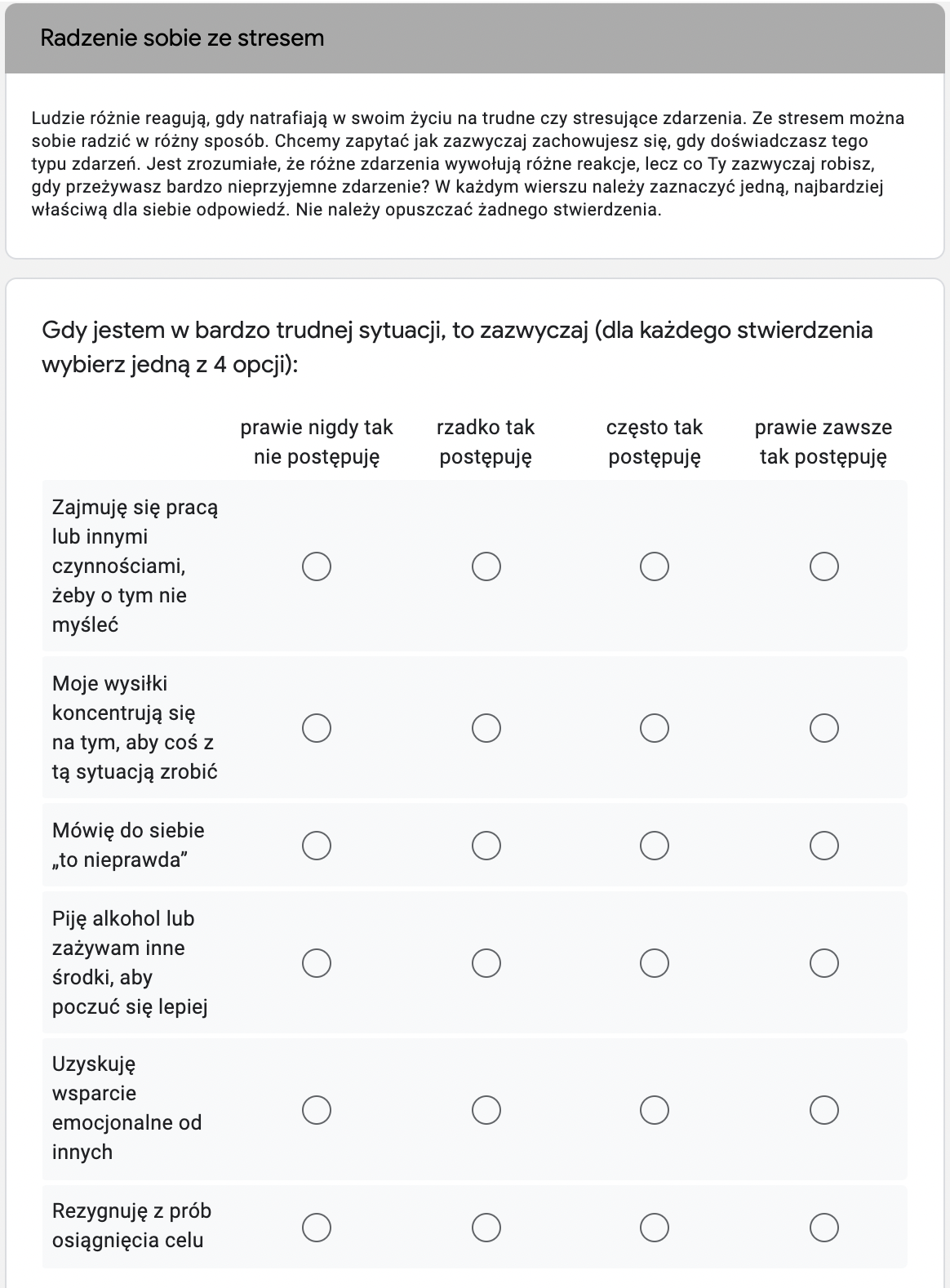
**

**
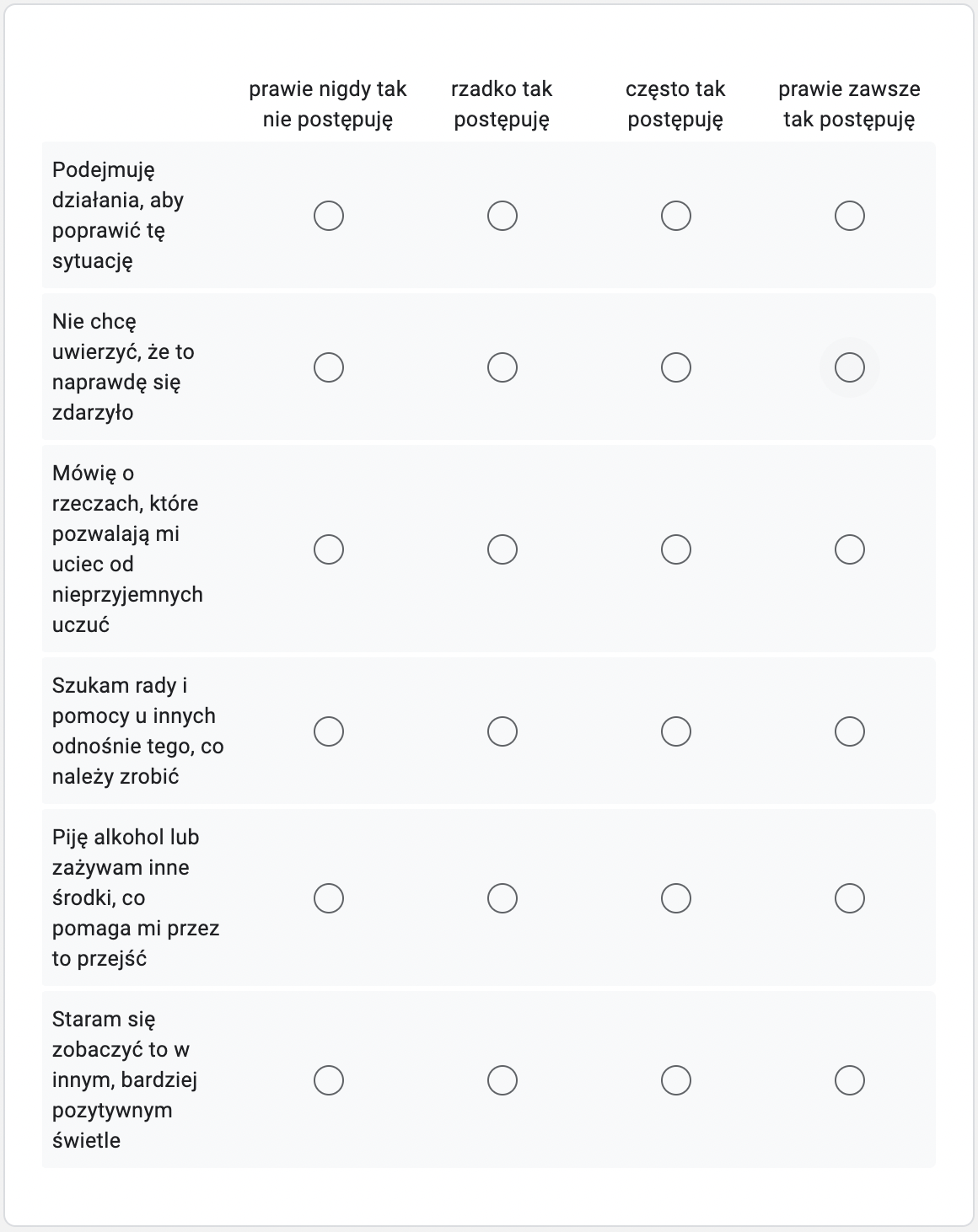
**

**
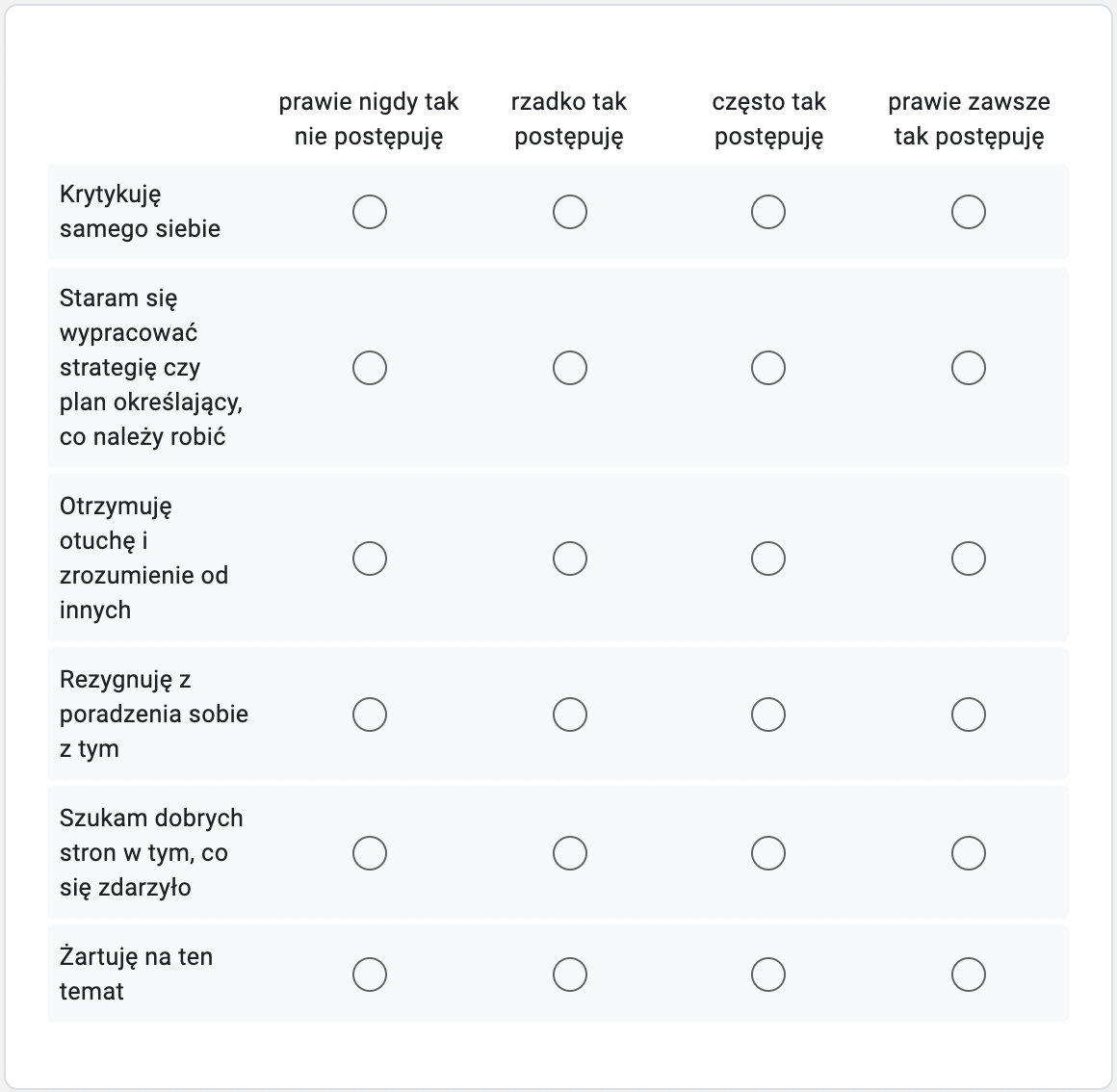
**

**
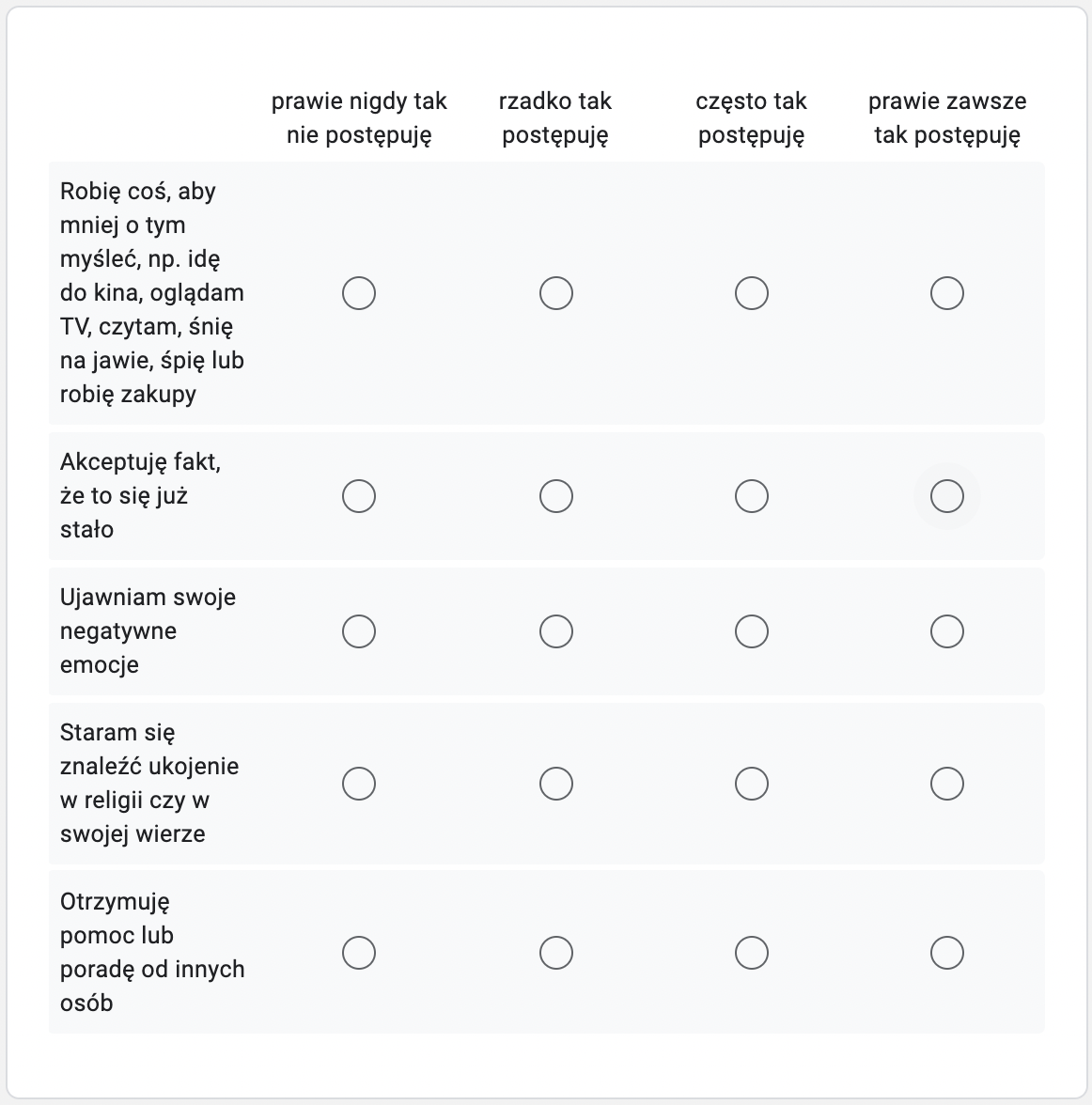
**

**
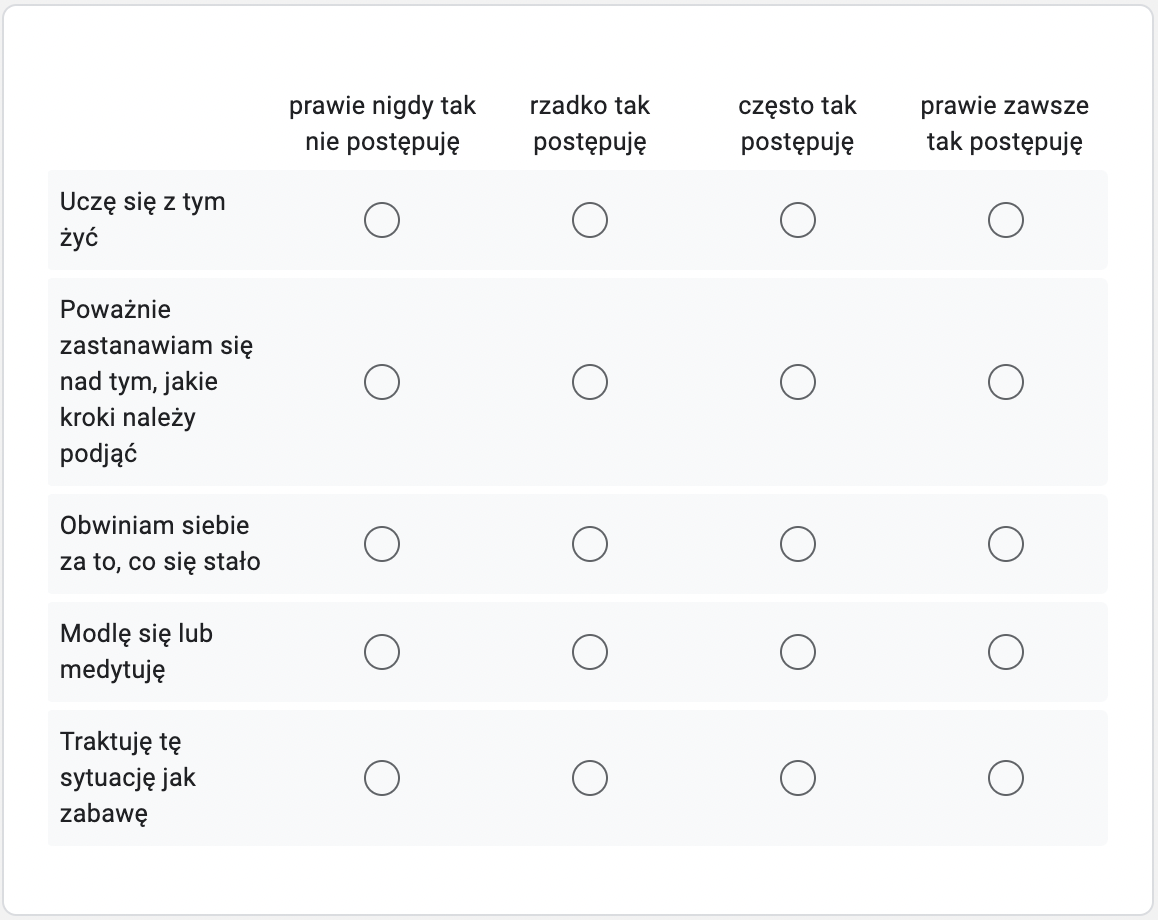
**

**
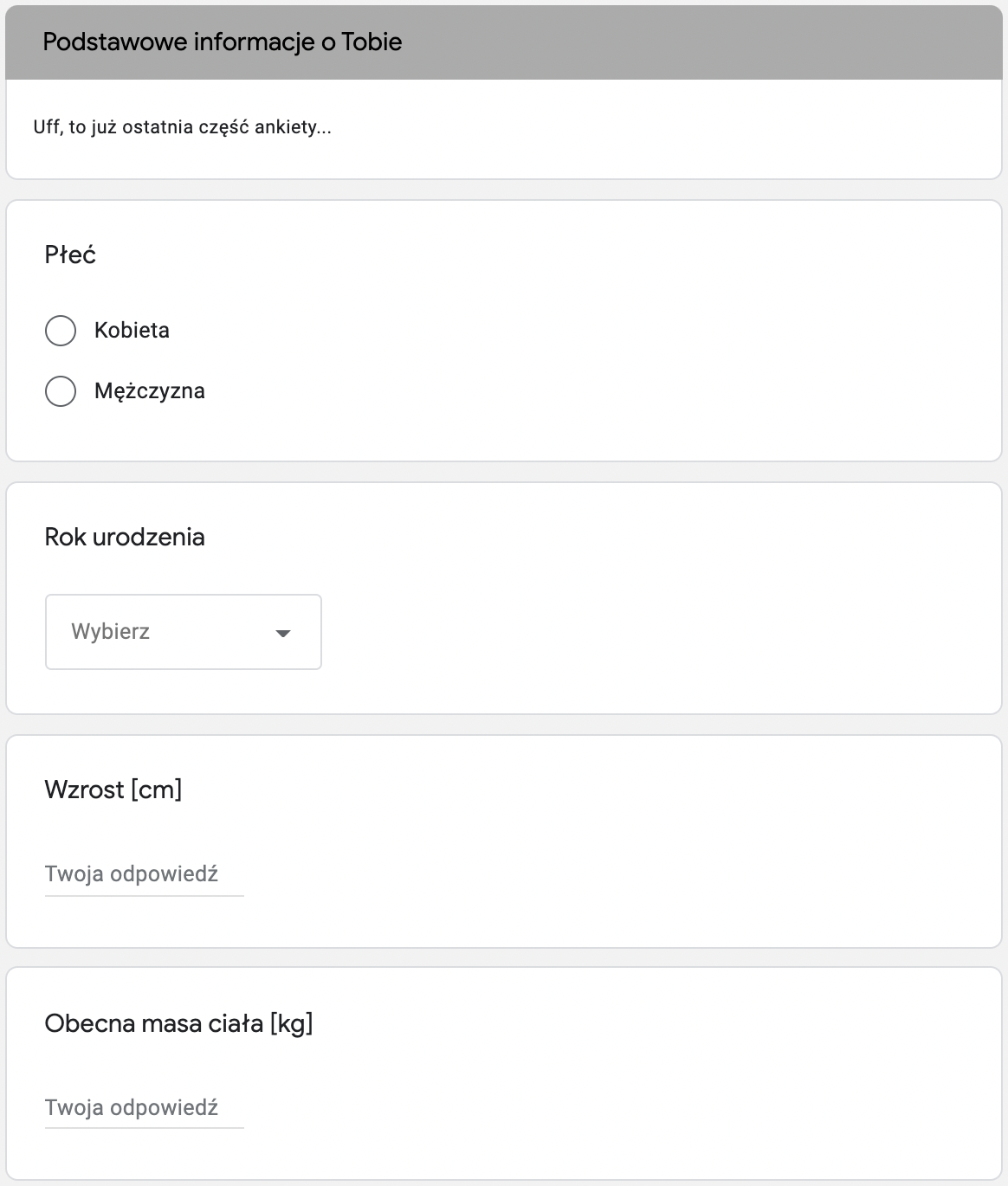
**

**
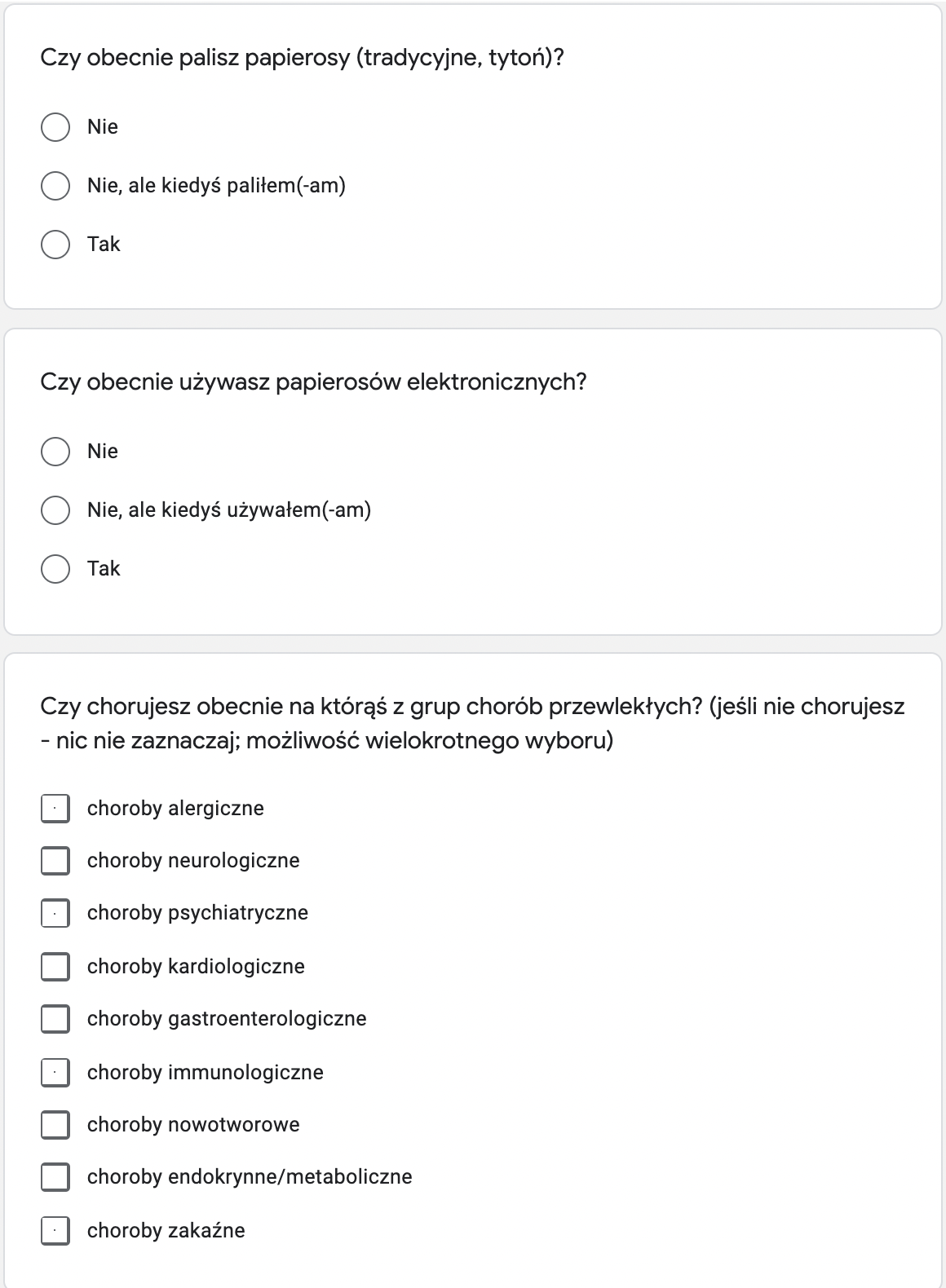
**

**
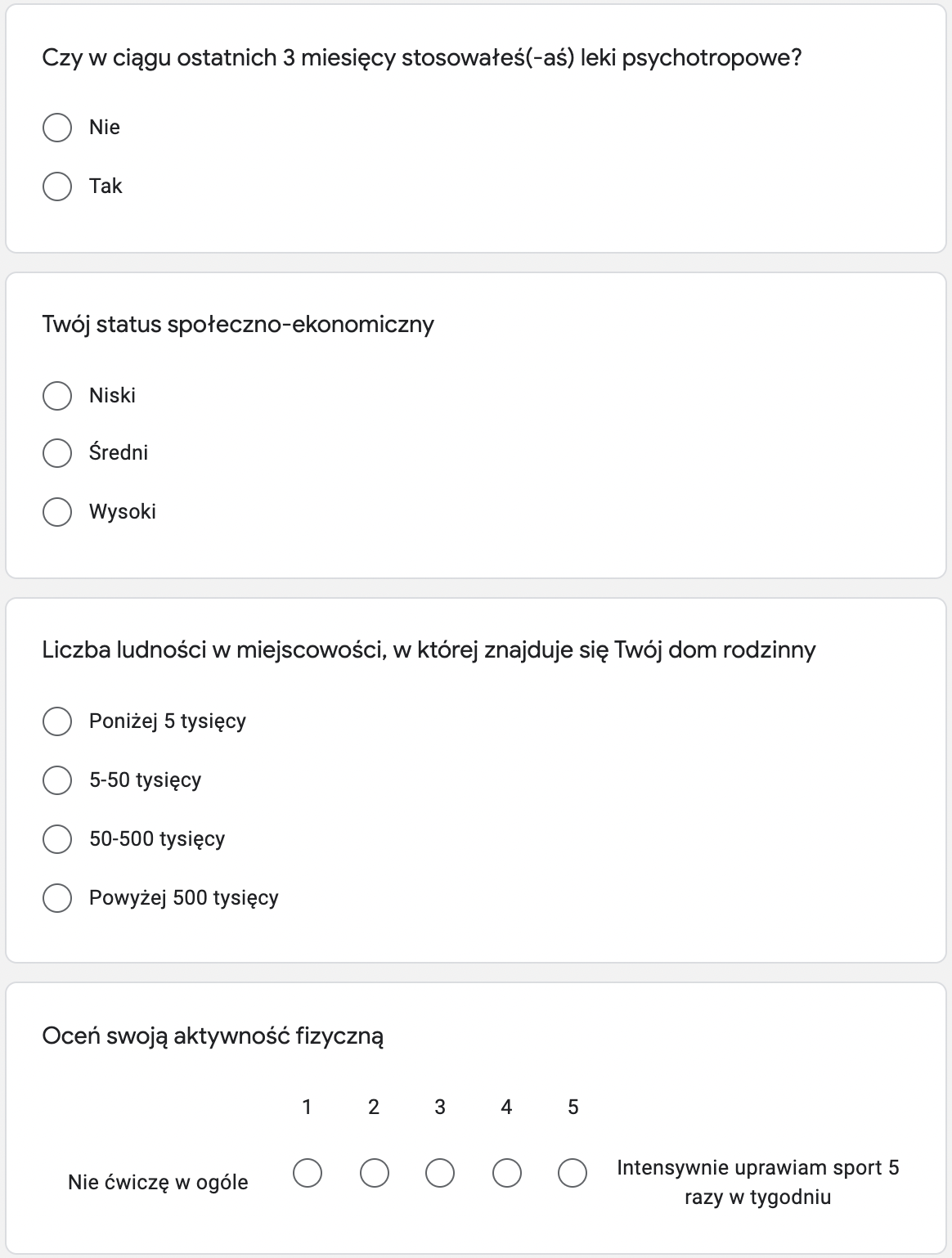
**

**
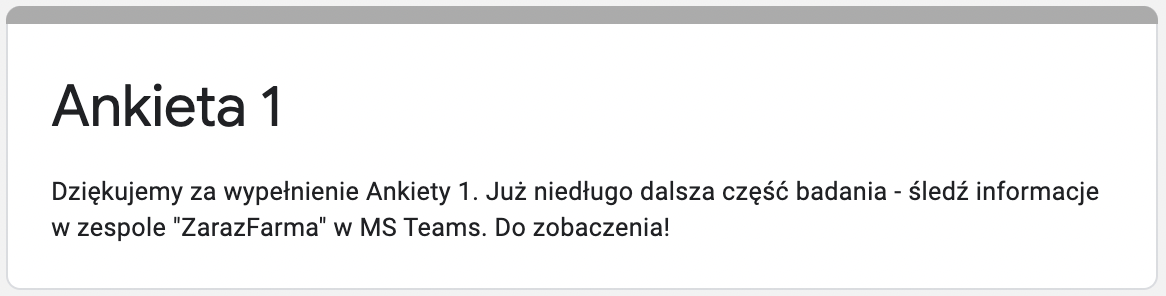
**

**English language translation of the original survey**

(The survey in the English language version is the free translations of the authors, it has not been validated for research use.)

**Survey 1**

Dear Students,

We kindly invite you to participate in the scientific research: “Association between lifestyle factors and cognitive performance under stress” (ZarazFarma). In the presented survey we will ask you about your lifestyle, especially diet, everyday problems and your ways of coping with stress; we will also collect basic demographic data about you.

Participation in the research is voluntary. There is a possibility to resign at any time without giving reasons or suffering any consequences. When it comes to your personal data, we will ask only for your student ID number, which allows us to assign your survey results to your exam result. Other personal data which are included in your student record book (such as name and surname) will not be assigned to the results of any of the surveys in this research. The research was approved by the Bioethics Commission of the Medical University of Lodz (nr RNN/111/20/KE from the 2nd of April 2020). In the case of any questions related to the research, do not hesitate to contact dr. Michał Karbownik (by private message on MS Teams or by email: michal.karbownik@umed.lodz.pl) .

Filling out the survey will take around ten minutes. Please feel free to start when you’re ready!

**Informed and voluntary consent**

If you want to participate in the research, express it by marking the following statements:

- I have read the information for participants, which is available here: <http://tinyurl.com/ZarazFarma-informacja>.
- The researchers have provided full answers for my questions, and I am accurately informed about the course of the research.
- I meet the inclusion criteria to the research: I am a third year medical student from the Medical University of Lodz and I do not have any impediments to take the final Pharmacology exam.
- I give my informed and voluntary consent to participate in the study “Association between lifestyle factors and cognitive performance under stress” (ZarazFarma); this will be required to fill out two online surveys and to participate in a seven-day-long diet recording. I know that I can resign from the participation at any time. I accept the terms of insurance in the case of harm related to the participation in the research. I give consent for my data to be processed in compliance with the Data Protection Act.

**Number of student record book**

Please, write your student ID number.

**Diet**

Over the past few months:

How many times a week did you eat fast food meals or snacks?

- Less than 1
- 1-2
- 3 or more

How many servings of fruit did you eat each day?

- 1 or less
- 2-3
- 4 or more

How many servings of vegetables did you eat each day?

- 2 or less
- 3-4
- 5 or more

How many glasses of carbonated drinks (such as cola, orangeade, energy drink) or glasses of sweet tea did you drink each day?

- less than 1
- around 1
- more than 1

How many times a week did you eat leguminous vegetables, chicken or fish?

- once or less
- 2-3
- 5 or more

How many times a week did you eat regular snack chips or crackers (not the low-fat kind)?

- once or less
- 2-3
- 4 or more

How much margarine, butter, or meat fat did you use to season vegetables or put on potatoes or bread?

- very little
- some
- a lot

What kind of meals have you generally eaten?

| I have eaten only street food or food bought in bars, restaurants or highly-processed food and snacks. | 1----2----3----4----5----6----7 | I have eaten only products cooked on my own at home. |
| --- | --- | --- |

**Everyday problems**

Over the last two weeks, how often have you been bothered by the following problems?

(Please, for each statement choose one out of four options.)

|  | Not at all | Several days | More than half the days | Nearly every day |
| --- | --- | --- | --- | --- |
| Little interest or pleasure in doing things |  |  |  |  |
| Feeling down, depressed,  or hopeless |  |  |  |  |
| Trouble falling or staying asleep, or sleeping too much |  |  |  |  |
| Feeling tired or having little energy |  |  |  |  |
| Poor appetite or overeating |  |  |  |  |
| Feeling bad about yourself -  or that you are a failure or have let yourself or your family down |  |  |  |  |
| Trouble concentrating on things, such as reading the newspaper  or watching television |  |  |  |  |
| Moving or speaking so slowly that other people could have noticed?  Or the opposite - being so fidgety or restless that you have been moving around a lot more than usual |  |  |  |  |
| Thoughts that you would be better off dead, or of hurting yourself  in some way |  |  |  |  |
| Feeling nervous, anxious,  or on edge |  |  |  |  |
| Not being able to stop or control worrying |  |  |  |  |
| Worrying too much about different things |  |  |  |  |
| Trouble relaxing |  |  |  |  |
| Being so restless that it is hard to sit still |  |  |  |  |
| Becoming easily annoyed  or irritable |  |  |  |  |
| Feeling afraid, as if something awful might happen |  |  |  |  |

**Yourself**

How much do these statements describe you? (Please, for each statement choose one out of seven options.)

| I See Myself as Someone Who... | 1  Definitely no | 2 | 3 | 4 | 5 | 6 | 7  Definitely yes |
| --- | --- | --- | --- | --- | --- | --- | --- |
| Does a thorough job |  |  |  |  |  |  |  |
| Is talkative |  |  |  |  |  |  |  |
| Is sometimes rude to others |  |  |  |  |  |  |  |
| Is original, comes up with new ideas |  |  |  |  |  |  |  |
| Worries a lot |  |  |  |  |  |  |  |
| Has a forgiving nature |  |  |  |  |  |  |  |
| Tends to be lazy |  |  |  |  |  |  |  |
| Is outgoing, sociable |  |  |  |  |  |  |  |
| Values artistic, aesthetic experiences |  |  |  |  |  |  |  |
| Gets nervous easily |  |  |  |  |  |  |  |
| Does things efficiently |  |  |  |  |  |  |  |
| Is reserved |  |  |  |  |  |  |  |
| Is considerate and kind to almost everyone |  |  |  |  |  |  |  |
| Has an active imagination |  |  |  |  |  |  |  |
| Remains calm in tense situations |  |  |  |  |  |  |  |

**Coping with stress**

People react differently when difficult or stressful events appear. There are many different methods of coping with stress. We would like to ask you how you usually behave when you experience these kinds of events. It is understandable that different events cause different reactions, but what do you usually do when you experience a very unpleasant event? Please, choose one, the most accurate option for each statement. Do not miss any statement.

In a very difficult situation, I…

|  | I haven't been doing this at all | I've been doing this a little bit | I've been doing this a medium amount | I've been doing this a lot |
| --- | --- | --- | --- | --- |
| I've been turning to work or other activities to take my mind off things. |  |  |  |  |
| I've been concentrating my efforts on doing something about the situation I'm in. |  |  |  |  |
| I've been saying to myself "this isn't real.". |  |  |  |  |
| I've been using alcohol or other drugs to make myself feel better. |  |  |  |  |
| I've been getting emotional support from others. |  |  |  |  |
| I've been giving up trying to deal with it. |  |  |  |  |
| I've been taking action to try to make the situation better. |  |  |  |  |
| I've been refusing to believe that it has happened. |  |  |  |  |
| I've been saying things to let my unpleasant feelings escape. |  |  |  |  |
| I’ve been getting help and advice from other people. |  |  |  |  |
| I've been using alcohol or other drugs to help me get through it. |  |  |  |  |
| I've been trying to see it in a different light, to make it seem more positive. |  |  |  |  |
| I’ve been criticizing myself. |  |  |  |  |
| I've been trying to come up with a strategy about what to do. |  |  |  |  |
| I've been getting comfort and understanding from someone. |  |  |  |  |
| I've been giving up the attempt to cope. |  |  |  |  |
| I've been looking for something good in what is happening. |  |  |  |  |
| I've been making jokes about it. |  |  |  |  |
| I've been doing something to think about it less, such as going to movies,  watching TV, reading, daydreaming, sleeping, or shopping. |  |  |  |  |
| I've been accepting the reality of the fact that it has happened. |  |  |  |  |
| I've been expressing my negative feelings. |  |  |  |  |
| I've been trying to find comfort in my religion or spiritual beliefs. |  |  |  |  |
| I’ve been trying to get advice or help from other people about what to do. |  |  |  |  |
| I've been learning to live with it. |  |  |  |  |
| I've been thinking hard about what steps to take. |  |  |  |  |
| I’ve been blaming myself for things that happened. |  |  |  |  |
| I've been praying or meditating. |  |  |  |  |
| I've been making fun of the situation. |  |  |  |  |

**Basic information about You**

Uff, finally the last part of the survey…

Sex

- Female
- Male

Year of birth

Height [cm]

Current body mass [kg]

Do you smoke cigarettes or tobacco?

- No
- No, but I used to smoke
- Yes

Do you use electronic cigarettes?

- No
- No, but I used to use
- Yes

Do you suffer from any chronic disease? (If no, do not mark anything; multiple choice is available)

- allergic diseases
- neurological diseases
- psychiatric diseases
- cardiological diseases
- gastroenterological diseases
- immunological diseases
- neoplastic diseases
- endocrine or metabolic diseases
- infectious diseases

Have you used psychotropic drugs in the last three months?

- Yes
- No

Your socio-economic status

- Low
- Average
- High

The number of habitants in a locality where your family house is located

- Less than 5 thousand
- 5-50 thousand
- 50-100 thousand
- More than 500 thousand

Assess your physical activity

| I do not exercise at all. | 1-----2-----3-----4-----5 | I do sports intensively five times a week. |
| --- | --- | --- |

**The First Survey**

Thank you for filling out The First Survey. The further part of the research will come soon - follow the information in the channel “ZarazFarma” on the MS Teams. See you!
