# Supplementary material for: Association Between Consumption of Fermented Food and Food-Derived Prebiotics With Cognitive Performance, Depressive, and Anxiety Symptoms in Psychiatrically Healthy Medical Students Under Psychological Stress: A Prospective Cohort Study
Source: Front Nutr. 2022 Mar 3;9:850249. doi: 10.3389/fnut.2022.850249 (PMC8929173; doi:10.3389/fnut.2022.850249)
Supplement: Supplementary file 3 [file Data_Sheet_3.DOCX]

***Supplementary Material 3***

***Food Record form “My meal”***

The screenshots of the original form are presented below.


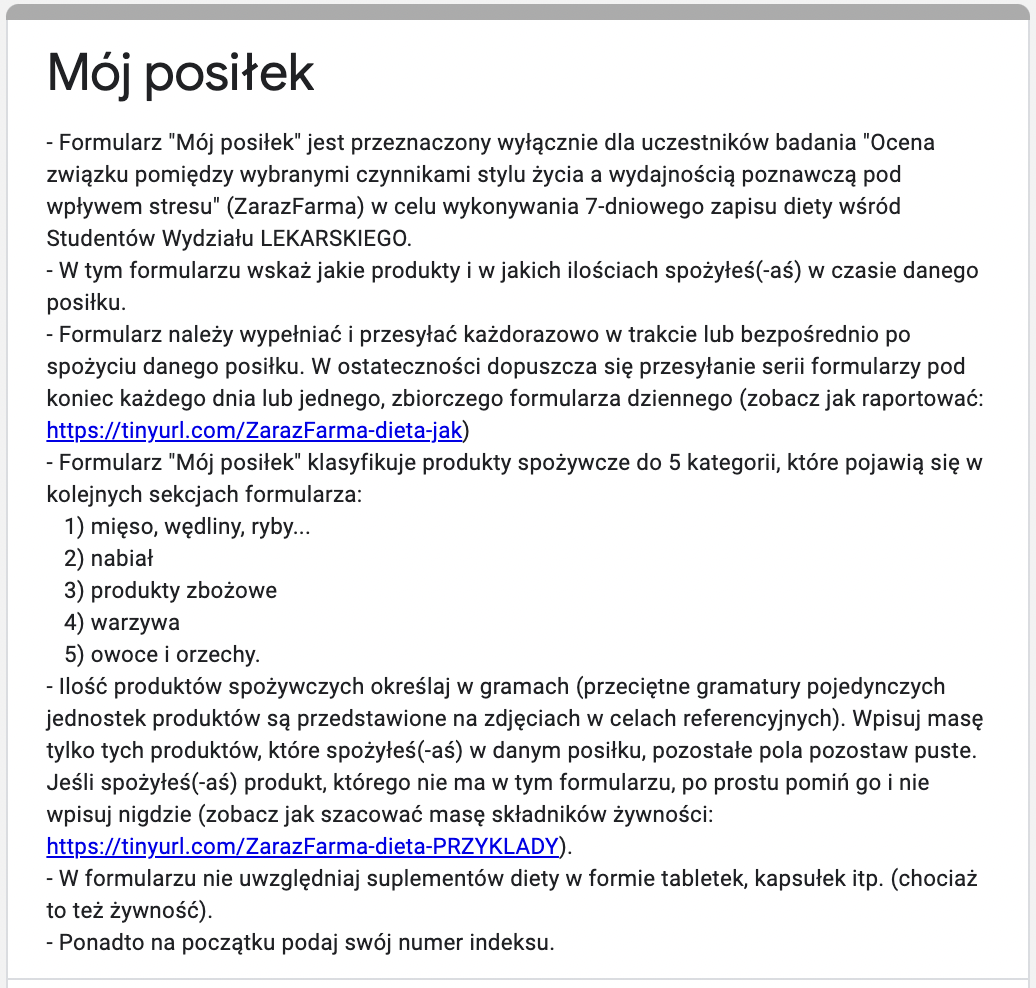


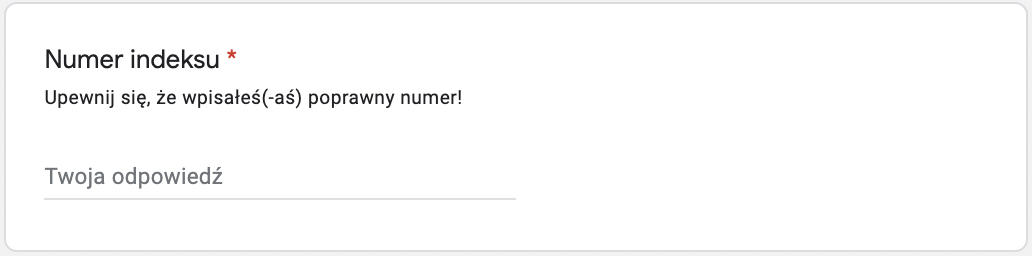


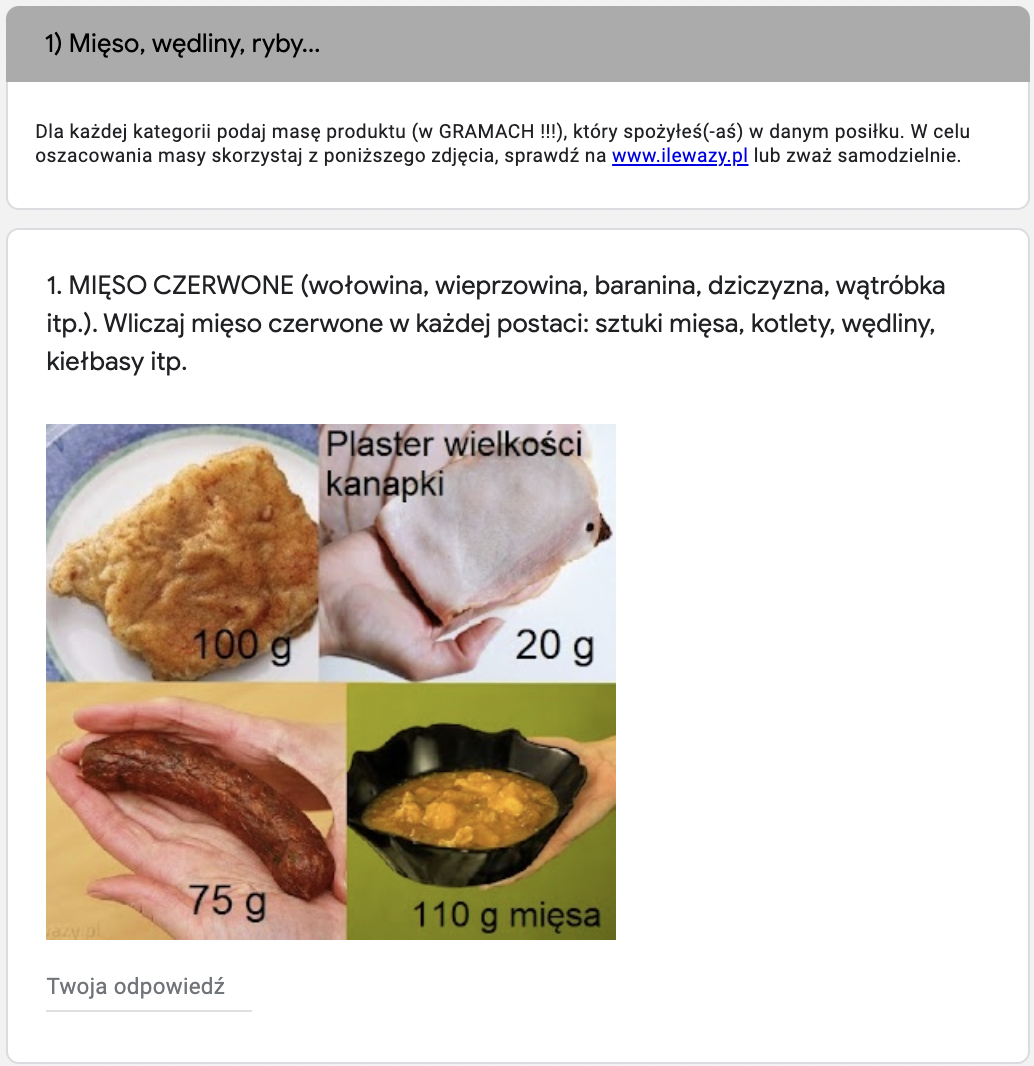


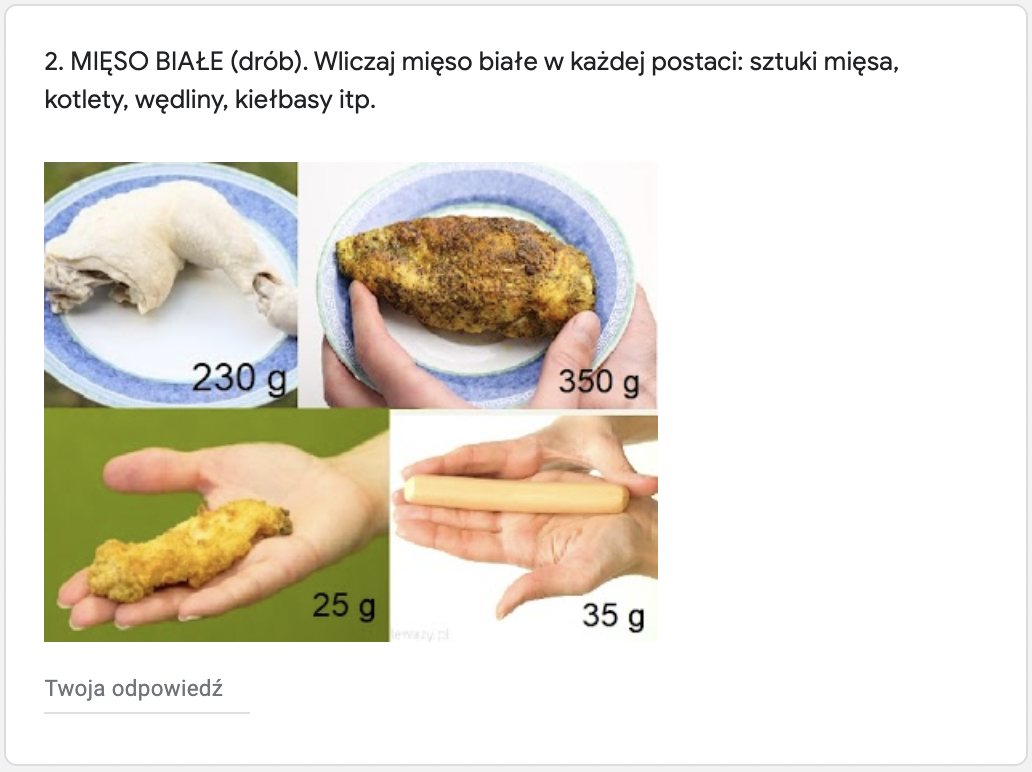


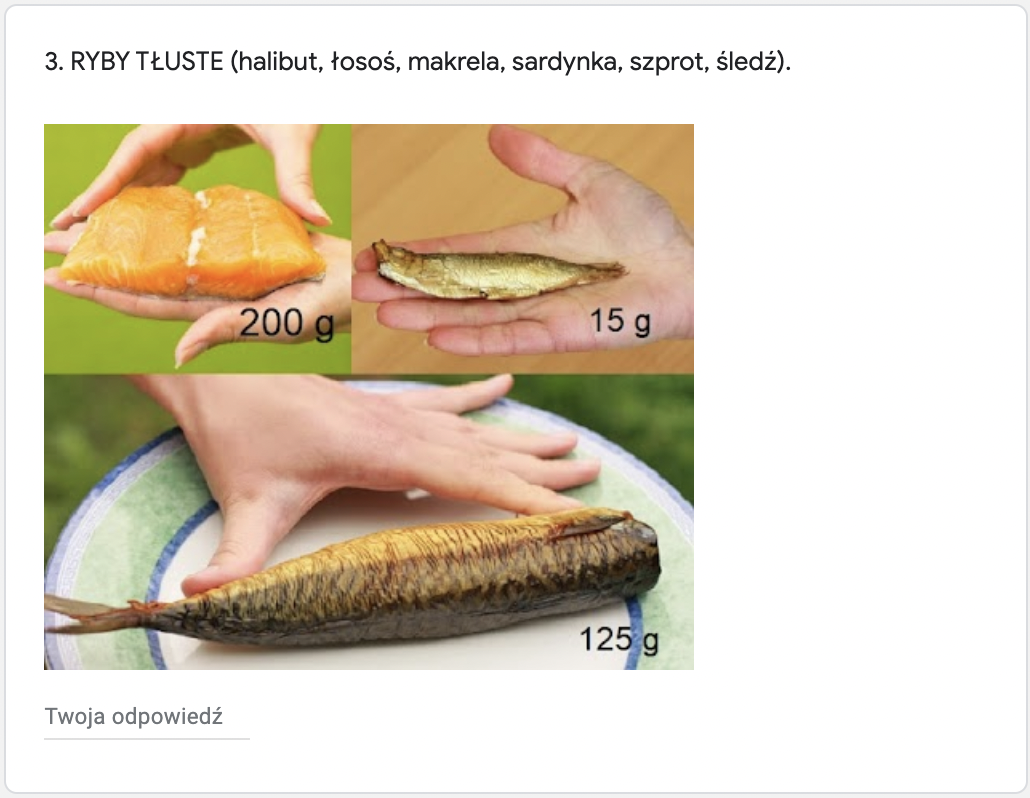


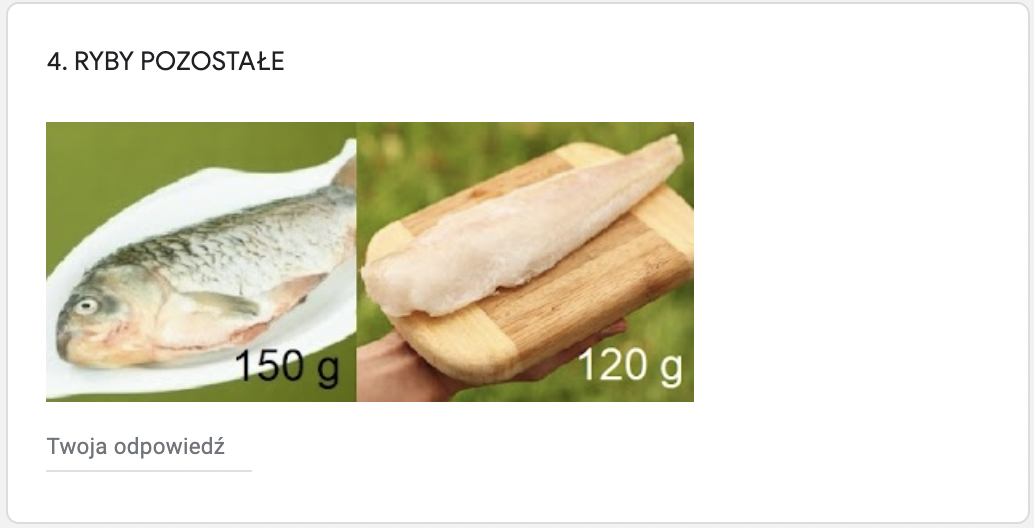


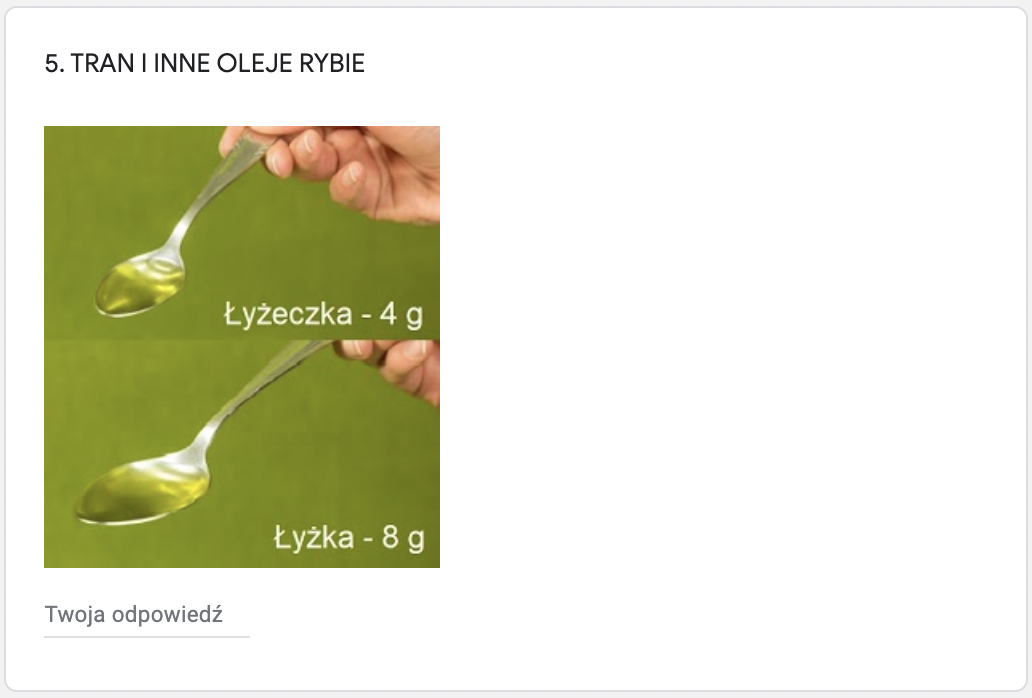


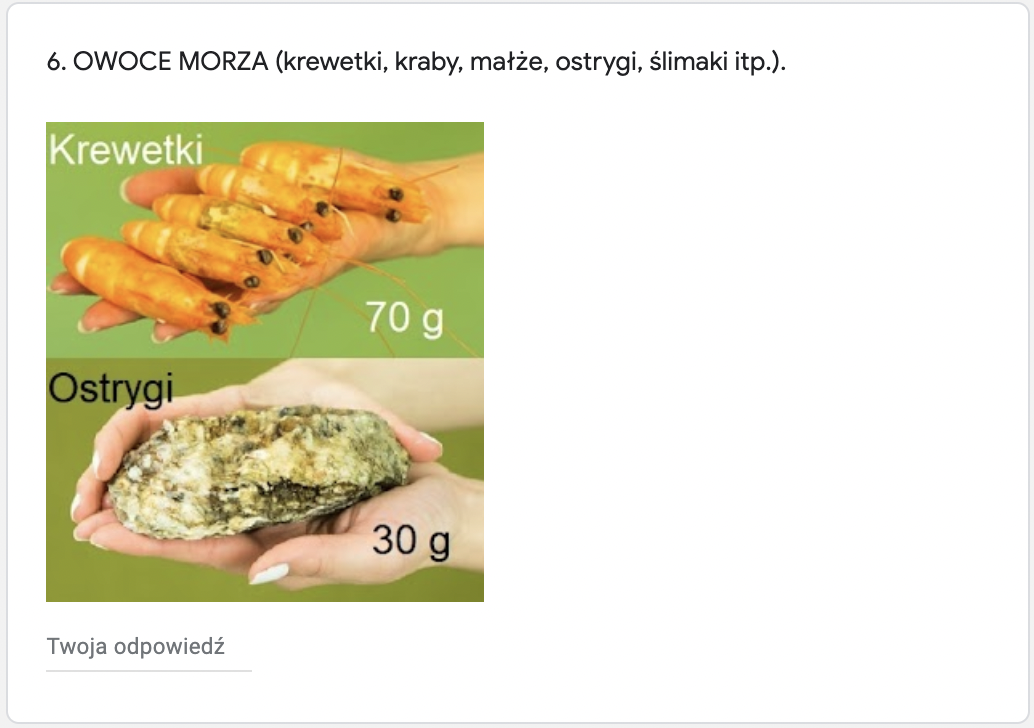


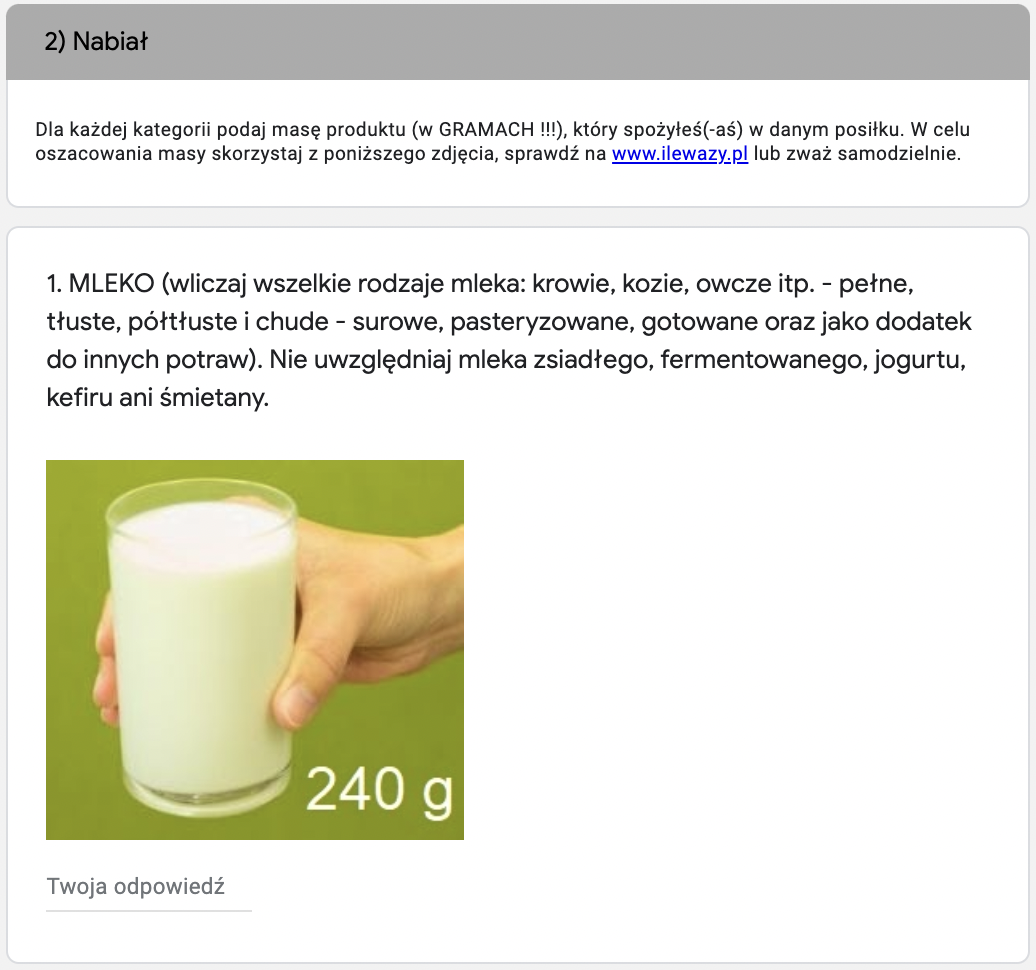


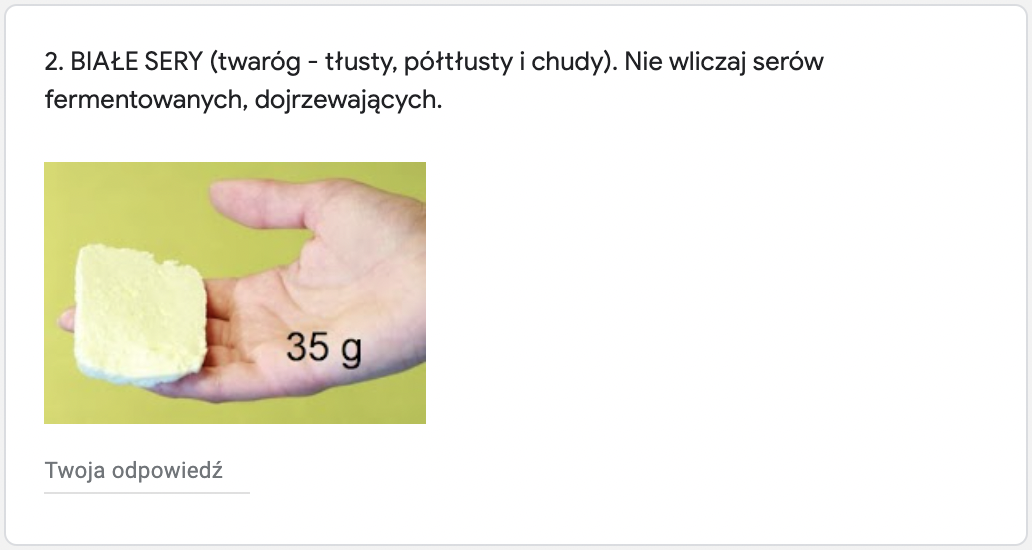


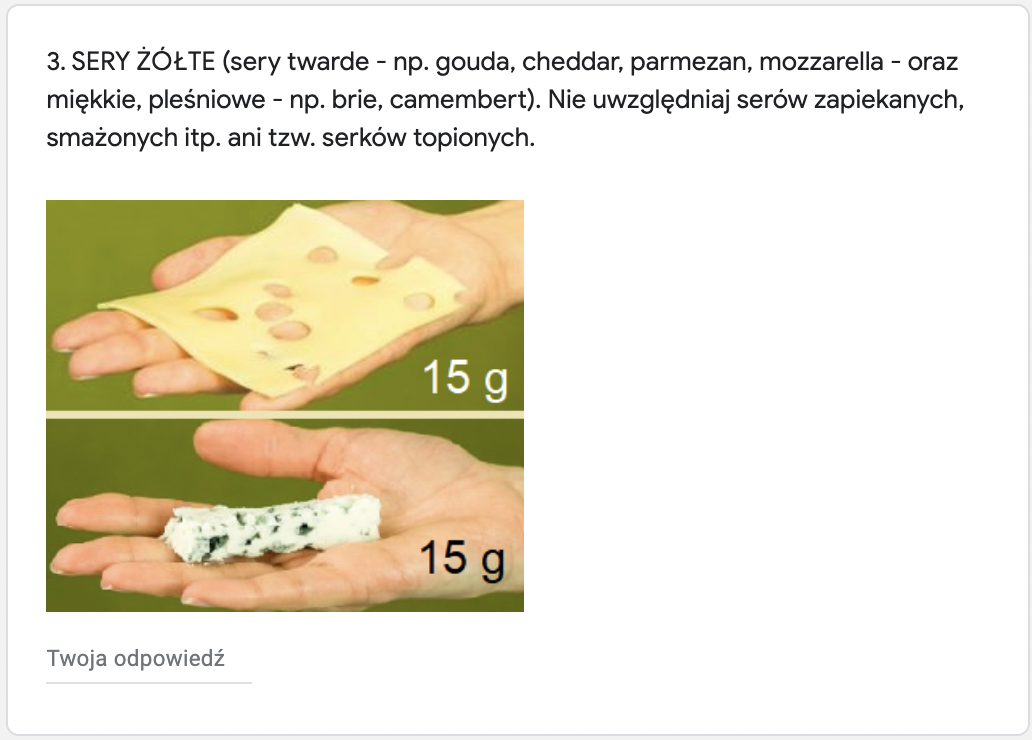


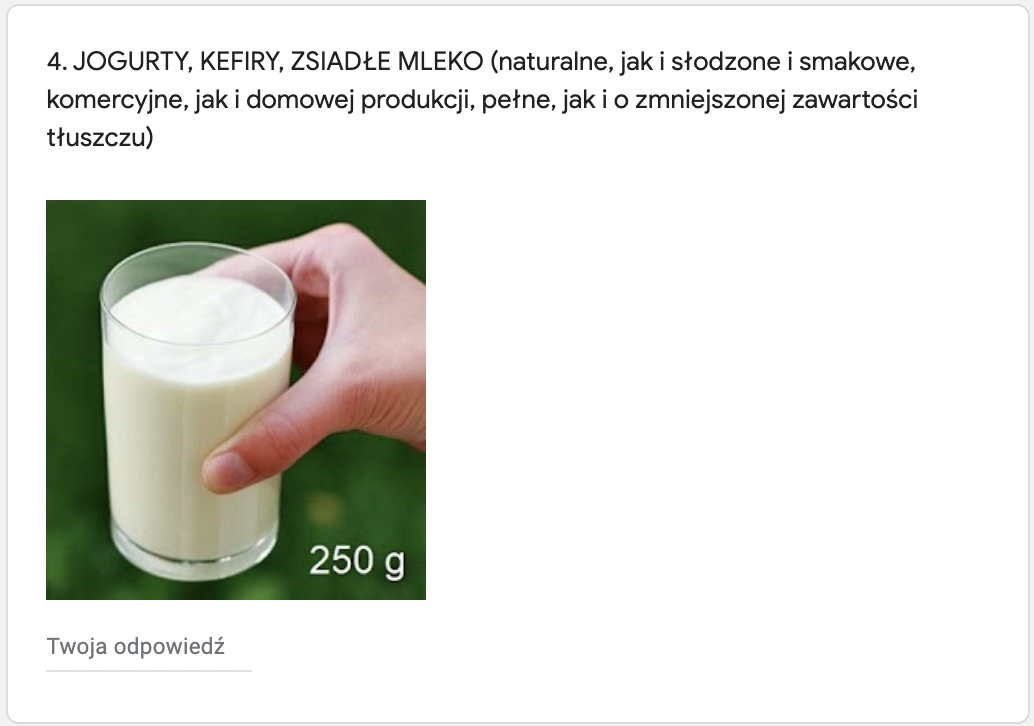


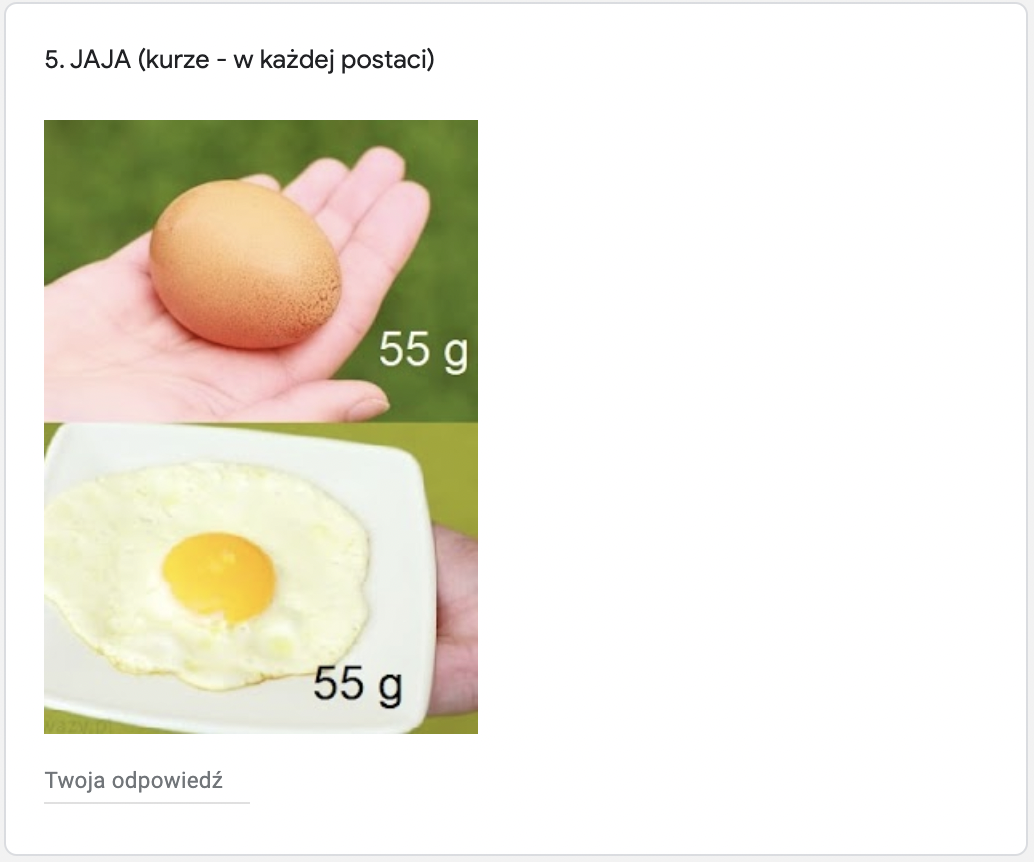

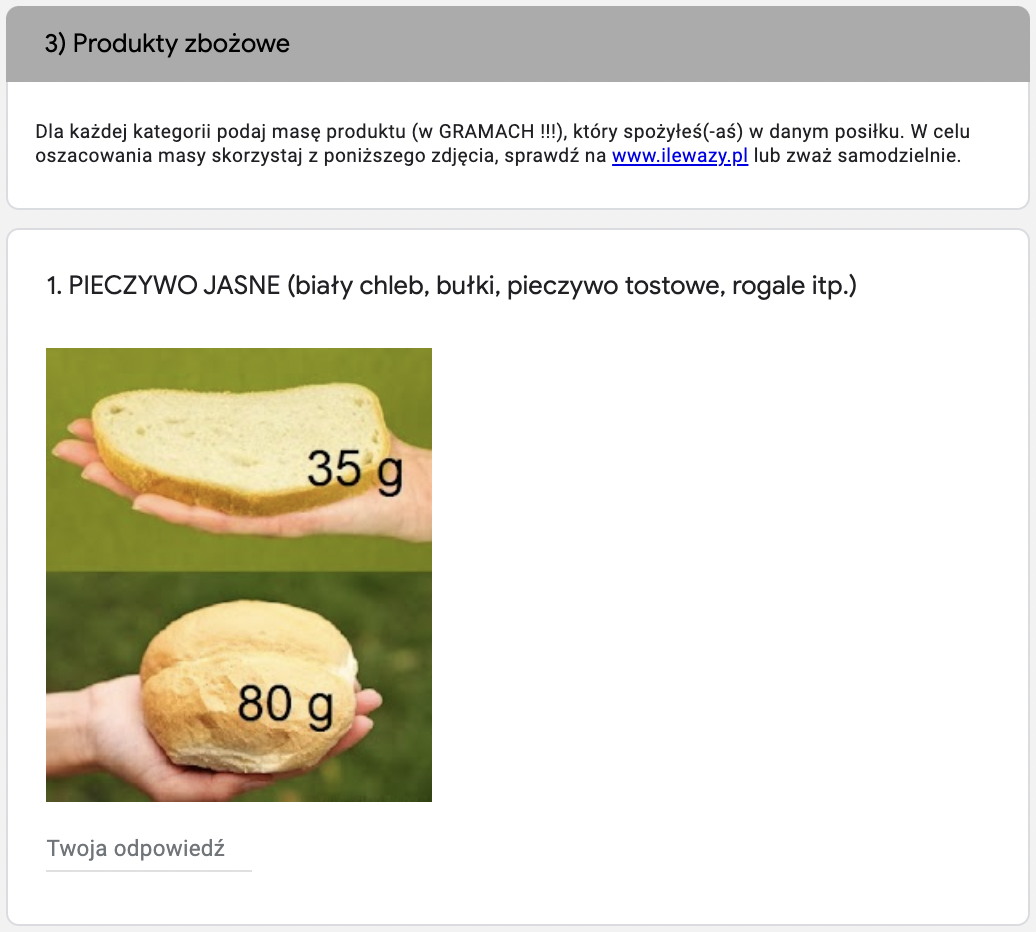


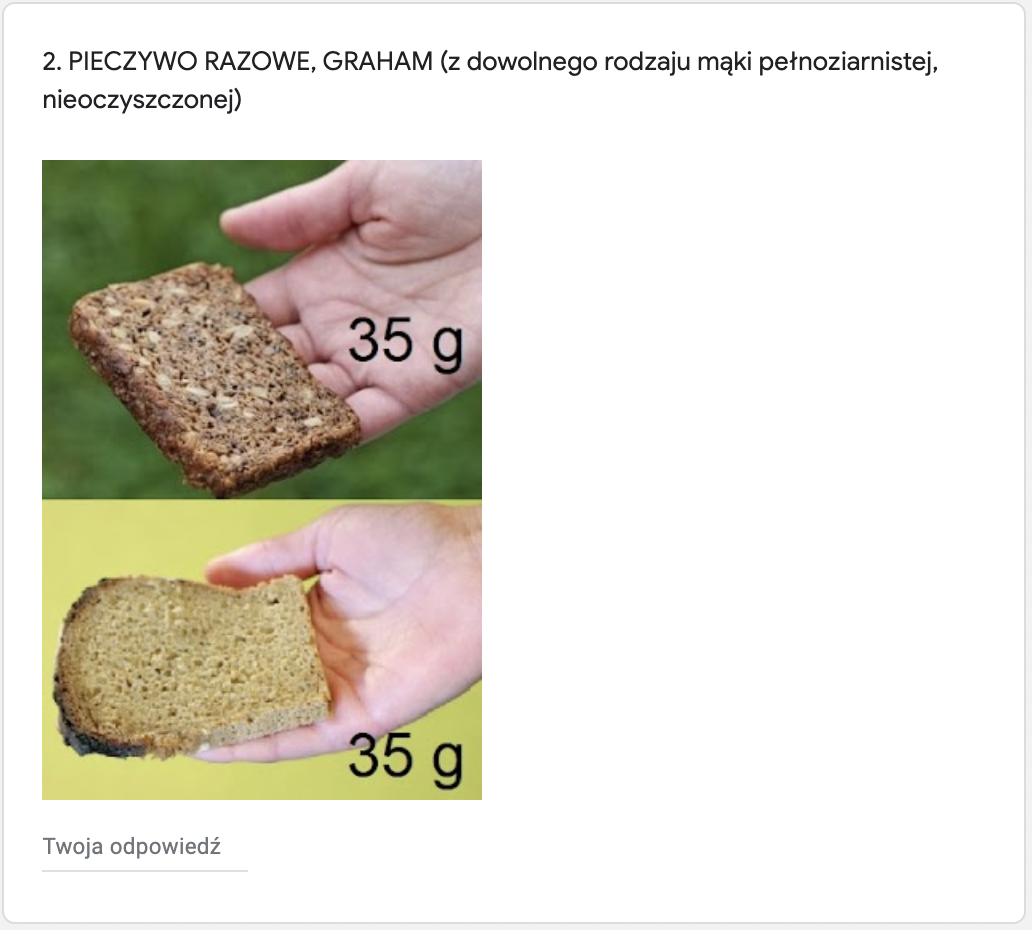


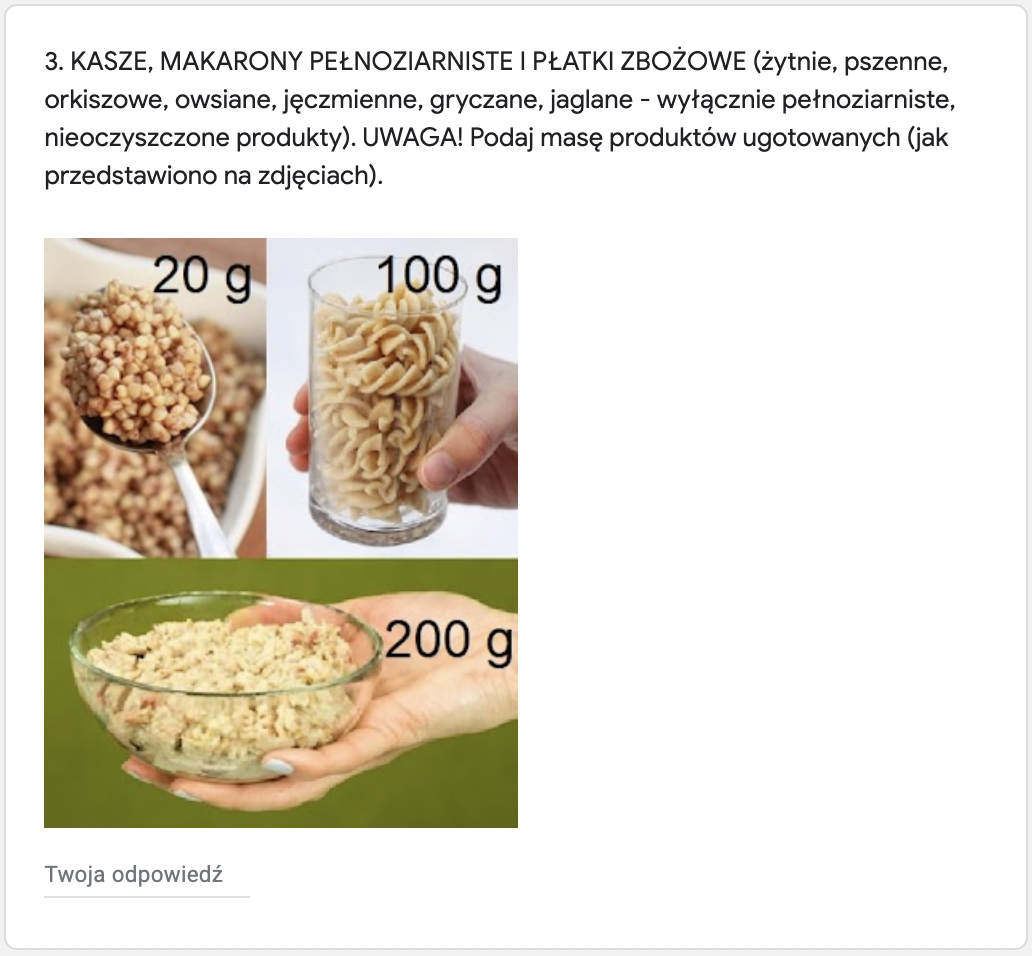


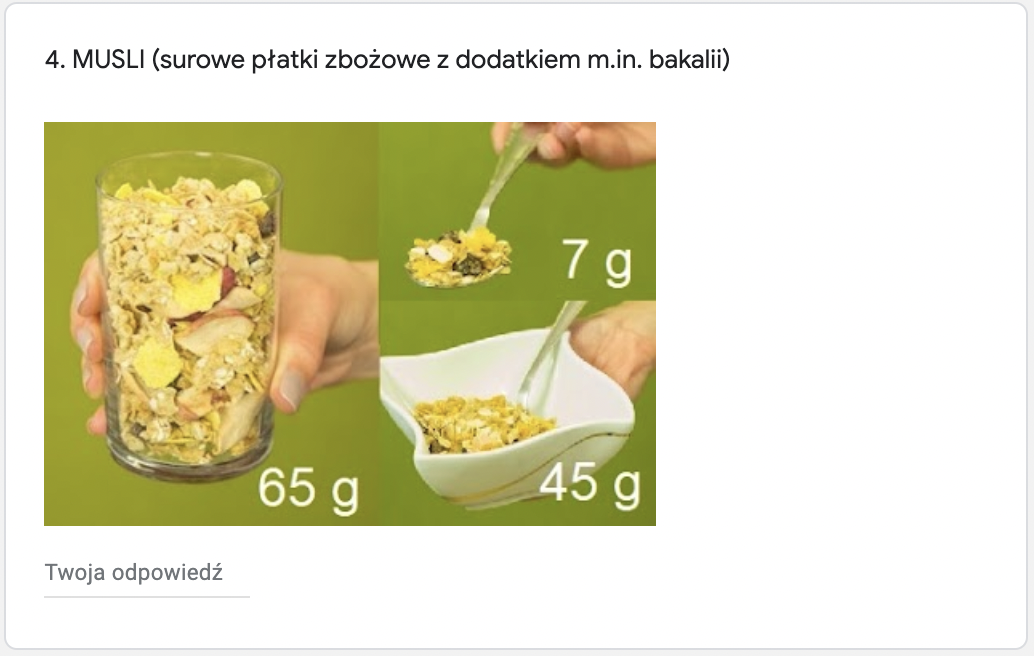


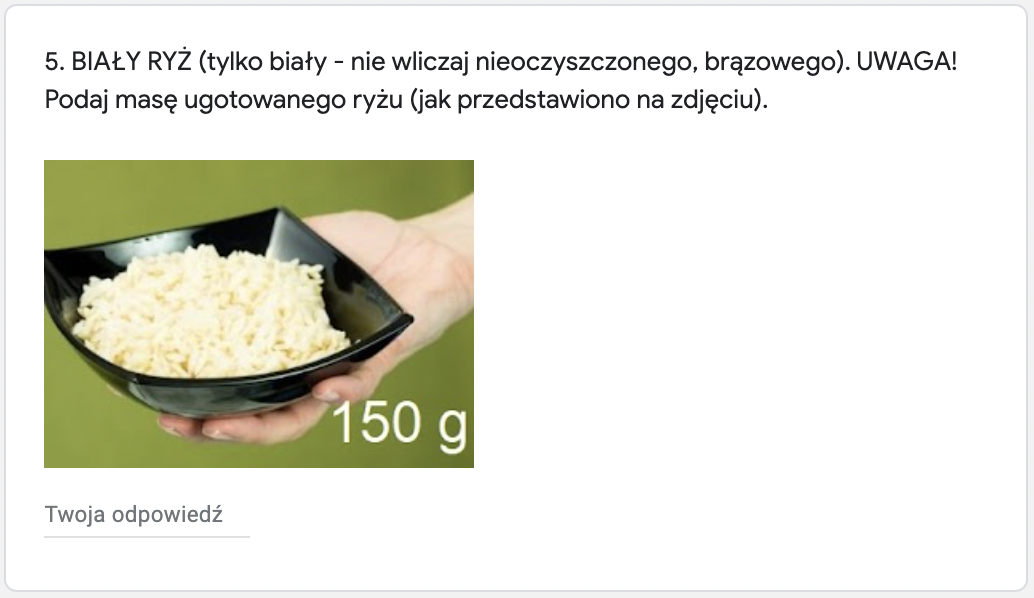


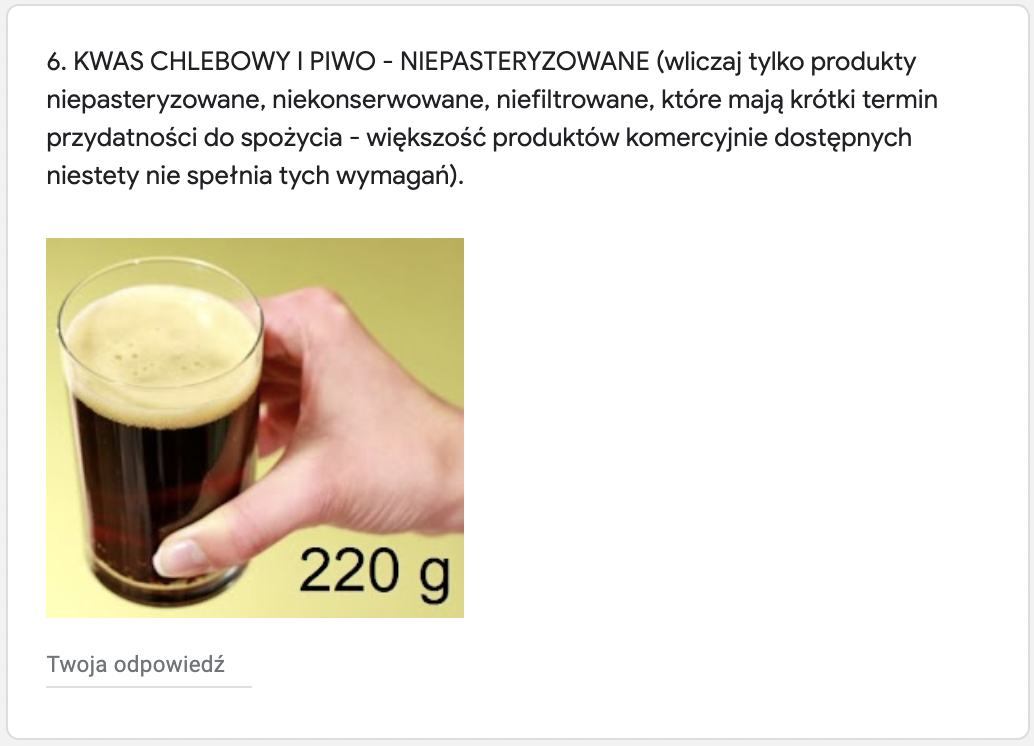


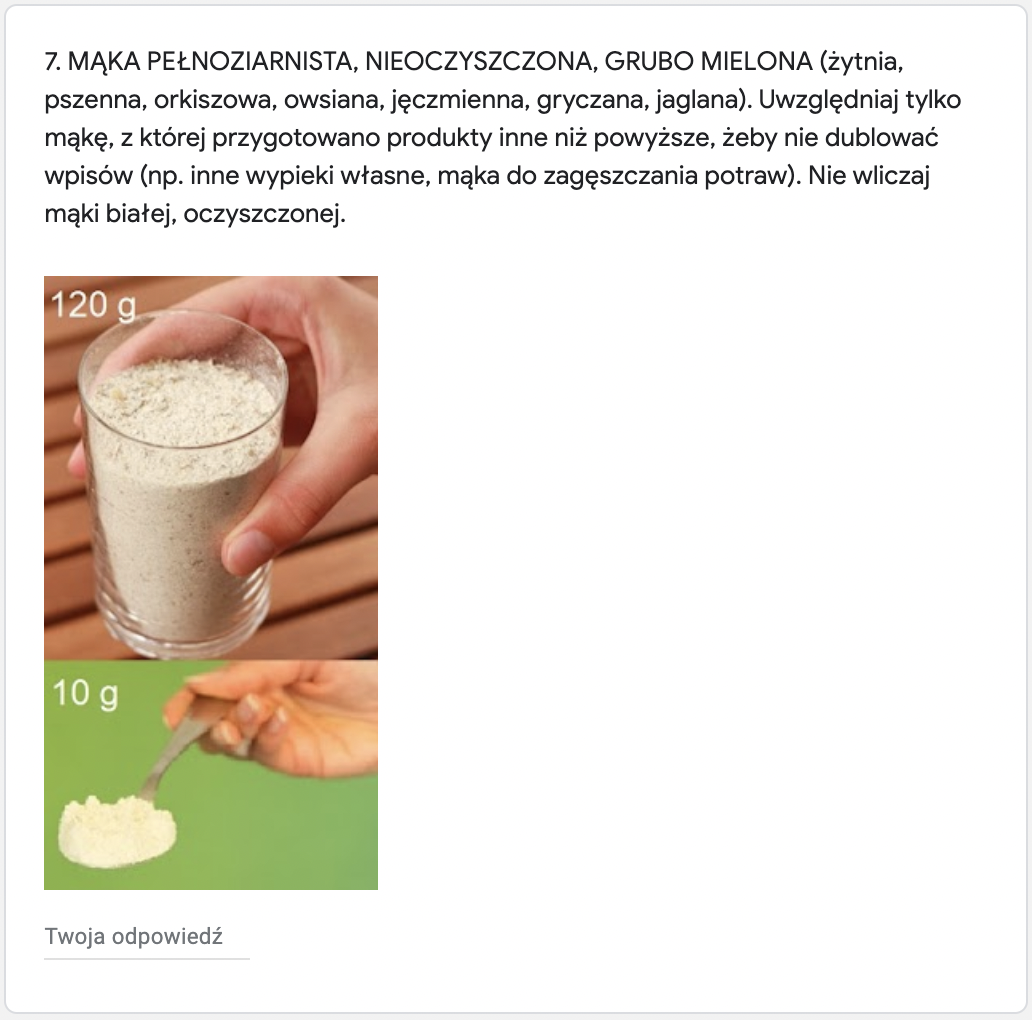


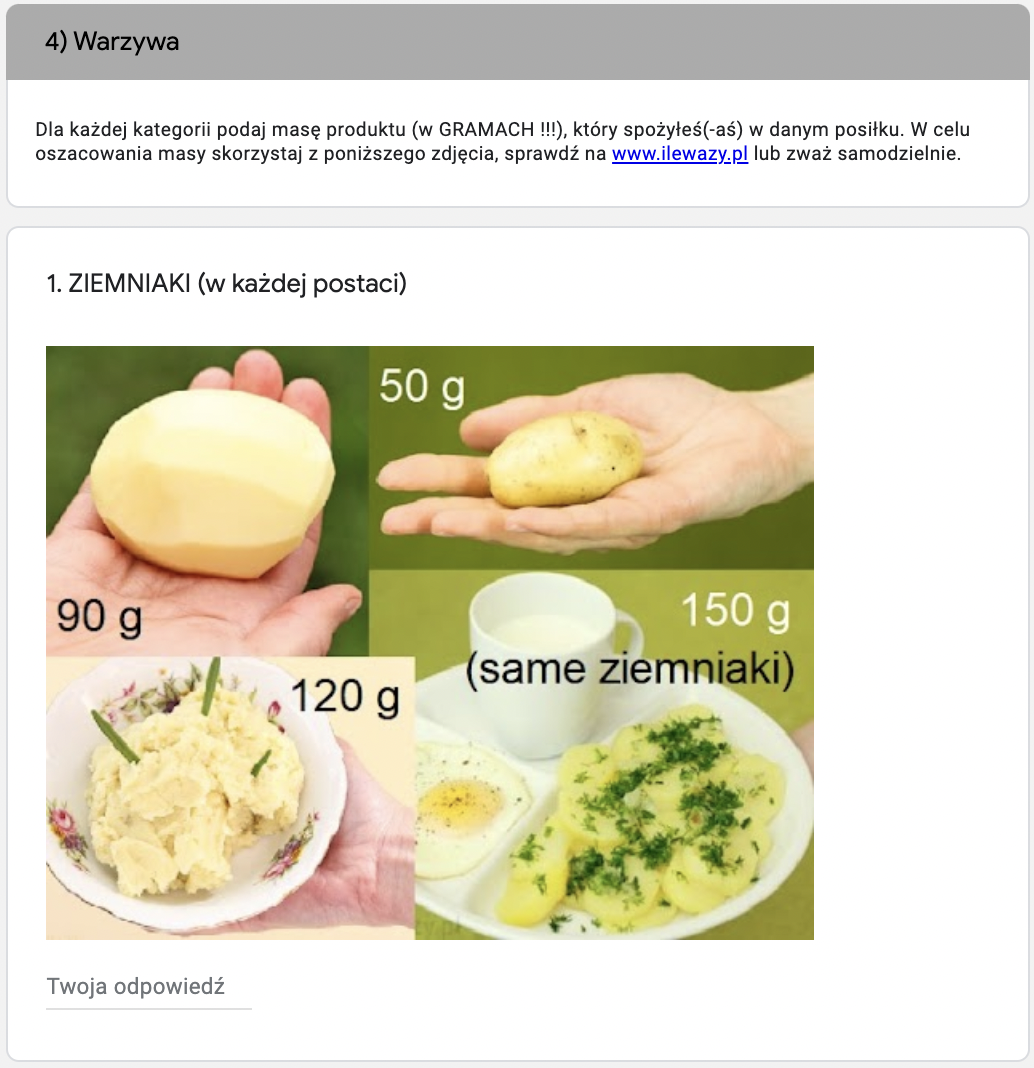


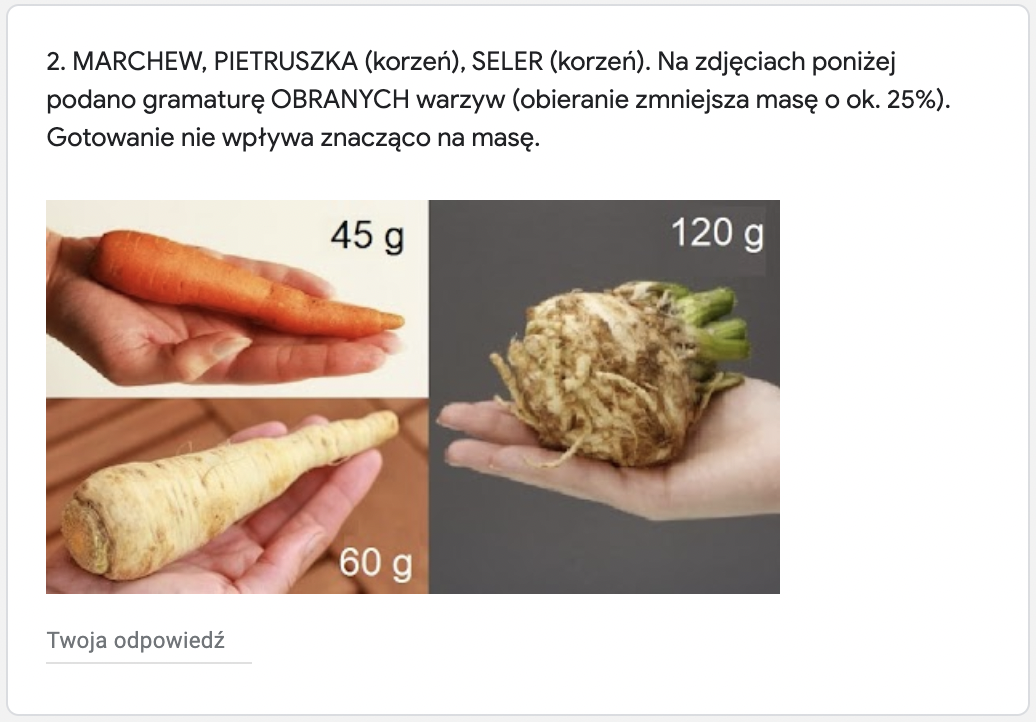


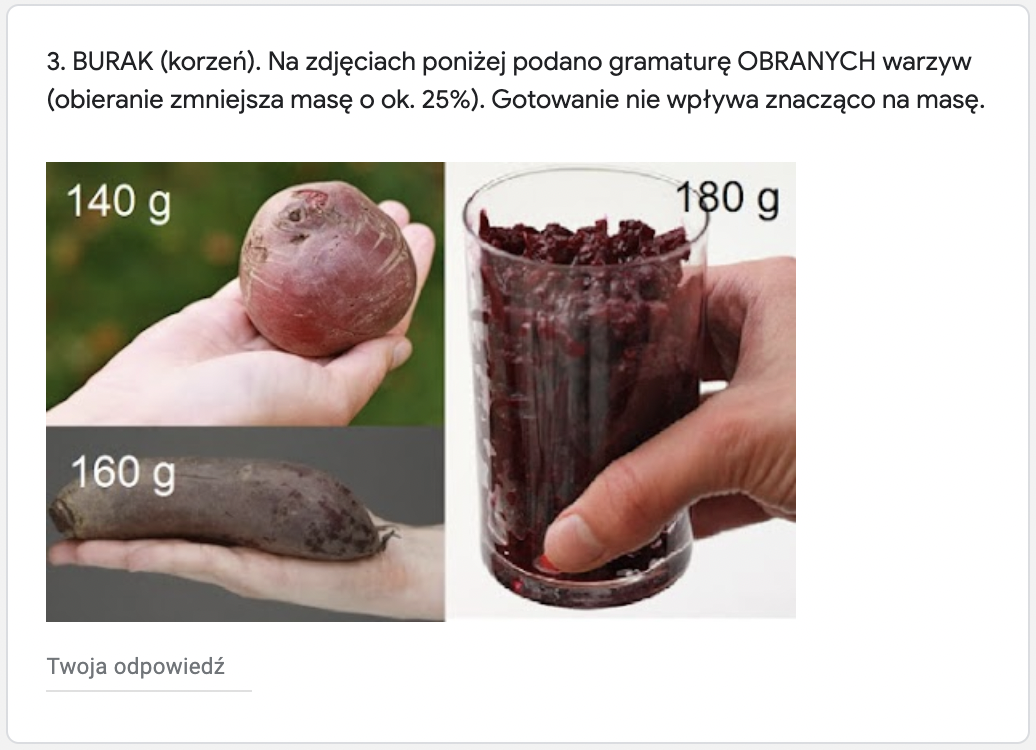


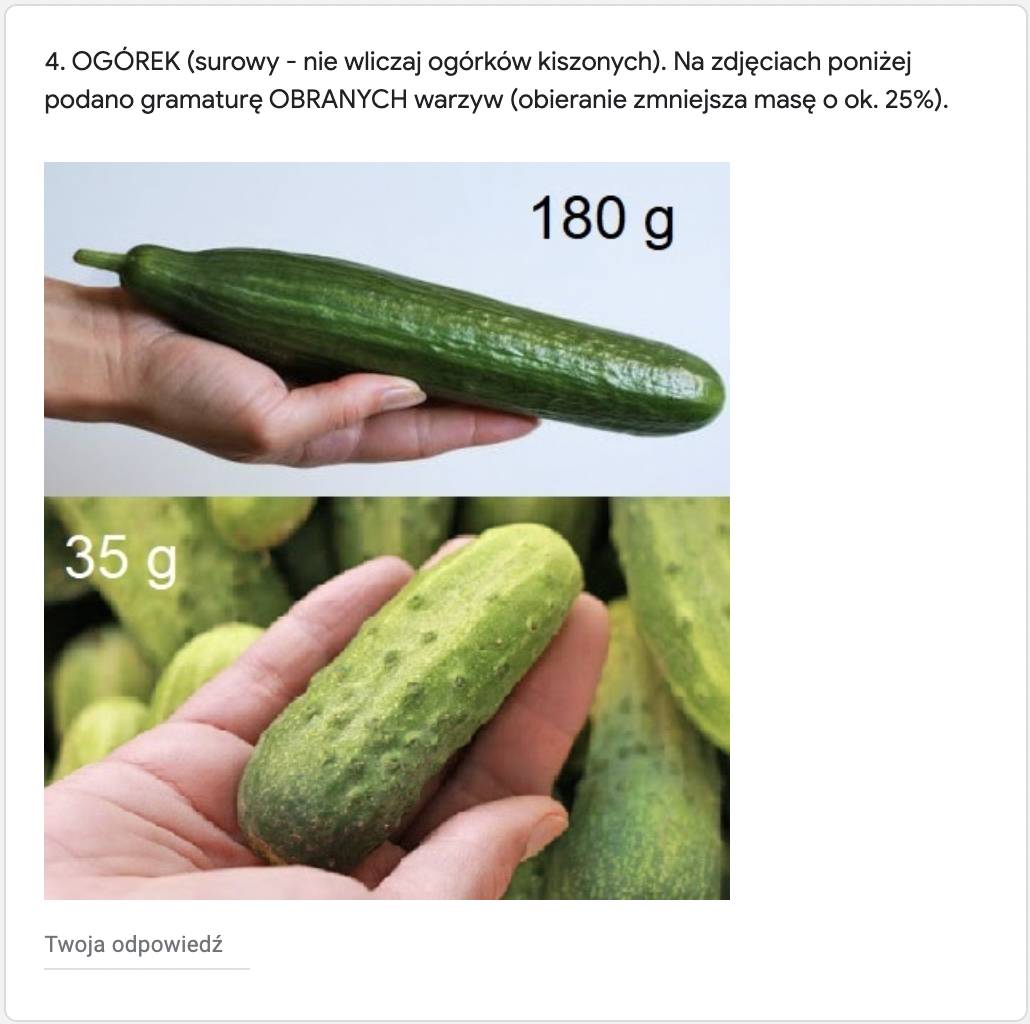


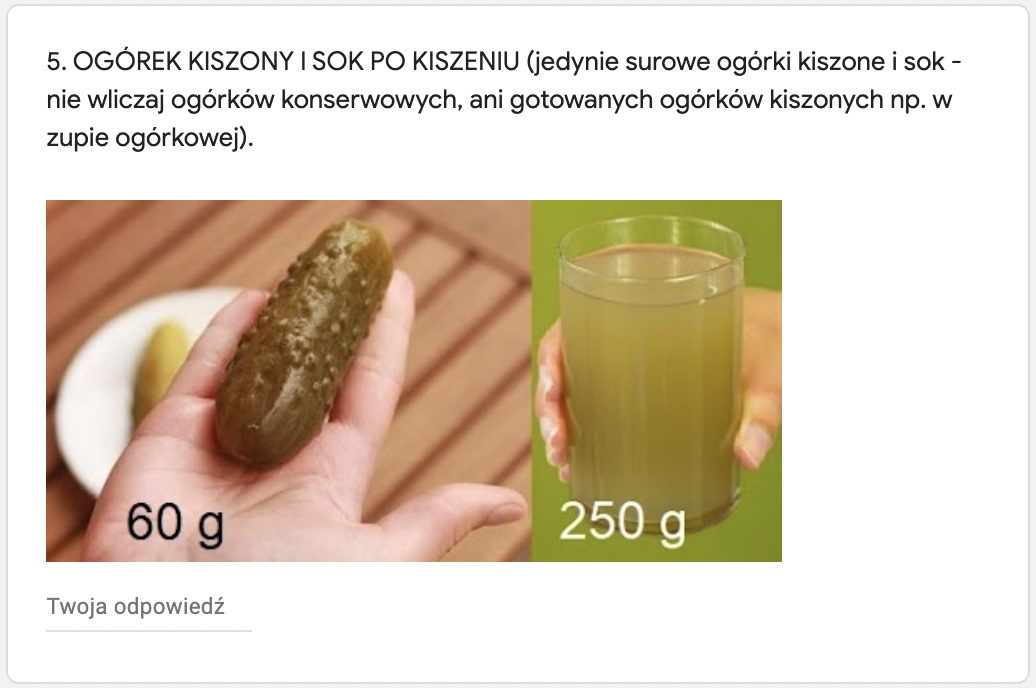


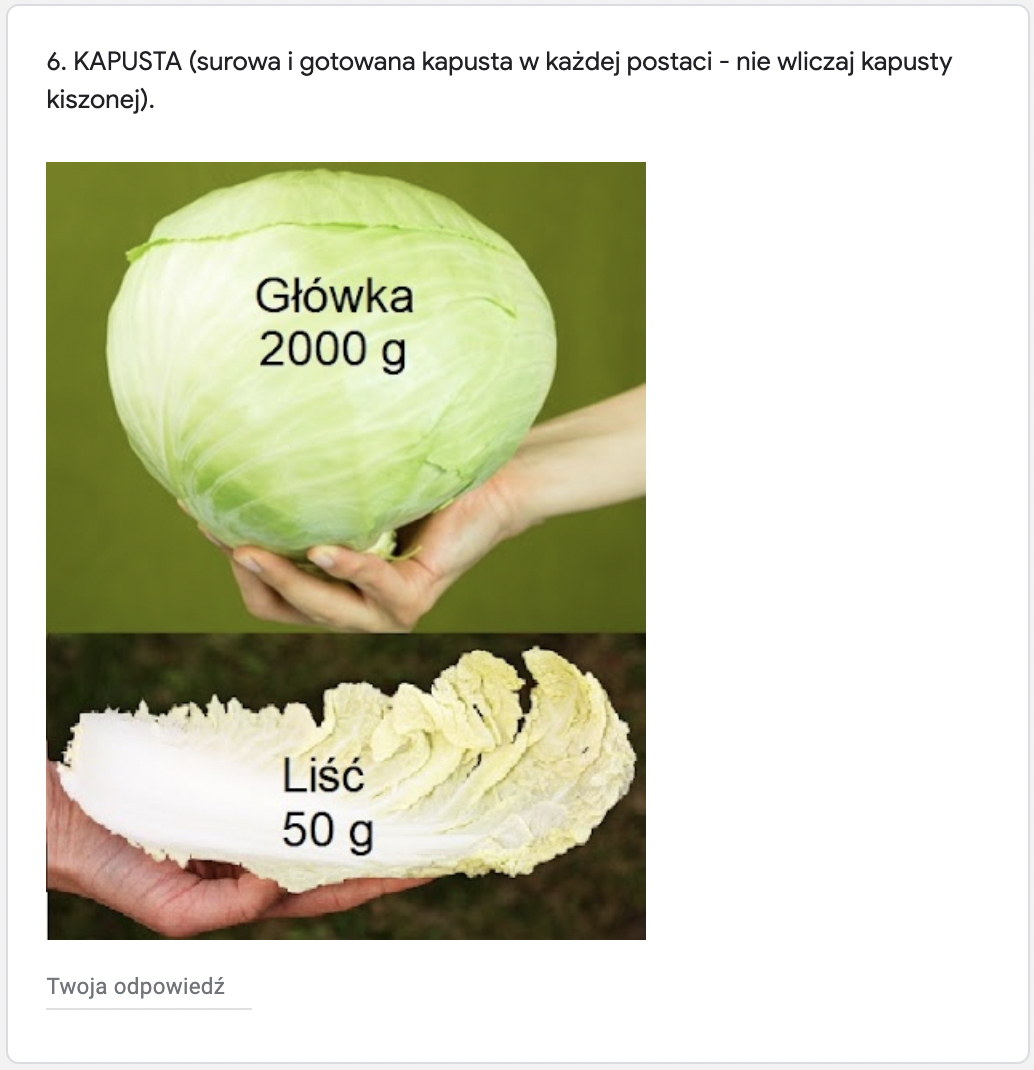


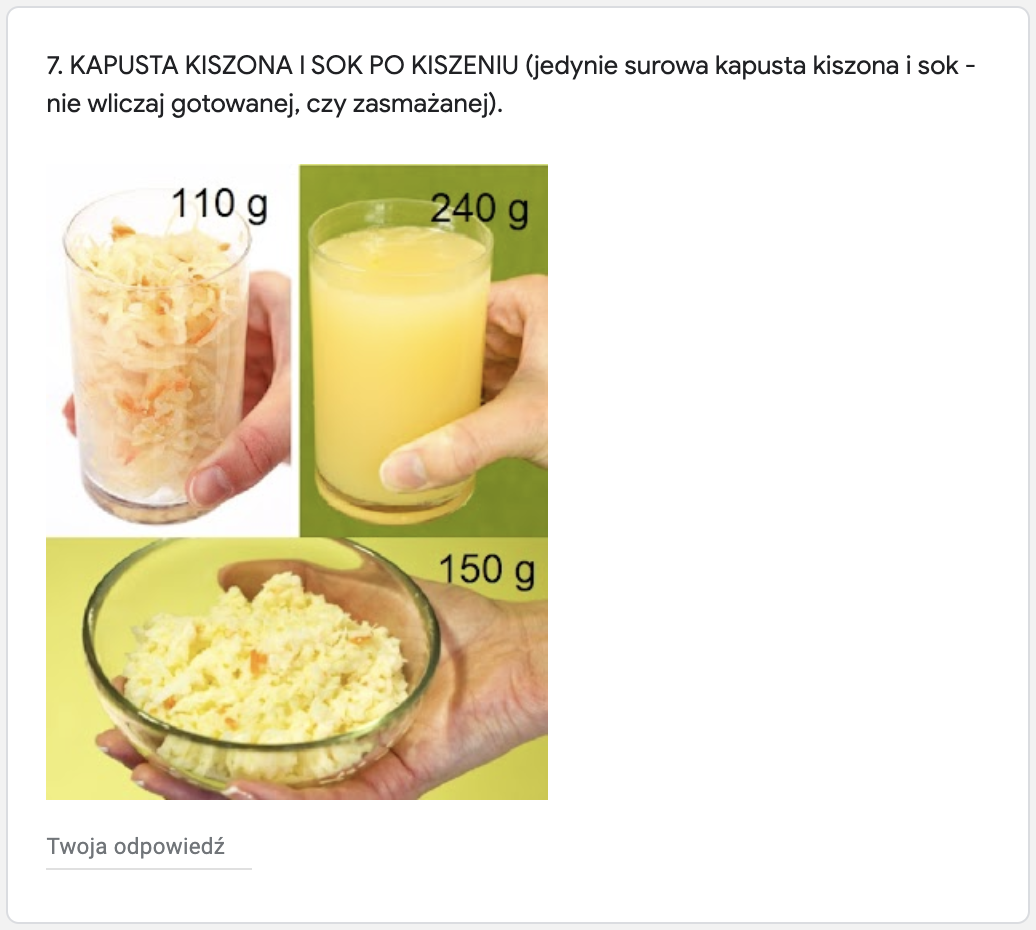


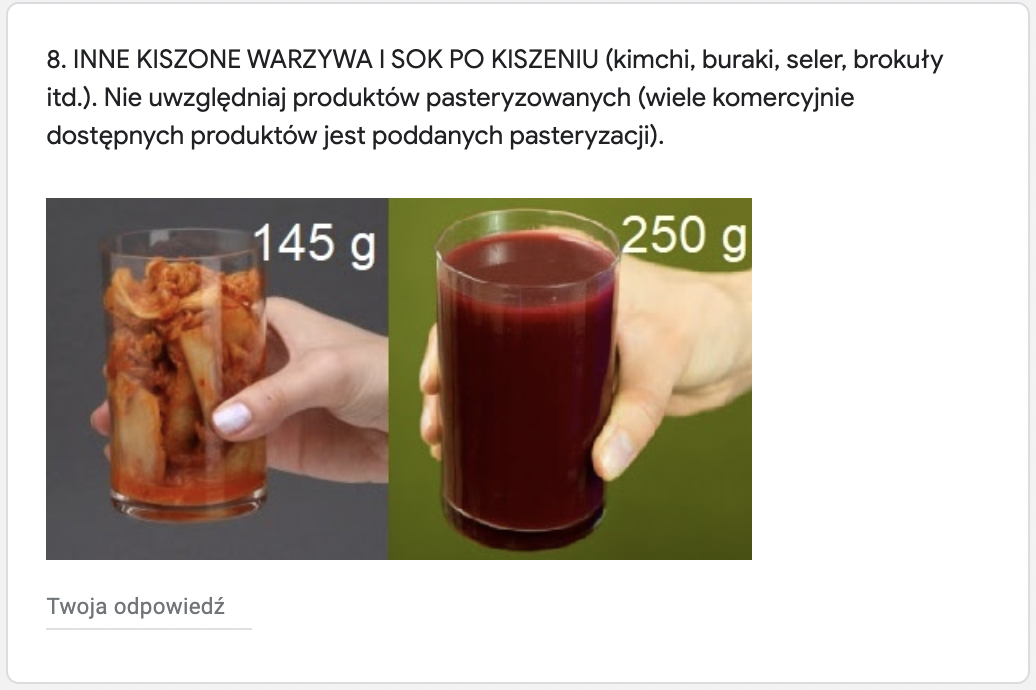


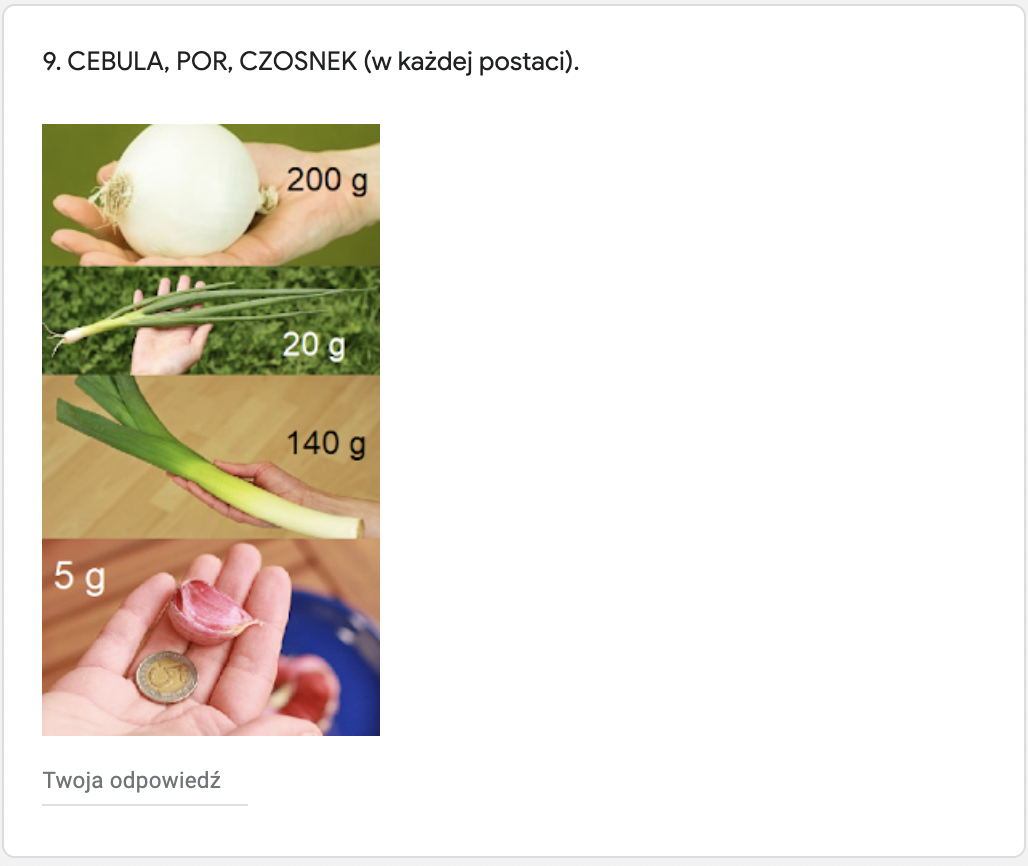


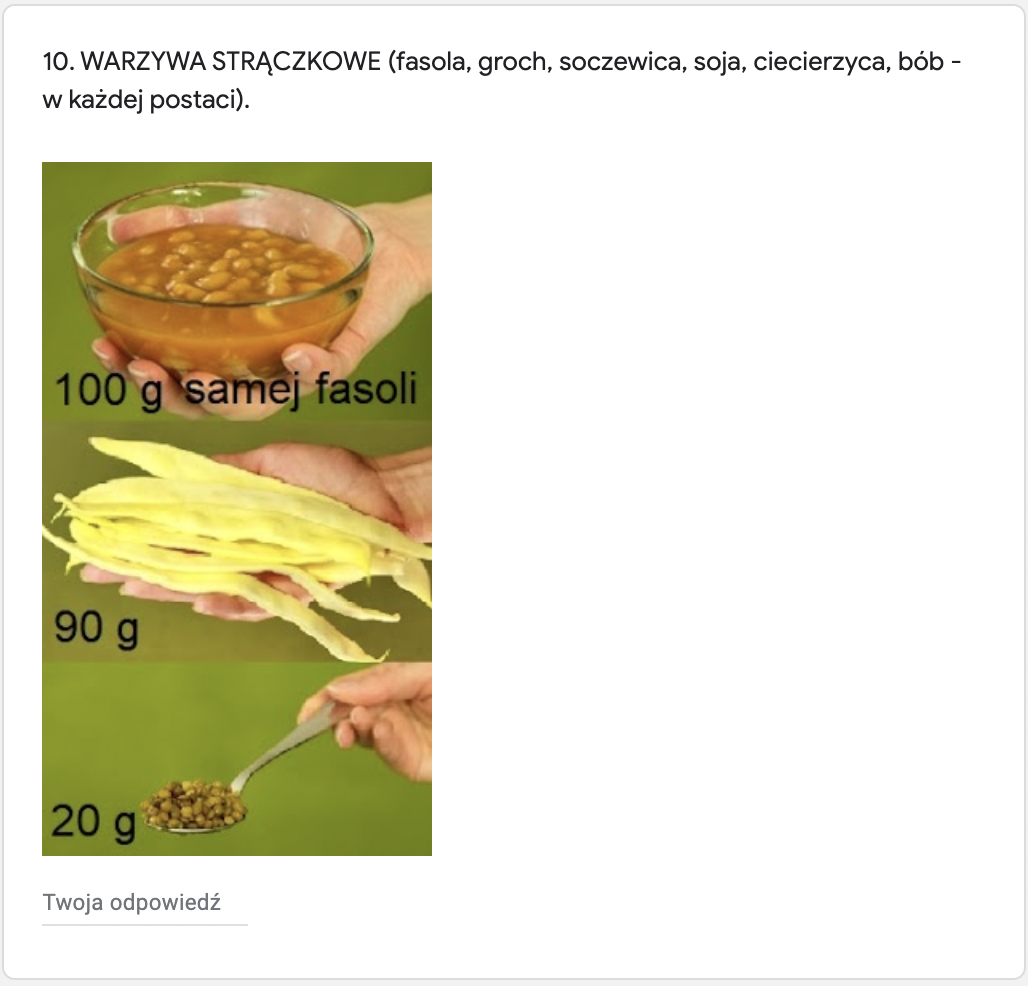


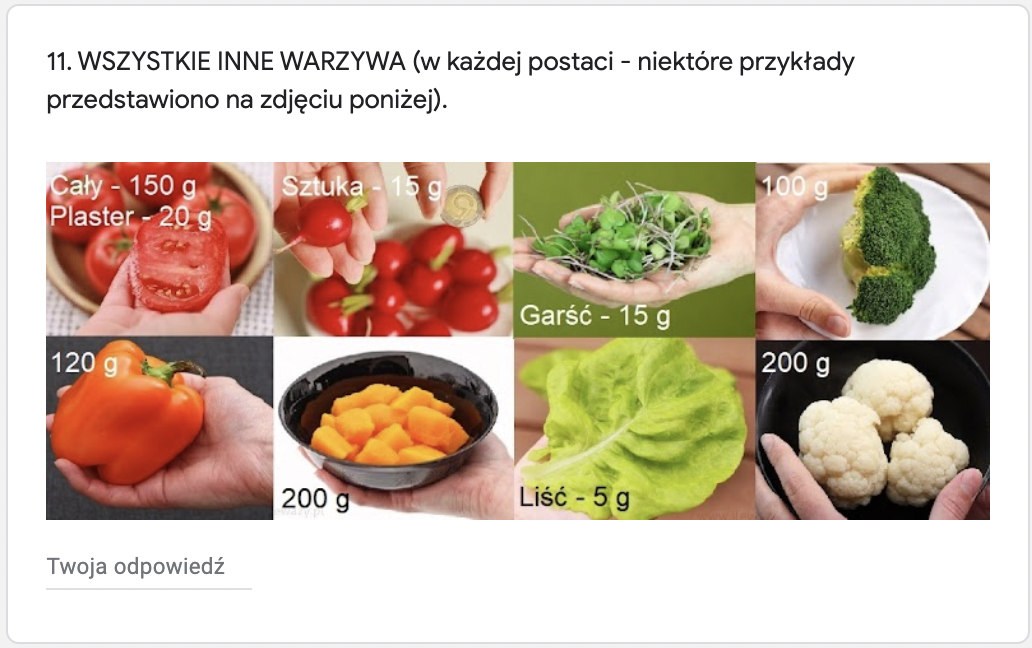


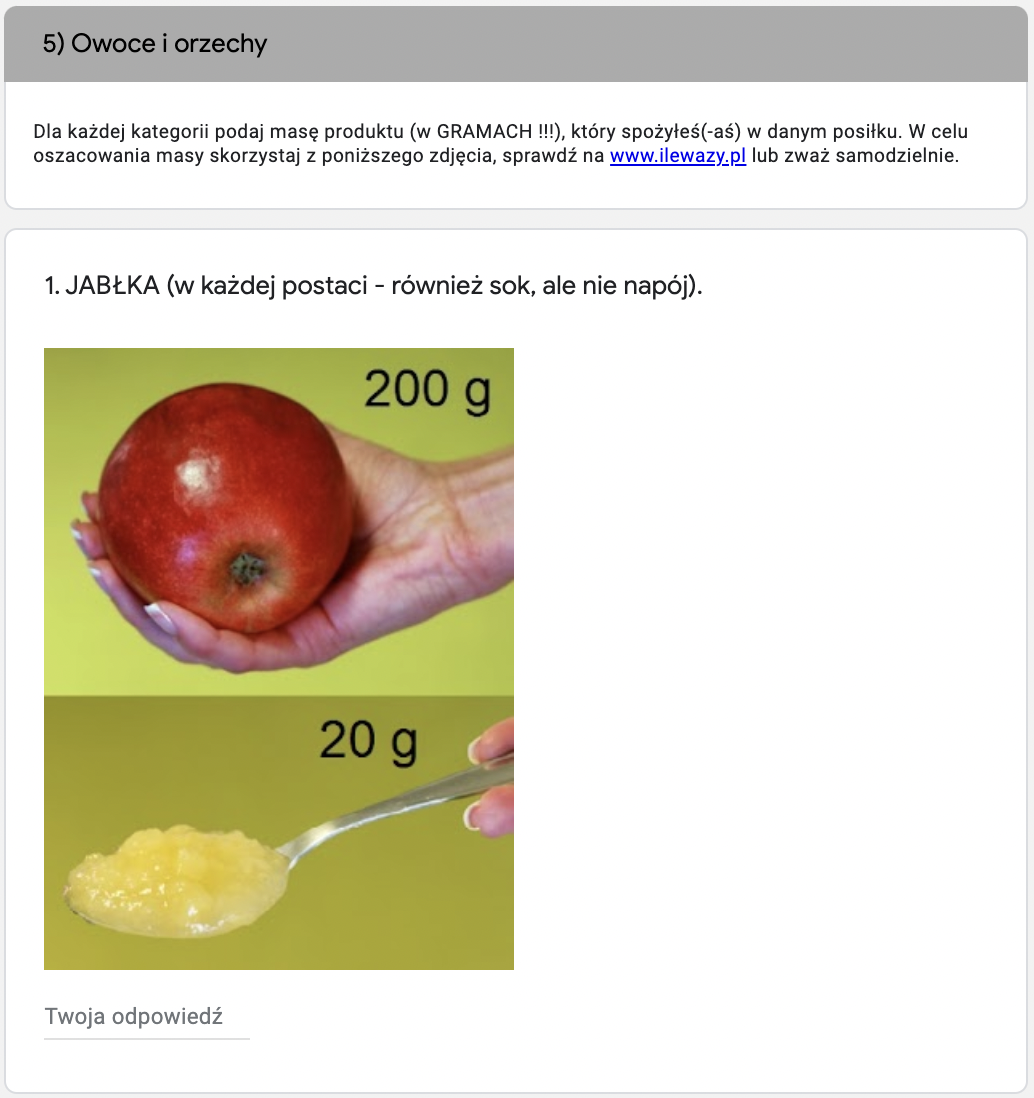


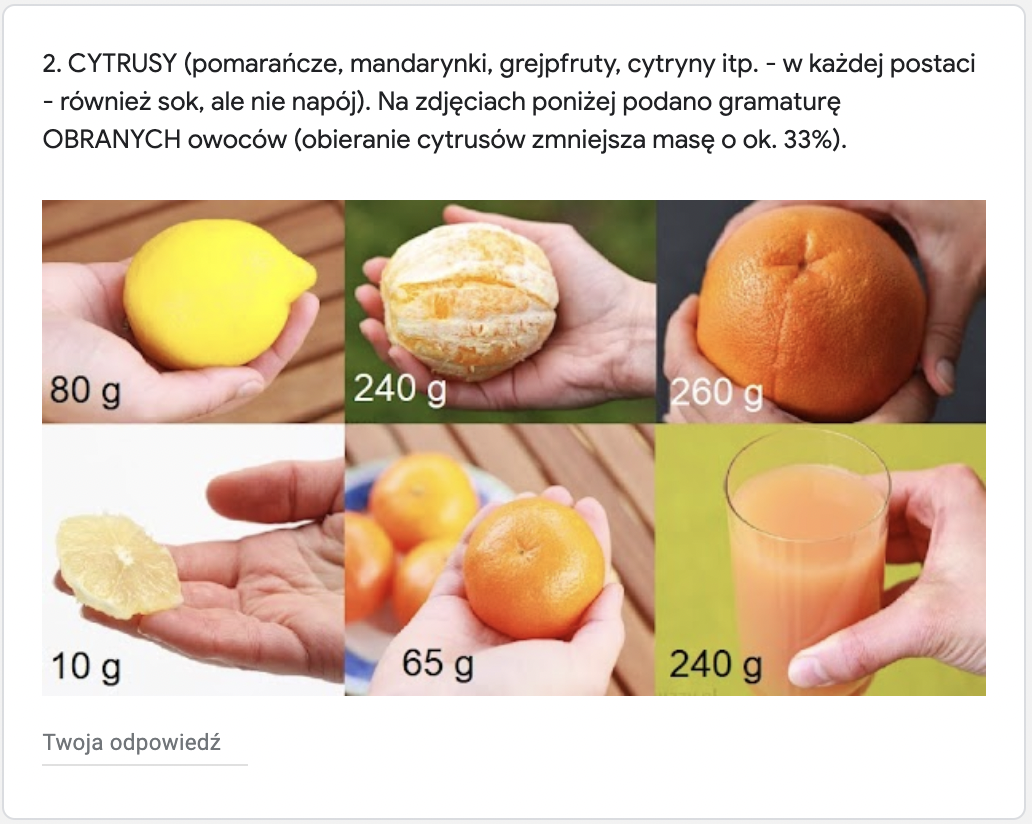


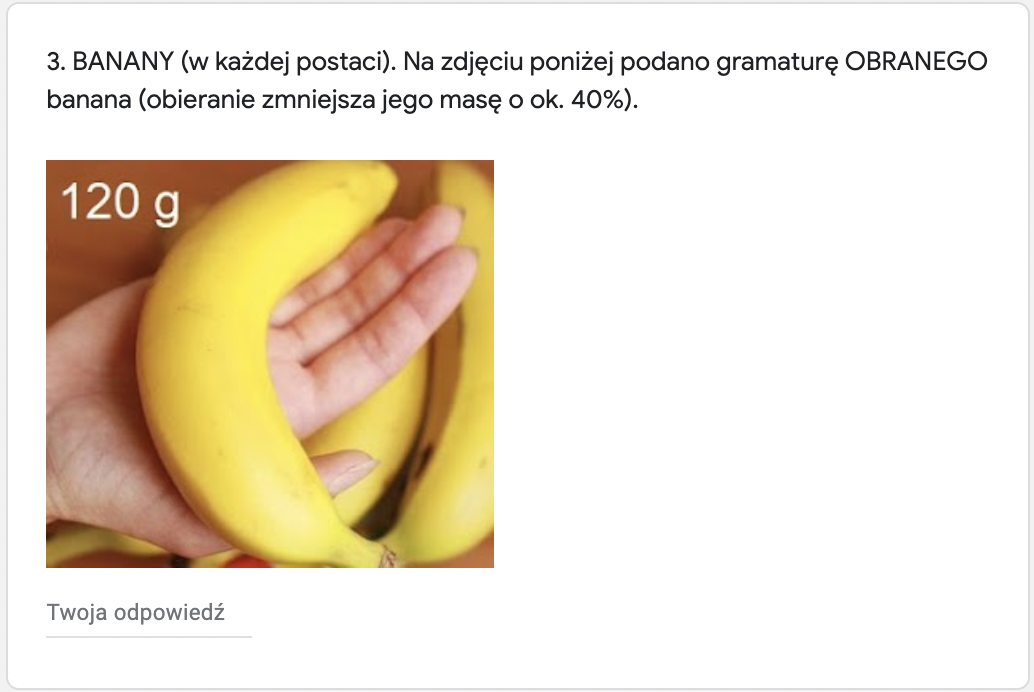


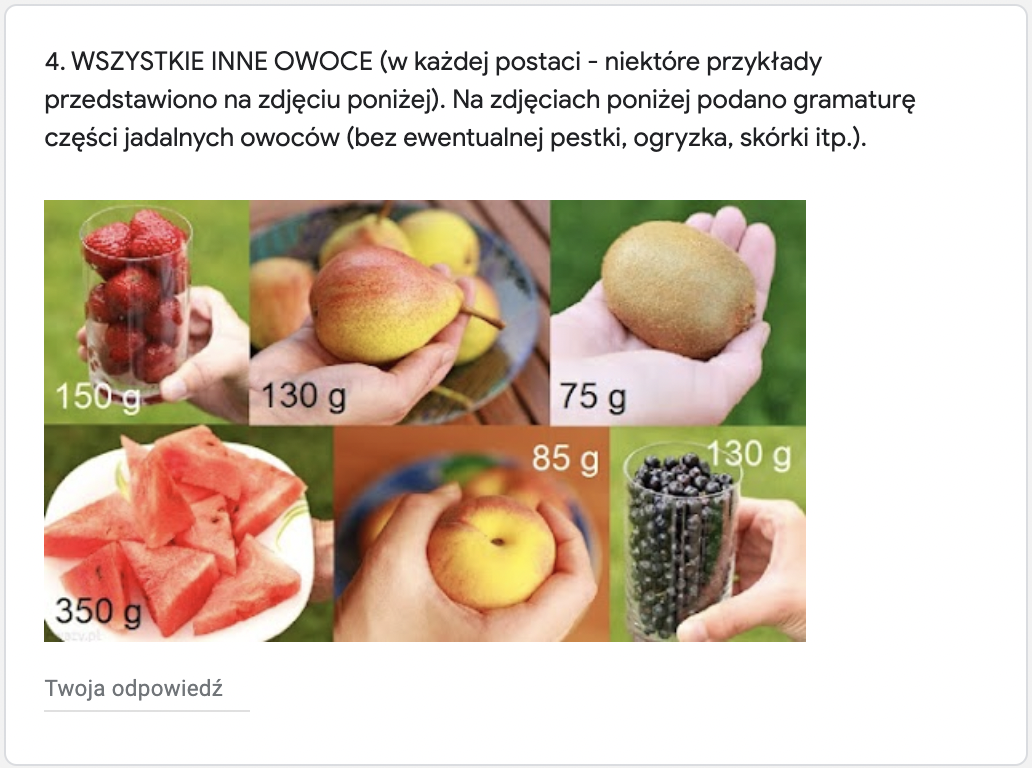

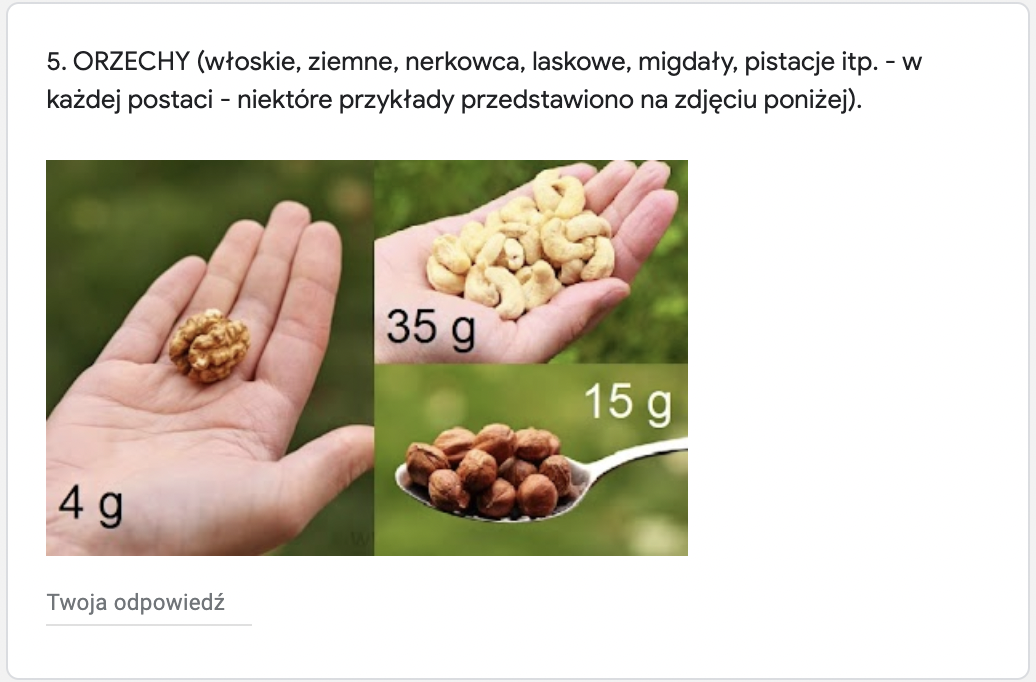

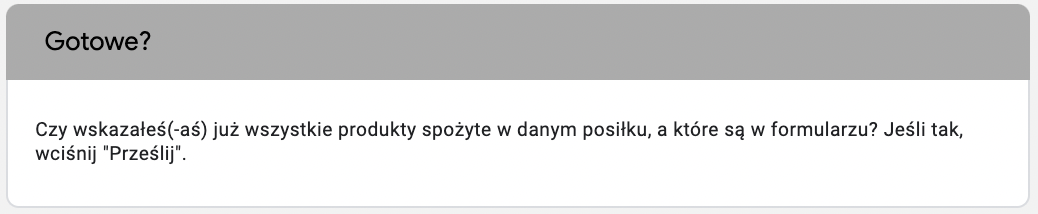


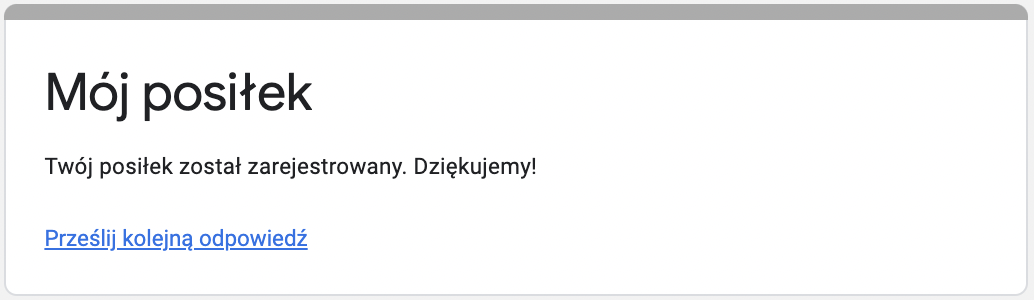


**English language translation of the original survey**

(The survey in the English language version is a free translation by the authors, it has not been validated for research use.)

**My meal**

- The form “My meal” is dedicated only to the participants of the research “Association between lifestyle factors and cognitive performance under stress” (ZarazFarma). The form was created to allow students of the Faculty of Medicine / Military Medicine to record what they eat for seven days.
- Please use this form to record which products you eat for a particular meal, and in what amounts.
- The form should be completed and sent every time during eating or directly after a meal. It is also possible to send a series of forms or one collective form at the end of the day (check how to report: <https://tinyurl.com/ZarazFarma-dieta-jak>).
- The form “My meal” classifies food into five categories, which will be visible in the further sections of the form:
  - meat, sausages, fish
  - dairy
  - grain products
  - vegetables
  - fruits and nuts
- The amount of food should be measured in grams (average masses in grams of single units of food will be shown in the photos for reference). Please, record only the mass of the products you eat, leave the rest of the form without any changes. If you eat any food not included in the form, just leave it and not record it anywhere (check how to assess a mass of food: <https://tinyurl.com/ZarazFarma-dieta-PRZYKLADY>)
- Do not record dietary supplements in tablets, capsules etc. in the form (however they are actually food).
- Moreover, write the number of your student record book at the beginning of the form.

**Number of student ID**

Make sure that you write the correct number!

**1)Meat, sausages, fishes…**

Write a mass (IN GRAMS!!!) of food that you eat in a particular meal. To assess a mass of food, use the photos below, check here: [www.ilewazy.pl](http://www.ilewazy.pl) or weight on your own.

1. RED MEAT (beef, pork, mutton, game, liver etc.) Include every form of red meat: units, cutlets, cold cuts, sausages etc.
2. WHITE MEAT (poultry). Include every form of white meat: units, cutlets, cold cuts, sausages etc.
3. FATTY FISH (halibut, salmon, mackerel, sardine, sprat, herring)
4. OTHER FISH
5. FISH OIL
6. SEAFOOD (shrimps, crabs, bivalve molluscs, oysters, snails ect.)

**2)Dairy**

Write a mass (IN GRAMS!!!) of food that you eat in a particular meal. To assess a mass of food, use the photos below, check here: [www.ilewazy.pl](http://www.ilewazy.pl) or weight on your own.

1. MILK (include every type of milk: cow’s milk, goat’s milk, sheep’s milk etc. - whole milk, semi-skimmed milk, skimmed milk - raw milk, pasteurised milk, boiled milk and milk as an addition to other meals). Do not include sour milk, fermented milk, yogurt, kefir nor cream.
2. COTTAGE CHEESE (cottage cheese - whole, semi-skimmed, skimmed). Do not include neither fermented cheese nor long-ripened cheese.
3. CHEESE (hard cheese - for example gouda cheese, cheddar cheese, parmesan cheese, mozzarella cheese - and soft cheese - for example brie cheese, camembert cheese). Do not include roasted, boiled, fried cheese nor cheese spread.
4. YOGURT, KEFIR, SOURED MILK (natural and sweet or flavored, commercial and home-made, whole and low-fat)
5. EGGS (hen’s egg - in every form)

**3) Grain products**

Write a mass (IN GRAMS!!!) of food that you eat in a particular meal. To assess a mass of food, use the photos below, check here: [www.ilewazy.pl](http://www.ilewazy.pl) or weight on your own.

1. LIGHT BREAD (white bread, bread rolls, toast bread, croissants etc.)
2. WHOLEMEAL BREAD, GRAHAM (made out of every kind of wholemeal flour or not-processed flour)
3. CEREAL, GROATS, WHOLE GRAIN NOODLE (rye, wheat, spelt, oat, groaning, groats, millet - only wholemeal, non-processed products). BE CAREFUL! Write only mass of boiled products (as it is shown in the photos).
4. MUESLI (raw cereal with dried tropical fruit added)
5. WHITE RICE (only white - do not include non-processed brown rice). BE CAREFUL! Write only mass of boiled rice (as it is shown in the photo).
6. UNPASTEURISED KVASS AND BEER - UNPASTEURISED (include only products which are unpasteurised, unpreserved, unfiltered and which have short expiry date - unfortunately, the majority of commercial products do not meet these criteria.
7. WHOLEMEAL FLOUR, non-processed flour, coarsely ground flour (rye, wheat, spelt, oat, groaning, groats, millet). Include only the flour from which other than the above mentioned products were made out. Due to it, your records are not double (for example include the flour you use to bake or to condense a sauce). Do not include plain flour nor processed flour.

**4) Vegetables**

Write a mass (IN GRAMS!!!) of food that you eat in a particular meal. To assess the mass of food, use the photos below, check here: [www.ilewazy.pl](http://www.ilewazy.pl) or weight them yourself.

1. POTATOES (in any form)
2. CARROT, PARSLEY (a root), CELERY (a root). In the photos below mass of peeled vegetables is shown (peeling vegetables causes a reduction of around 25% in their mass). Boiling does not change the mass significantly.
3. BEETROOT. In the photos below mass of peeled vegetables is shown (peeling vegetables causes a reduction of around 25% in their mass). Boiling does not change the mass significantly.
4. CUCUMBER (raw cucumber - do not include pickled cucumbers). In the photos below mass of peeled vegetables is shown (peeling vegetables causes a reduction of around 25% in their mass).
5. PICKLED CUCUMBER AND PICKLING JUICE (only raw pickled cucumbers and pickling juice - do not include gherkins nor boiled pickled cucumbers for example in cucumber soup).
6. CABBAGE (raw and boiled cabbage in every form - do not include sauerkraut).
7. SAUERKRAUT AND PICKLING JUICE (only raw sauerkraut and pickling juice - do not include boiled sauerkraut nor fried sauerkraut).
8. OTHER FERMENTED VEGETABLES AND THEIR PICKLING JUICE (kimchi, beetroots, celeries, broccoli etc.). Do not include pasteurised products (many commercial products are pasteurized).
9. ONION, LEEK, GARLIC (in every form).
10. LEGUMINOUS VEGETABLES (beans, peas, lentils, soybeans, chickpeas, broad beans - in every form).
11. ALL OTHER VEGETABLES (in every form - some examples are presented below).

**5)Fruits and nuts**

Write a mass (IN GRAMS!!!) of food that you eat in a particular meal. To assess a mass of food, use the photos below, check here: [www.ilewazy.pl](http://www.ilewazy.pl) or weight on your own.

1. APPLES (in every form - also juice, but not apple cider).
2. CITRUSES (oranges, mandarins, grapefruits, lemons etc. - in every form - also fruit juice, but not fruit drink). In the photos below mass of peeled fruits is shown (peeling fruits causes a reduction of around 33% in their mass).
3. BANANAS (in every form). Mass of peeled banana is shown in the photo below (peeling bananas causes a reduction of around 40% in their mass).
4. ALL OTHER FRUITS (in every form - some examples are presented below). Mass of edible parts of fruits is shown in the photos below (without stones, apple cores, skins etc.).
5. NUTS (walnuts, peanuts, cashews, hazelnuts, almonds, pistachio nuts etc. - in every form - some examples are presented below).

**Are you ready?**

Did you record every product that you had eaten which was available in this form? If yes, click the “send” button.

**Your meal**

Your meal was recorded. Thank you!
