# Supplementary material for: Association Between Consumption of Fermented Food and Food-Derived Prebiotics With Cognitive Performance, Depressive, and Anxiety Symptoms in Psychiatrically Healthy Medical Students Under Psychological Stress: A Prospective Cohort Study
Source: Front Nutr. 2022 Mar 3;9:850249. doi: 10.3389/fnut.2022.850249 (PMC8929173; doi:10.3389/fnut.2022.850249)
Supplement: Supplementary file 4 [file Data_Sheet_4.DOCX]

***Supplementary Material 4***

***Estimation of inulin and fructooligosaccharides (IN&FOS) consumption***

IN&FOS consumption was calculated by multiplying the quantity of each consumed prebiotic-containing food by a factor reflecting IN&FOS content as presented in the Table below:

$IN\&FOS consumption=\sum(Factor \times Quantity of prebiotic food$)

| No ^a^ | Prebiotic-containing food | Factor reflecting IN&FOS content ^b^ | Explanation |
| --- | --- | --- | --- |
| 3b | Wholemeal bread and graham | 0.019 | Assuming 30% wheat flour (midpoint for baked wheat flour: 0.048) and 30% rye flour (midpoint for baked rye: 0.014)  0.3 × 0.048 + 0.3 × 0.014 = 0.019 |
| 3c | Cereal, groats and whole grain noodle | 0.015 | Assuming rye (midpoint for baked rye: 0.014) and barley (midpoint for raw barley 0.016) in 1:1 mass proportion  0.5 × 0.014 + 0.5 × 0.016 = 0.015 |
| 3d | Muesli | 0.010 | Assuming the content of 2/3 of rye and barley (1:1 mass proportion) grains  0.67 × “3c” = 0.010 |
| 3g | Wholemeal flour | 0.032 | Assuming wheat (midpoint for bran-raw wheat 0.050) and rye (midpoint for baked rye: 0.014) data in 1:1 mass proportion  0.5 × 0.050 + 0.5 × 0.014 = 0.032 |
| 4i | Onion, leek and garlic | 0.092 | Assuming consumption of raw onions, leeks and garlic cloves in 1:¼:1 item proportion ^c^  (200 × 0.086 + 0.25 × 140 × 0.117 + 5 × 0.175) / (200 + 0.25 × 140 + 5) = 0.092 |
| 5c | Bananas (raw) | 0.010 | Raw banana 0.010 |
| S2 | Asparagus (raw or boiled) | 0.042 | Assuming equal contribution of raw and boiled vegetable  0.5 × 0.050 + 0.5 × 0.034 = 0.042 |
| S2 | Chicory  root | 0.645 |  |
| S2 | Dandelion  leaves (raw or cooked) | 0.204 | Assuming equal contribution of raw and boiled vegetable  0.5 × 0.243 + 0.5 × 0.164 = 0.204 |
| S2 | Globe artichoke | 0.048 |  |
| S2 | Jerusalem artichoke | 0.315 |  |

S2 – as evaluated in Survey 2

^a^ Number in the Food Record form or other source of data

^b^ IN&FOS content at the basis of Moshfegh et al. (1999)

^c^ onion, leak and garlic consumption estimated at the basis of Dorant et al. (1996)

**References**

Dorant, E., van den Brandt, P. A., & Goldbohm, R. A. (1996). A prospective cohort study on the relationship between onion and leek consumption, garlic supplement use and the risk of colorectal carcinoma in The Netherlands. *Carcinogenesis*, *17*(3), 477–484. https://doi.org/10.1093/carcin/17.3.477

Moshfegh, A. J., Friday, J. E., Goldman, J. P., & Ahuja, J. K. (1999). Presence of inulin and oligofructose in the diets of Americans. *The Journal of Nutrition*, *129*(7 Suppl), 1407S-11S. https://doi.org/10.1093/jn/129.7.1407S
