# Supplementary material for: Association Between Consumption of Fermented Food and Food-Derived Prebiotics With Cognitive Performance, Depressive, and Anxiety Symptoms in Psychiatrically Healthy Medical Students Under Psychological Stress: A Prospective Cohort Study
Source: Front Nutr. 2022 Mar 3;9:850249. doi: 10.3389/fnut.2022.850249 (PMC8929173; doi:10.3389/fnut.2022.850249)
Supplement: Supplementary file 5 [file Data_Sheet_5.DOCX]

***Supplementary Material 5***

***Survey 2***

The screenshots of the original survey are presented below.


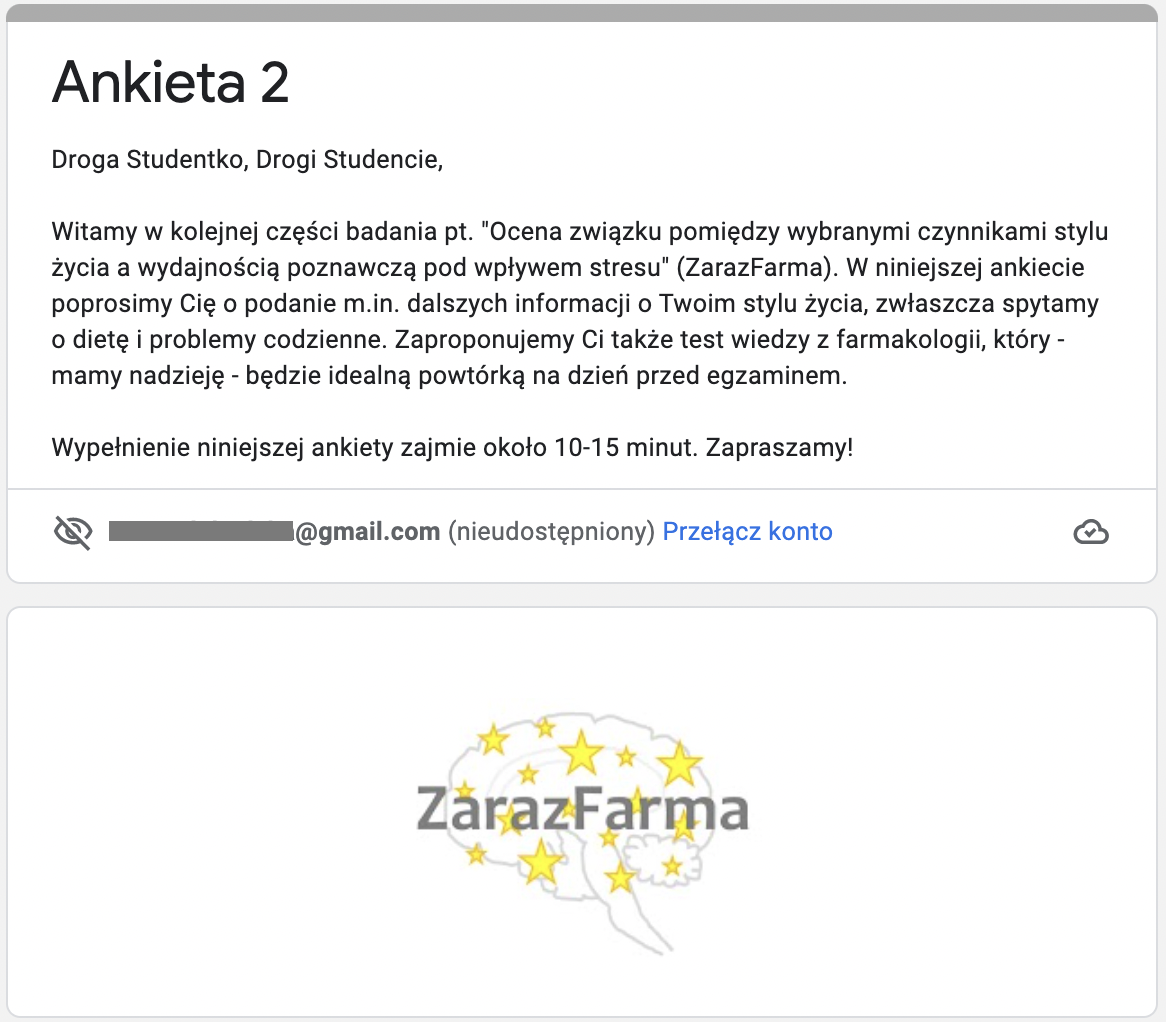


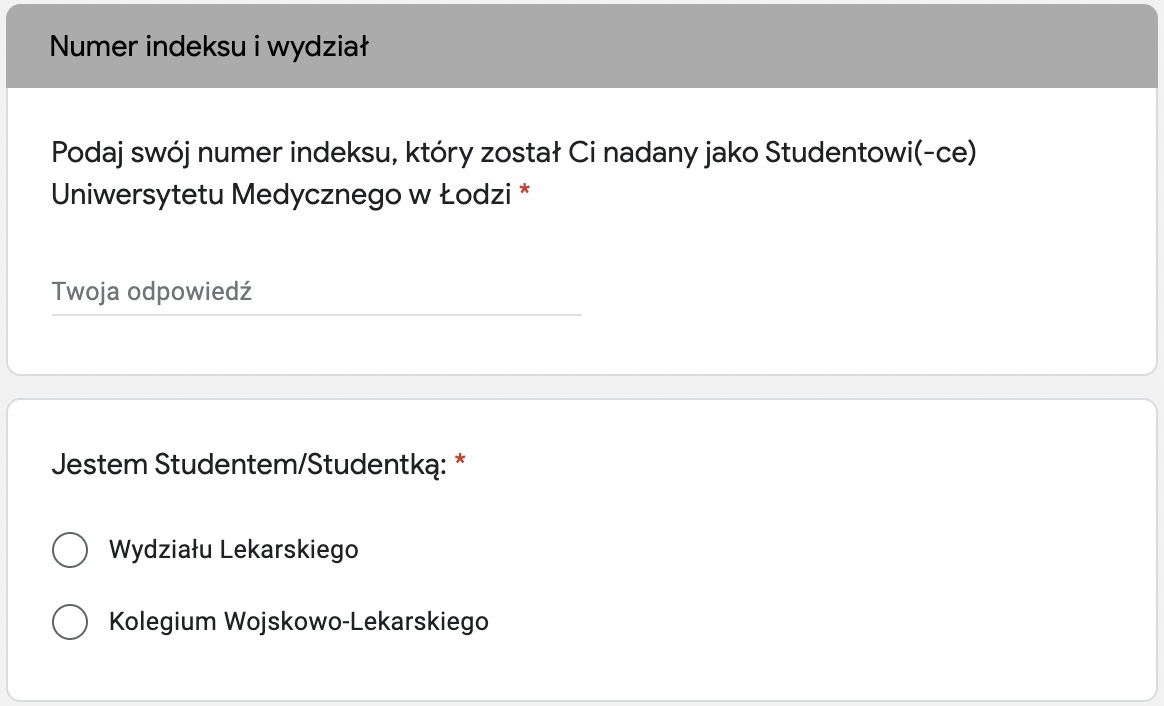


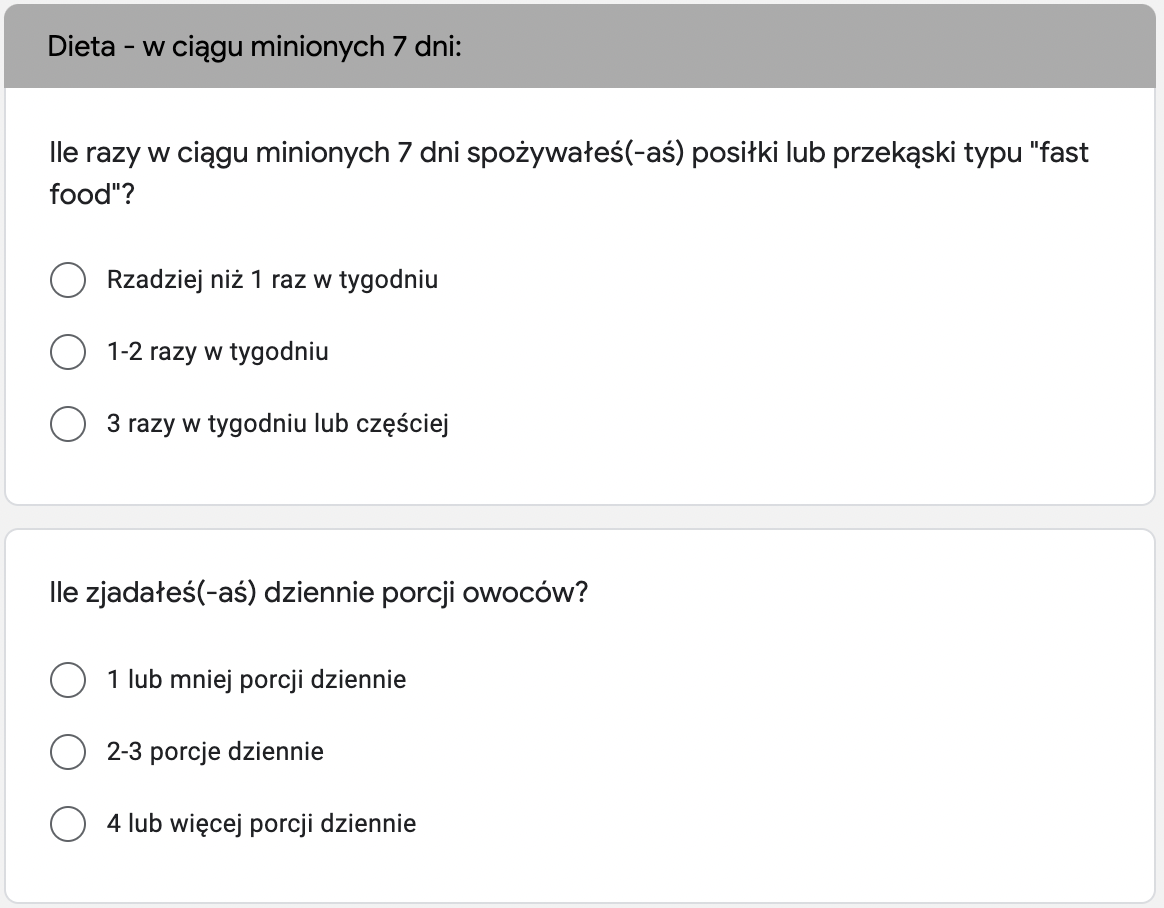


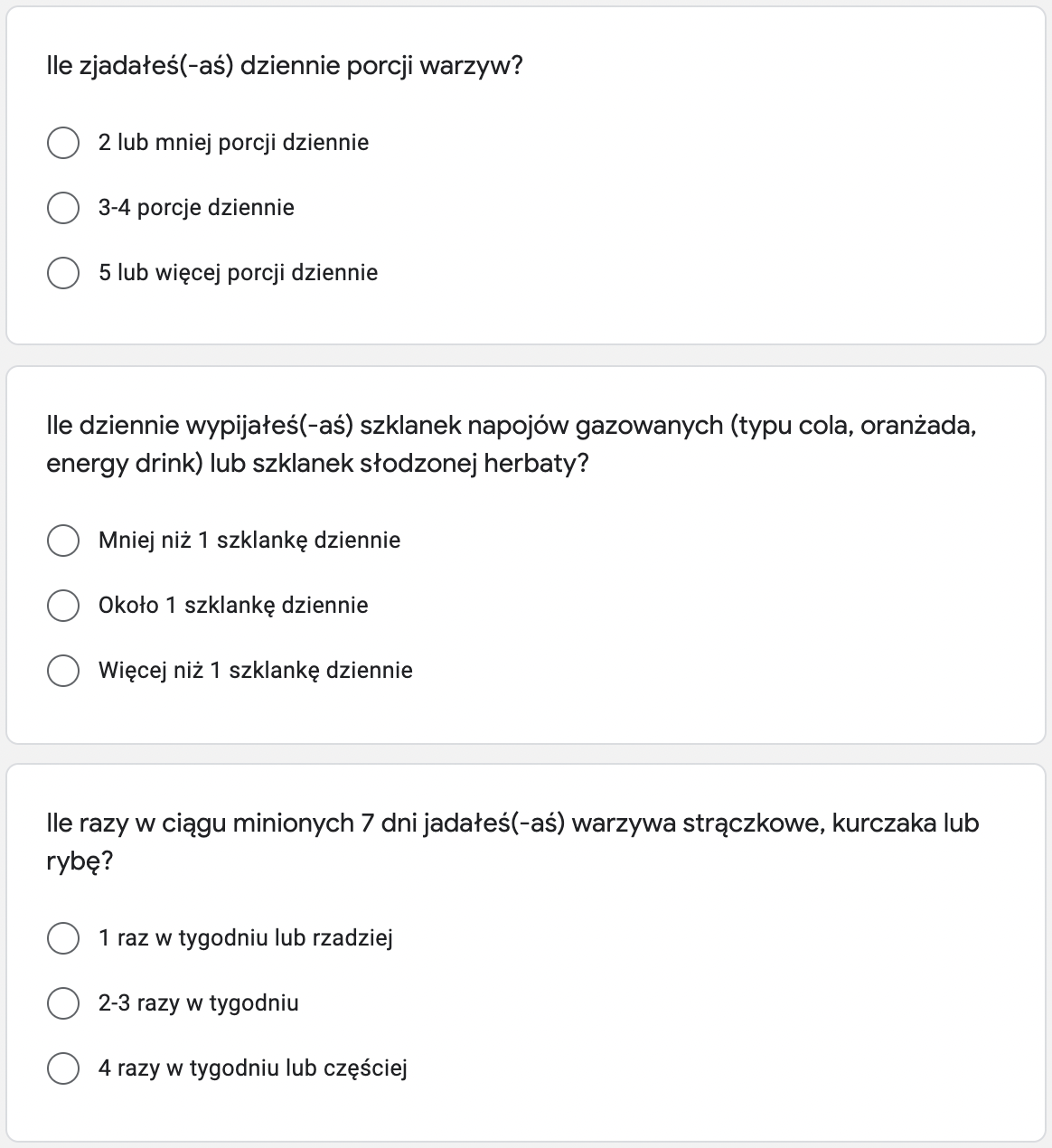


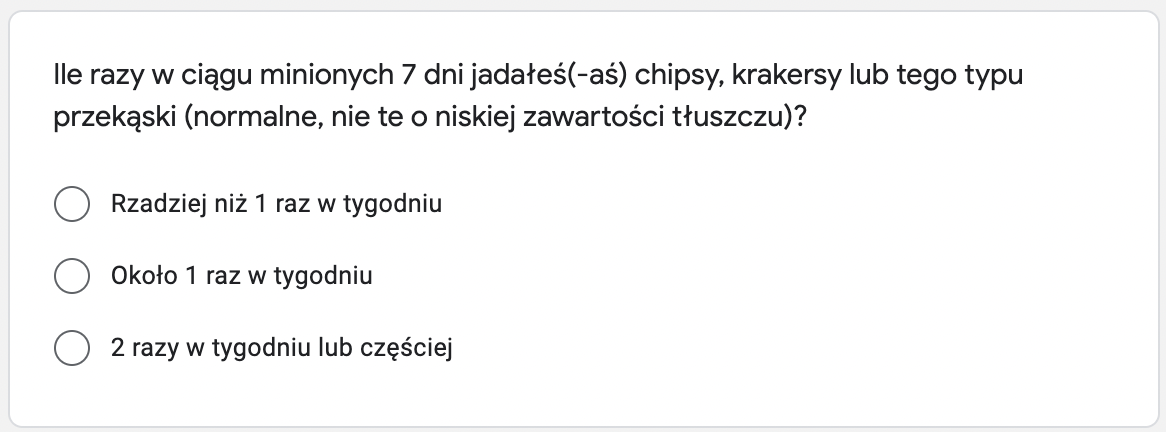


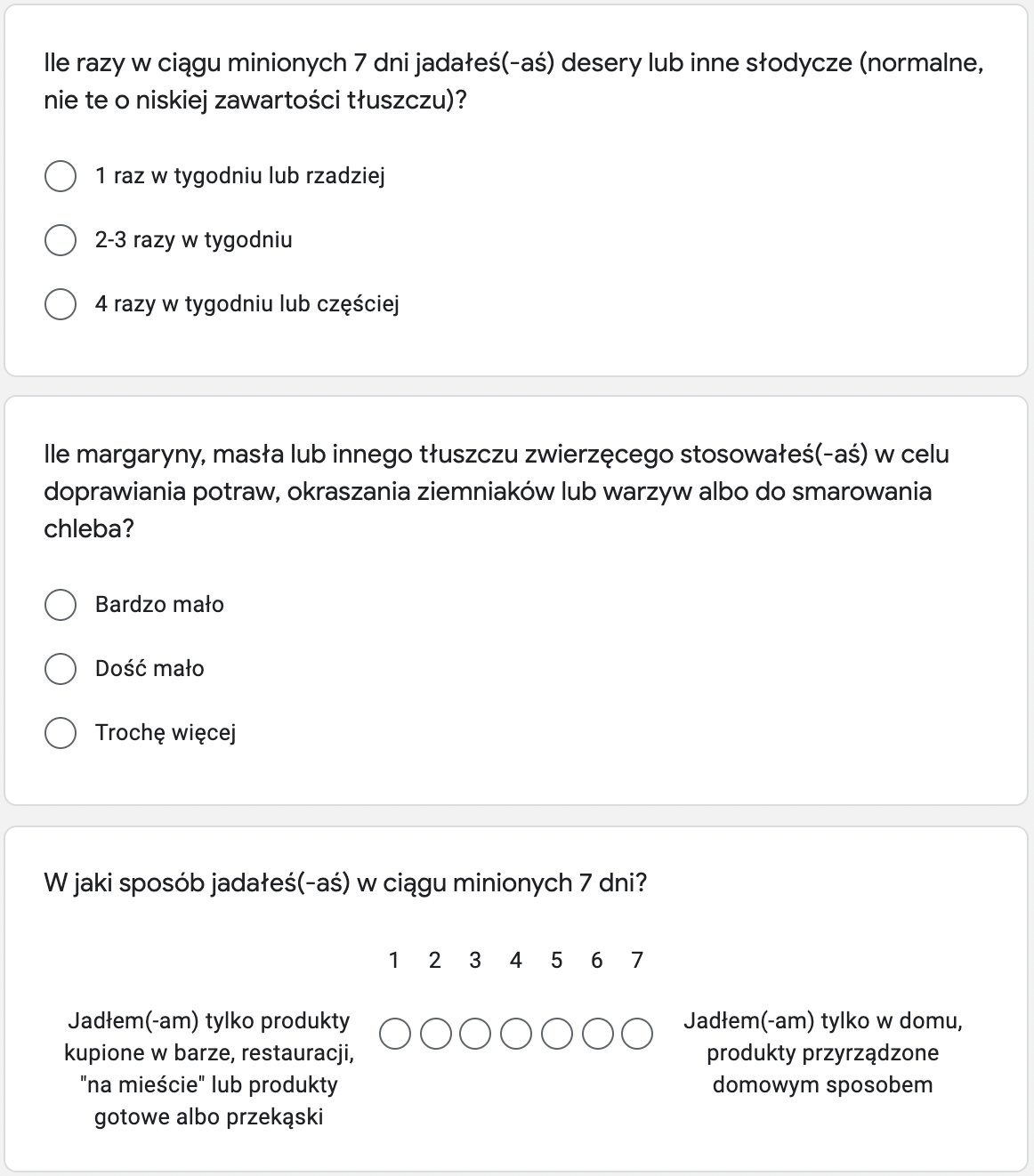


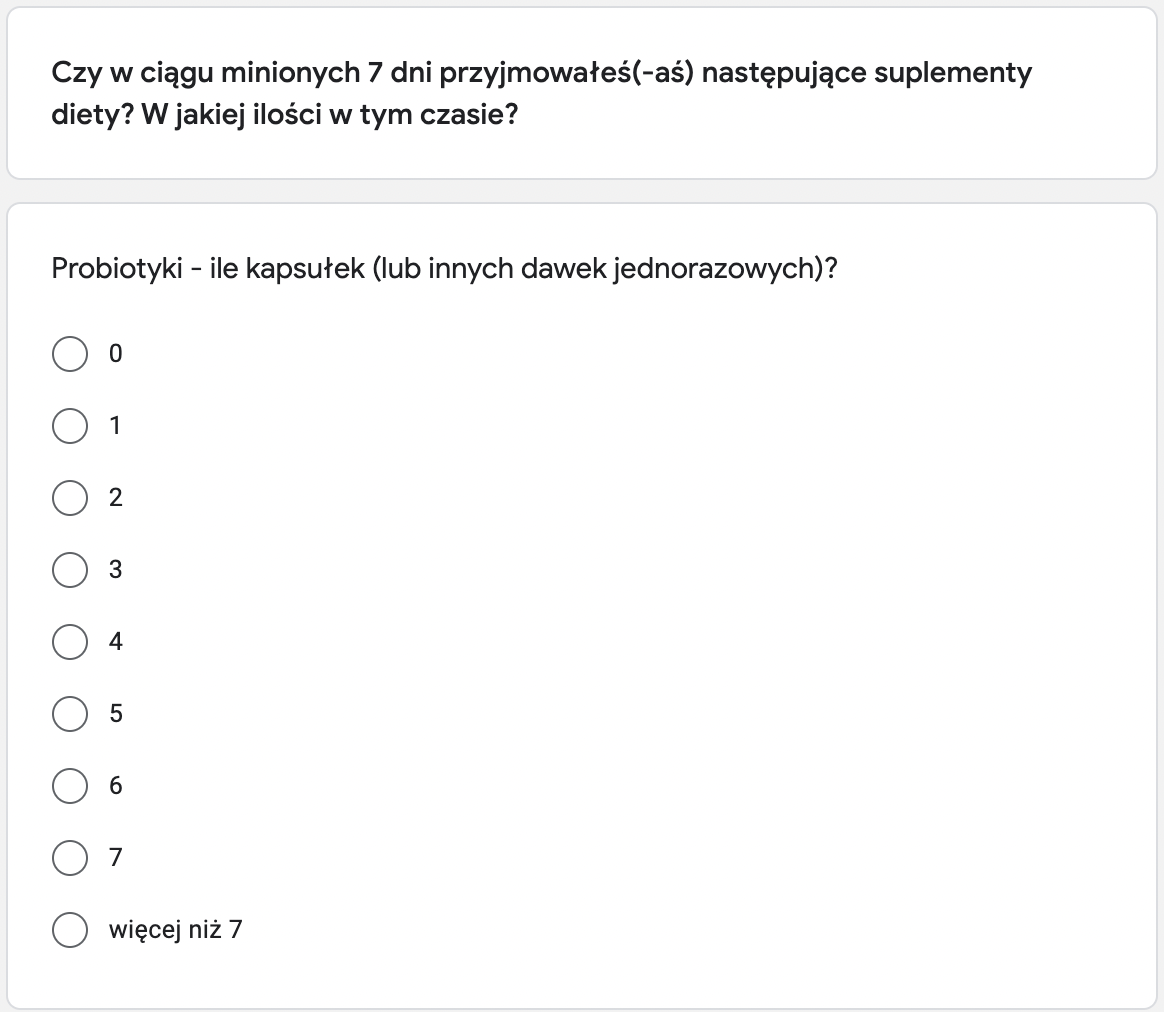


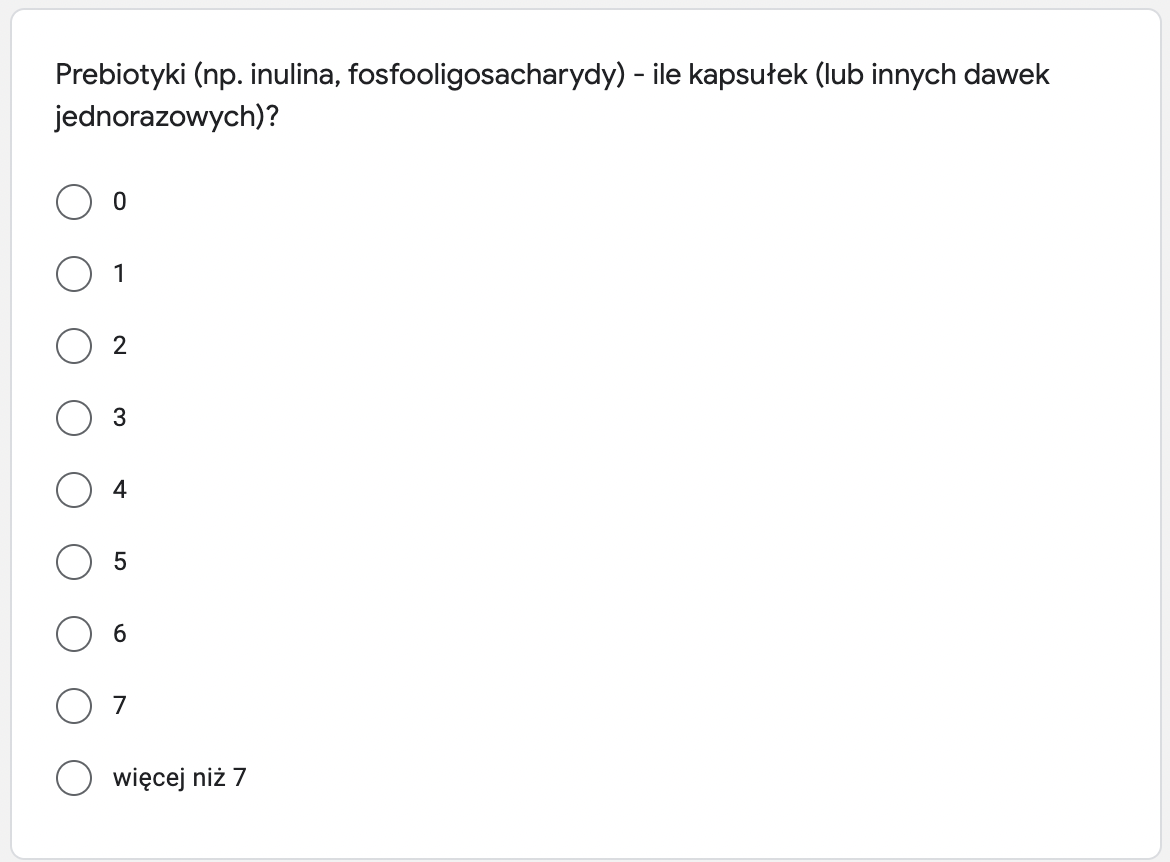


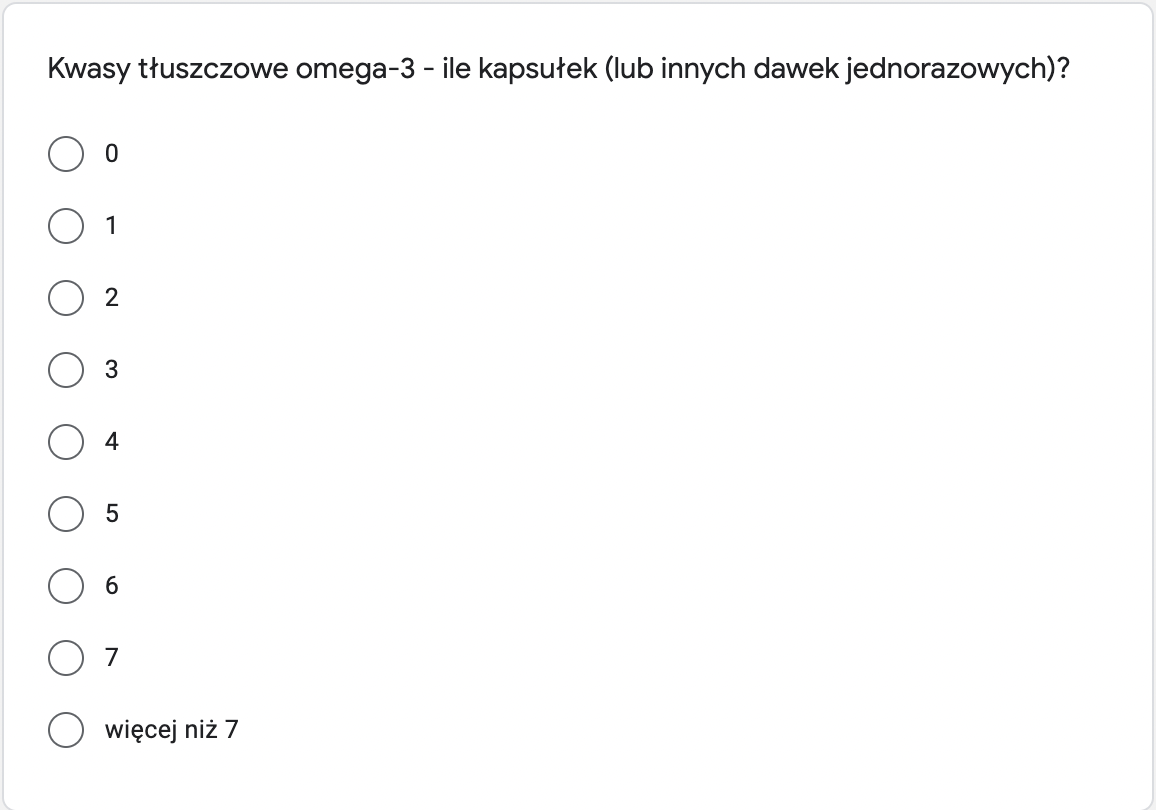


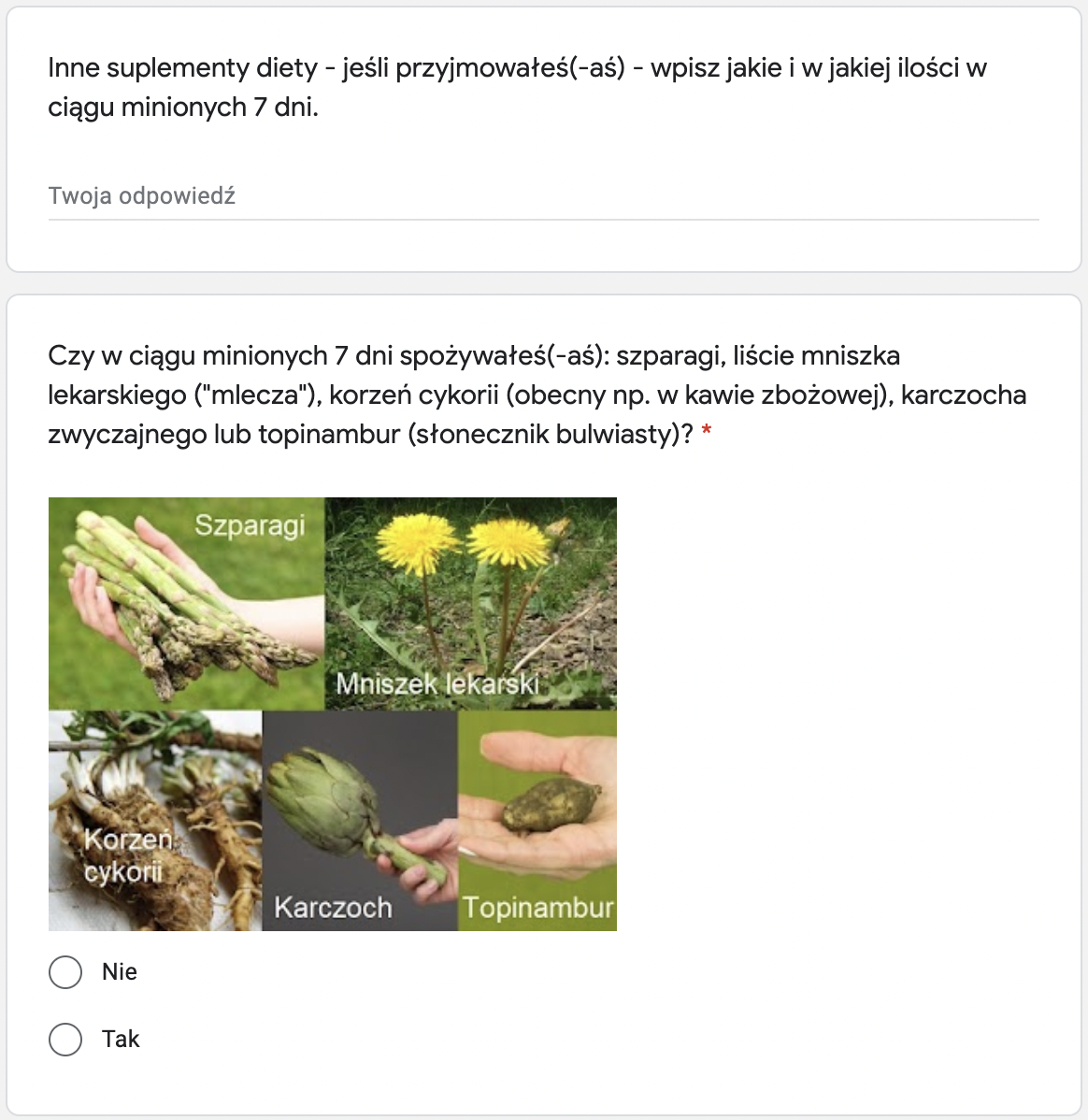


Conditional section (appeared only if a participant reported any consumption of the abovementioned vegetables):
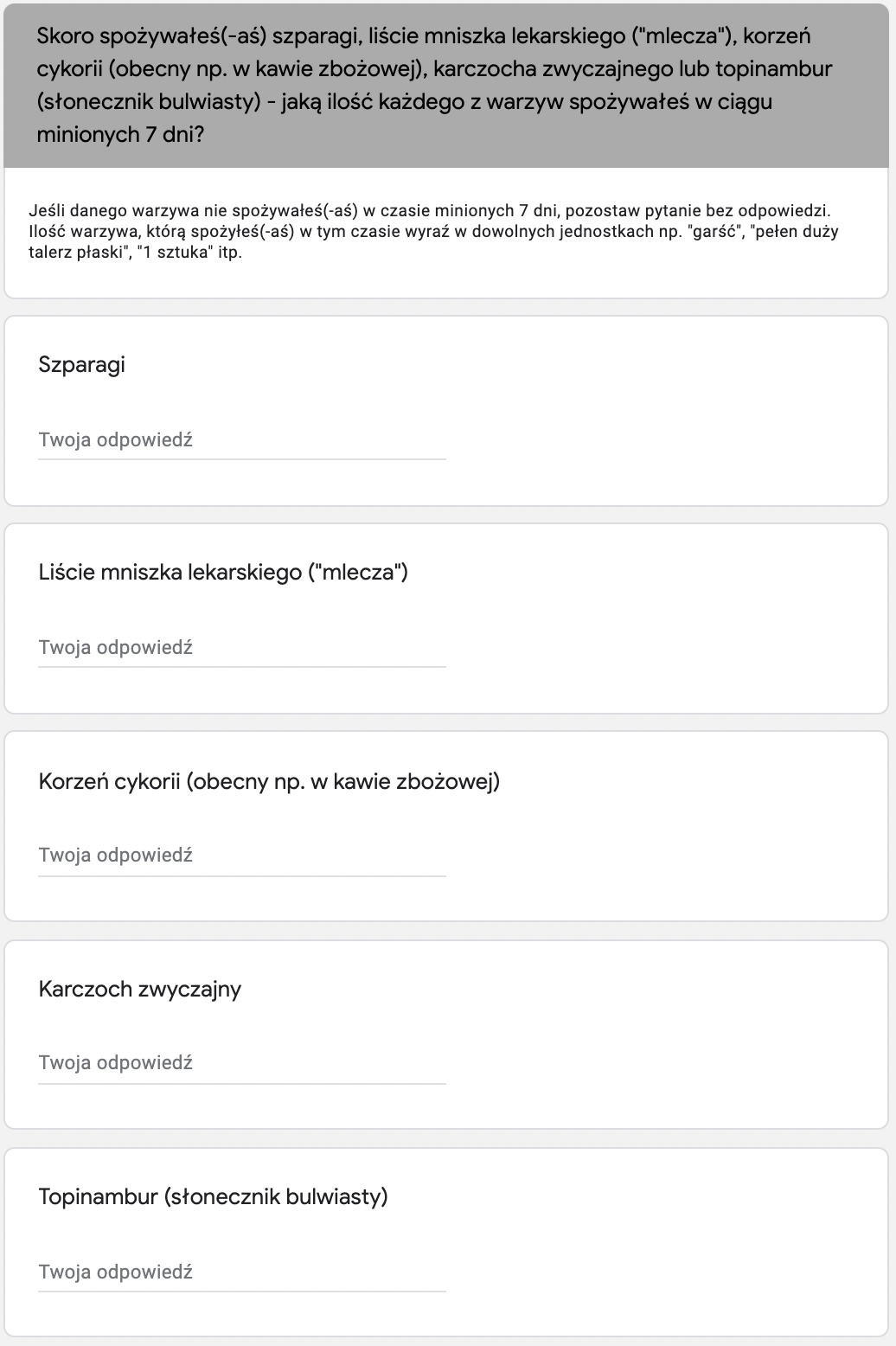


---------------- the end of the conditional section ----------------


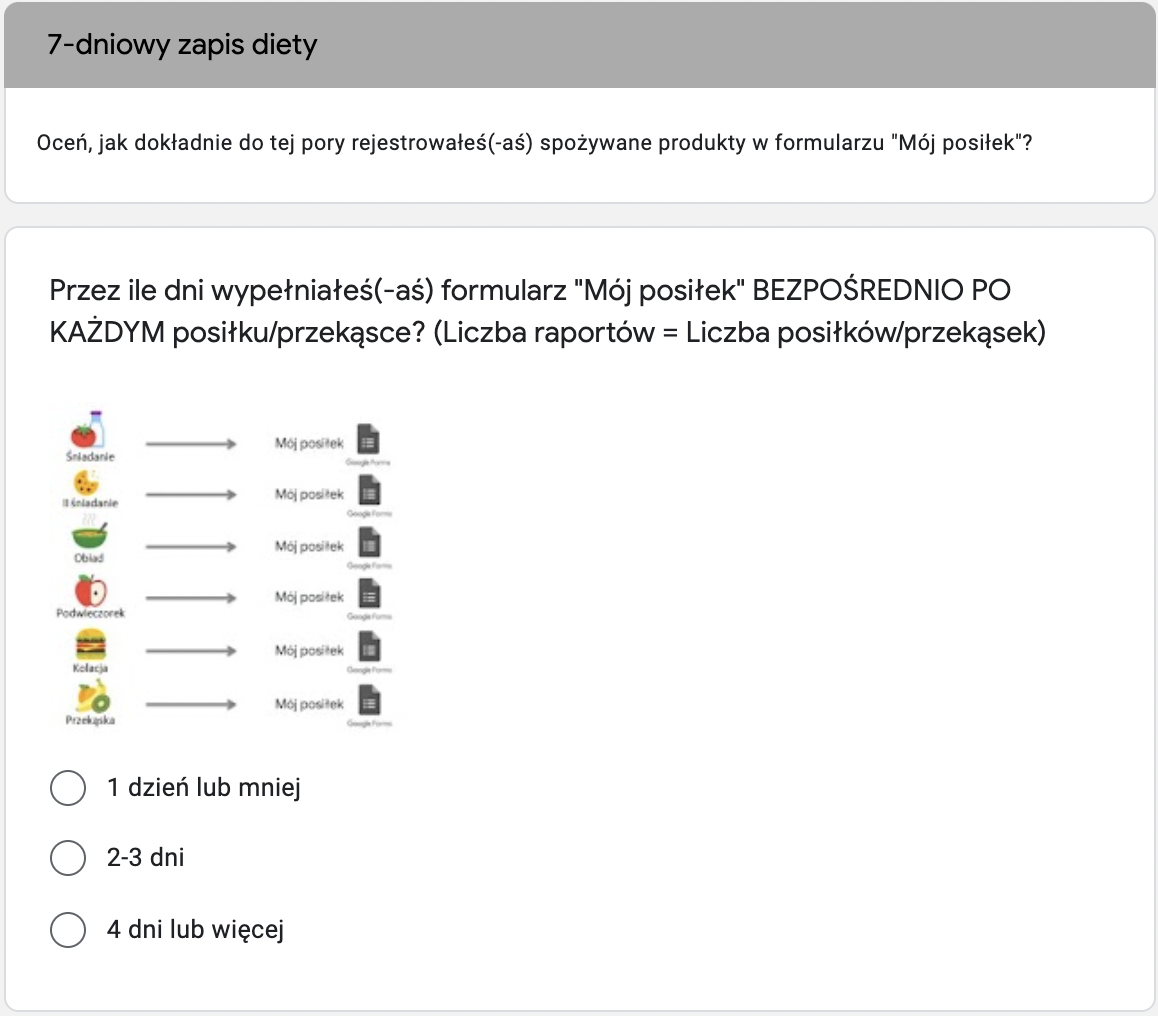


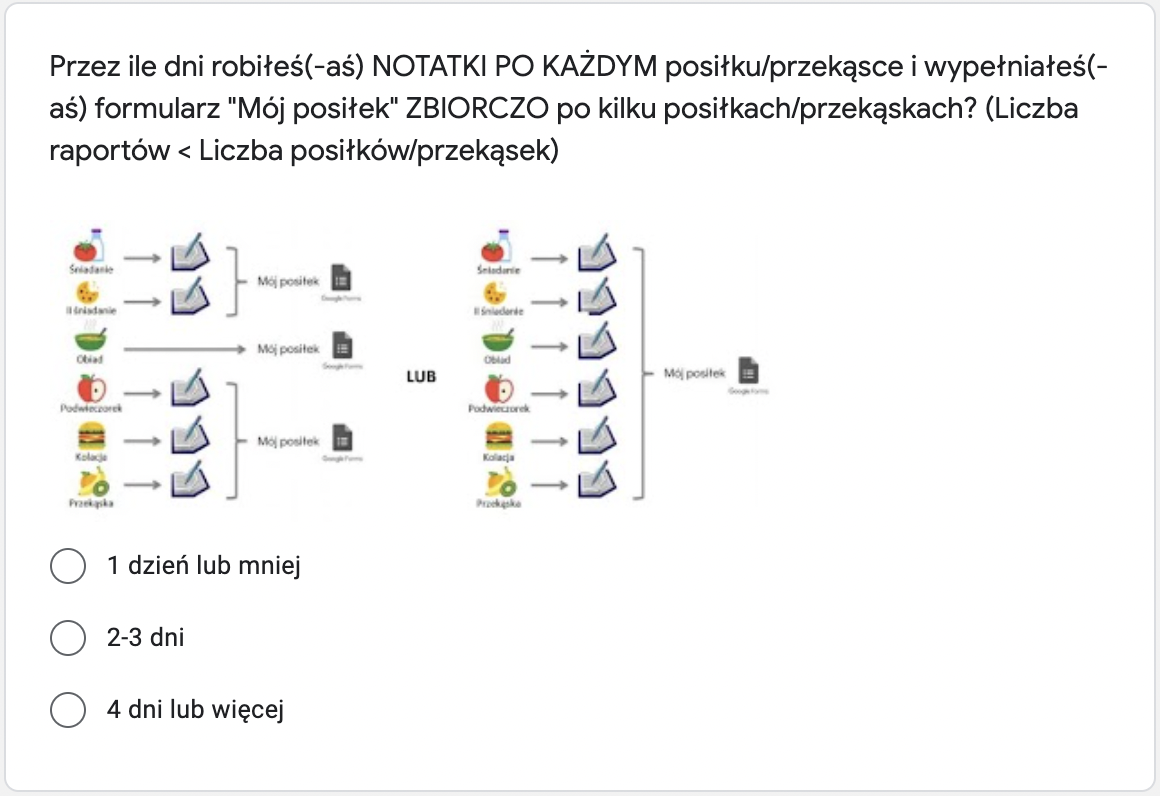


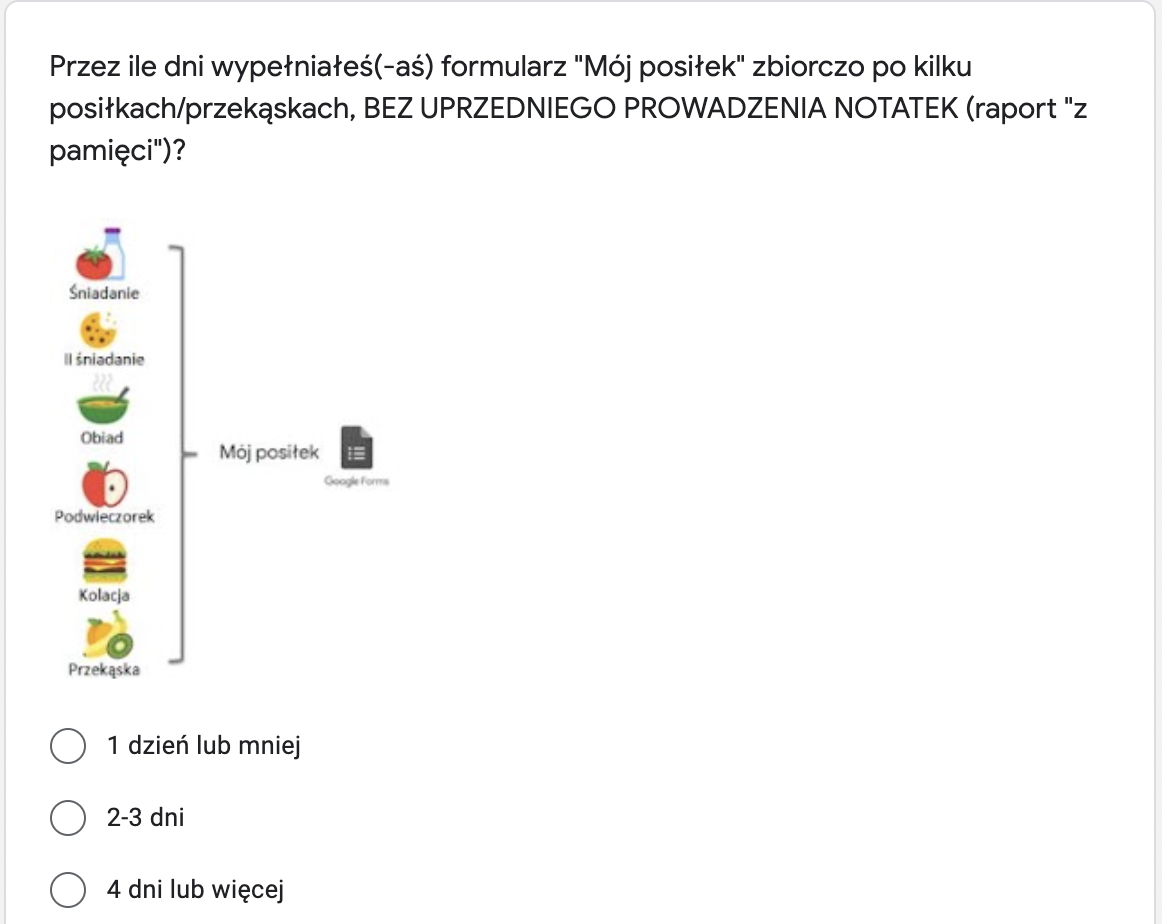


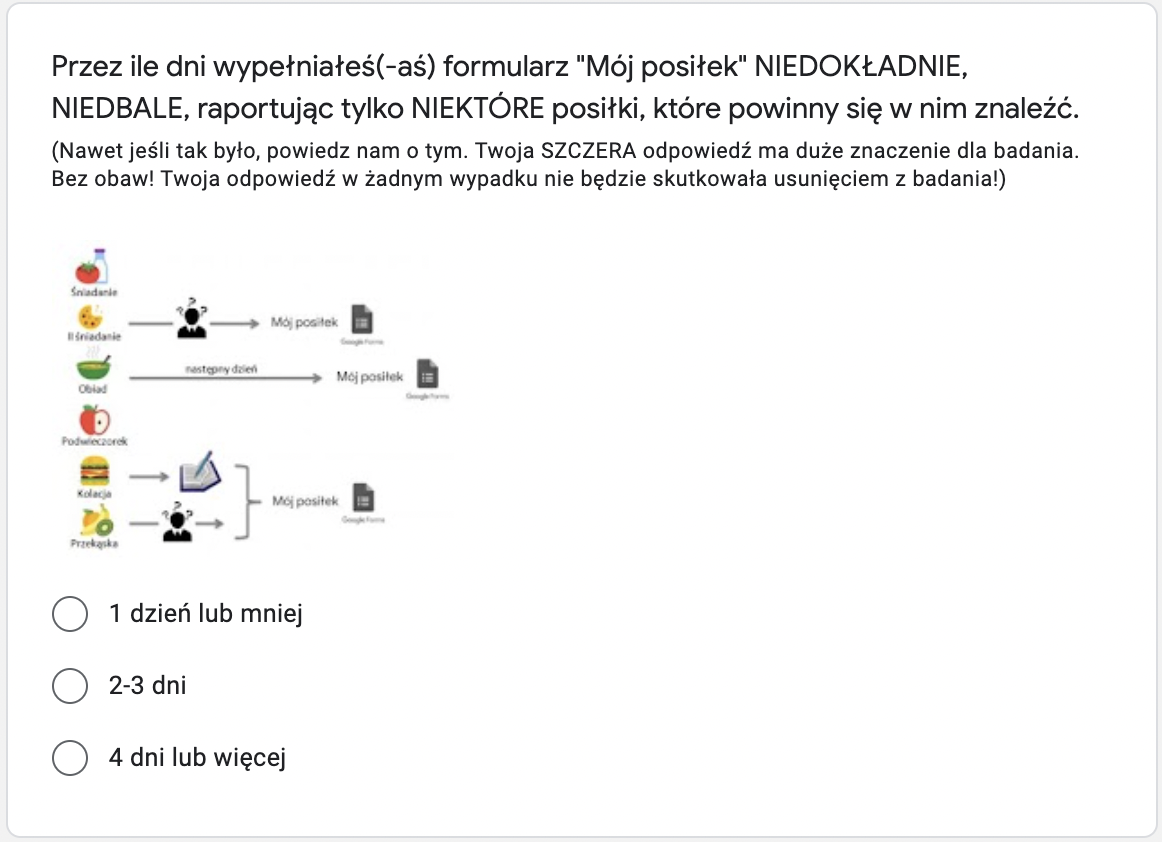


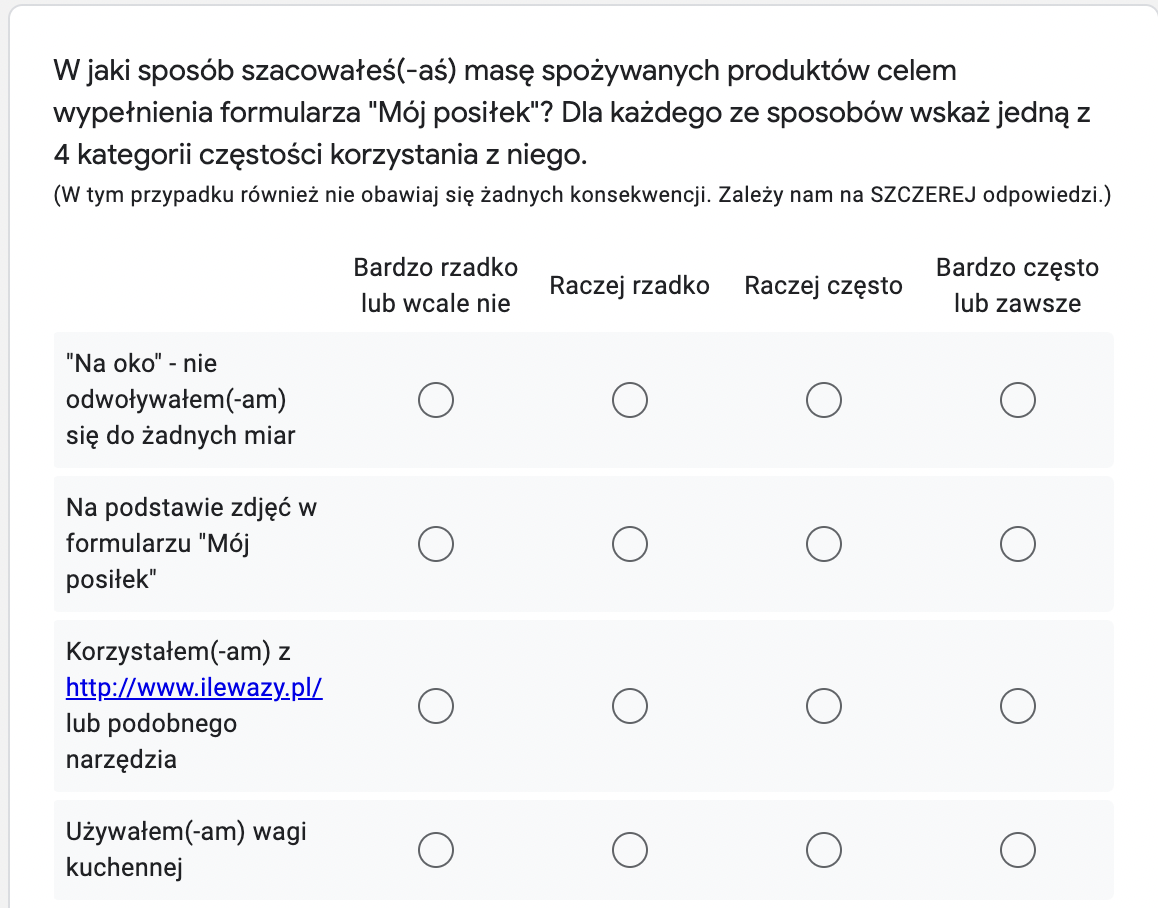


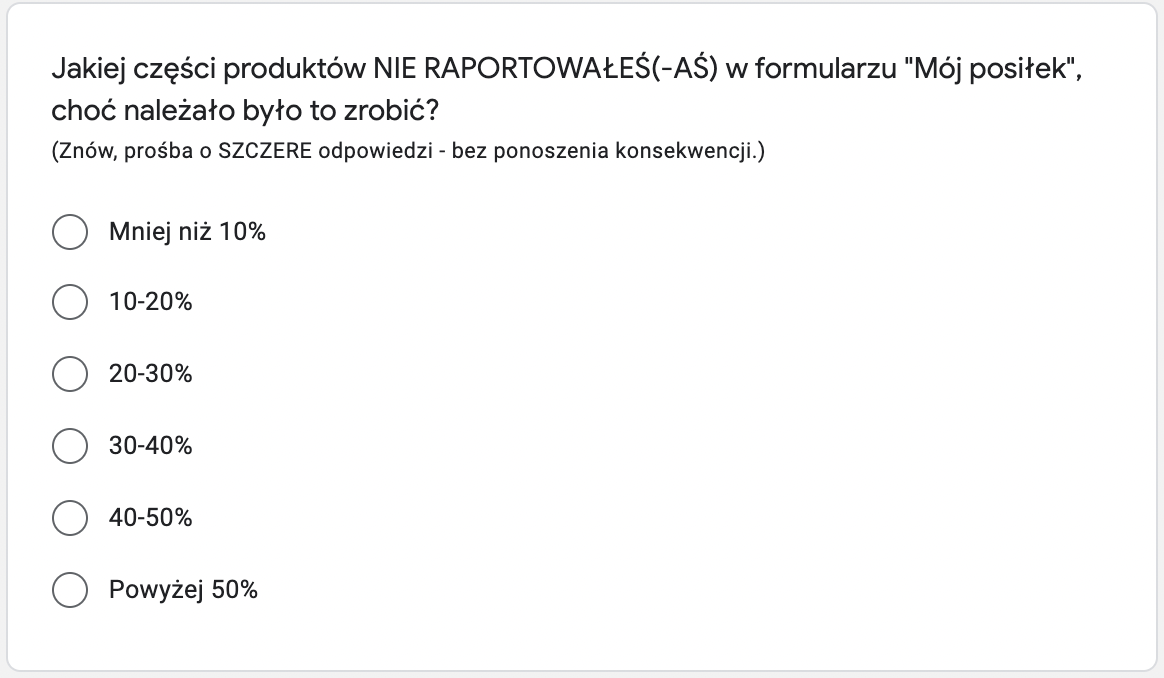


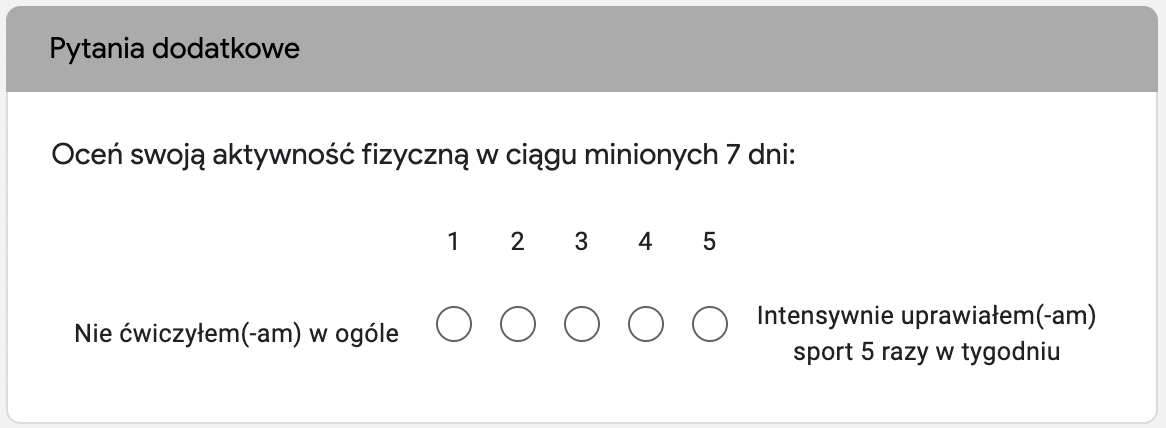


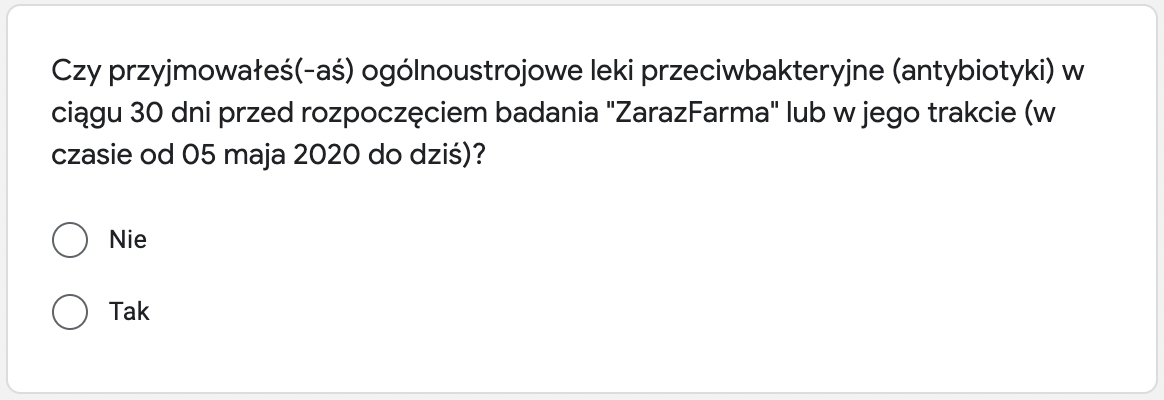


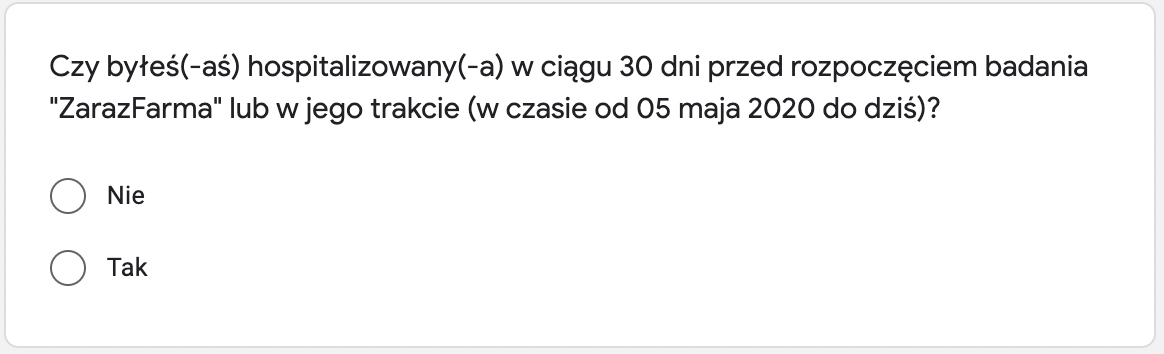


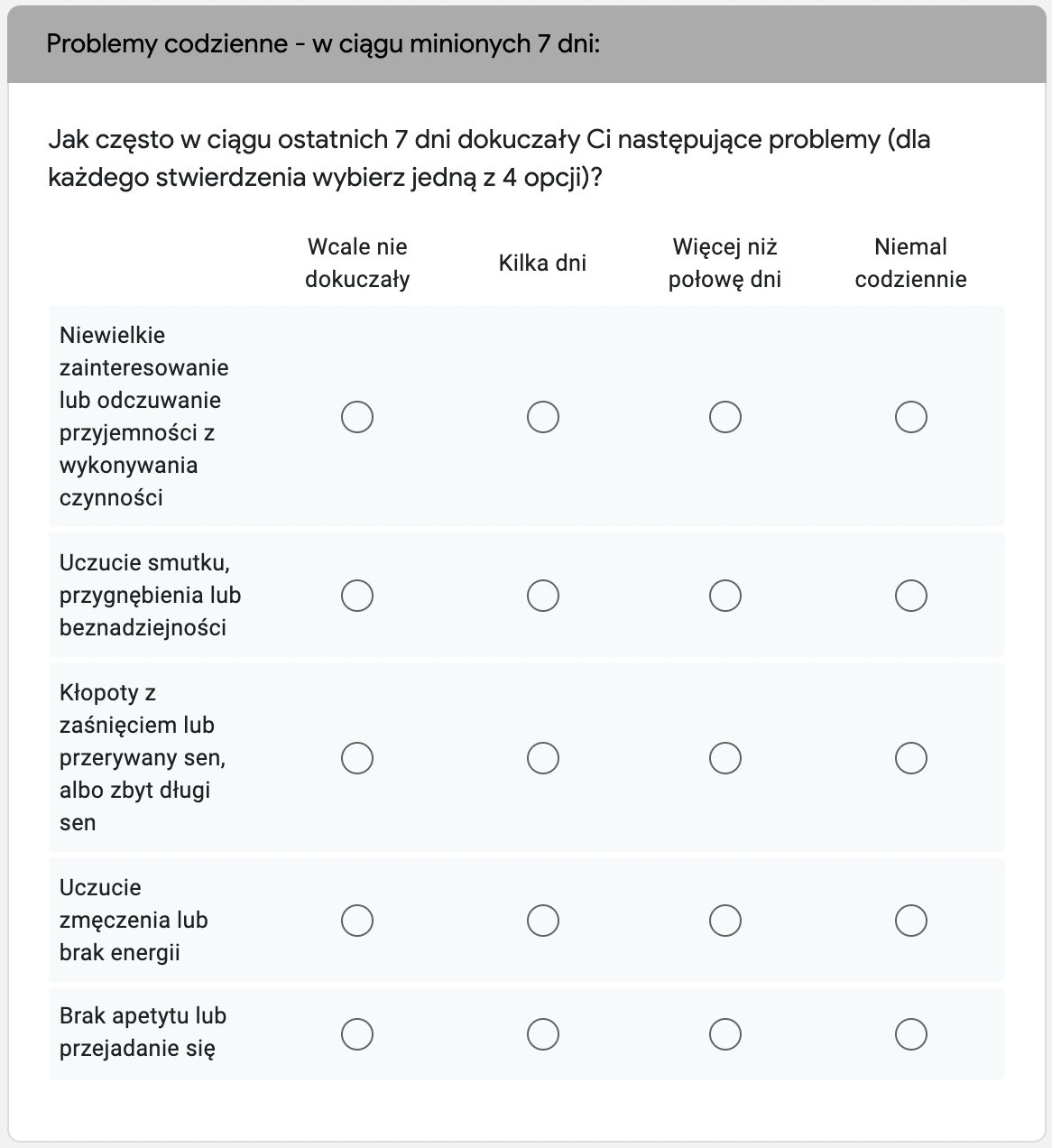


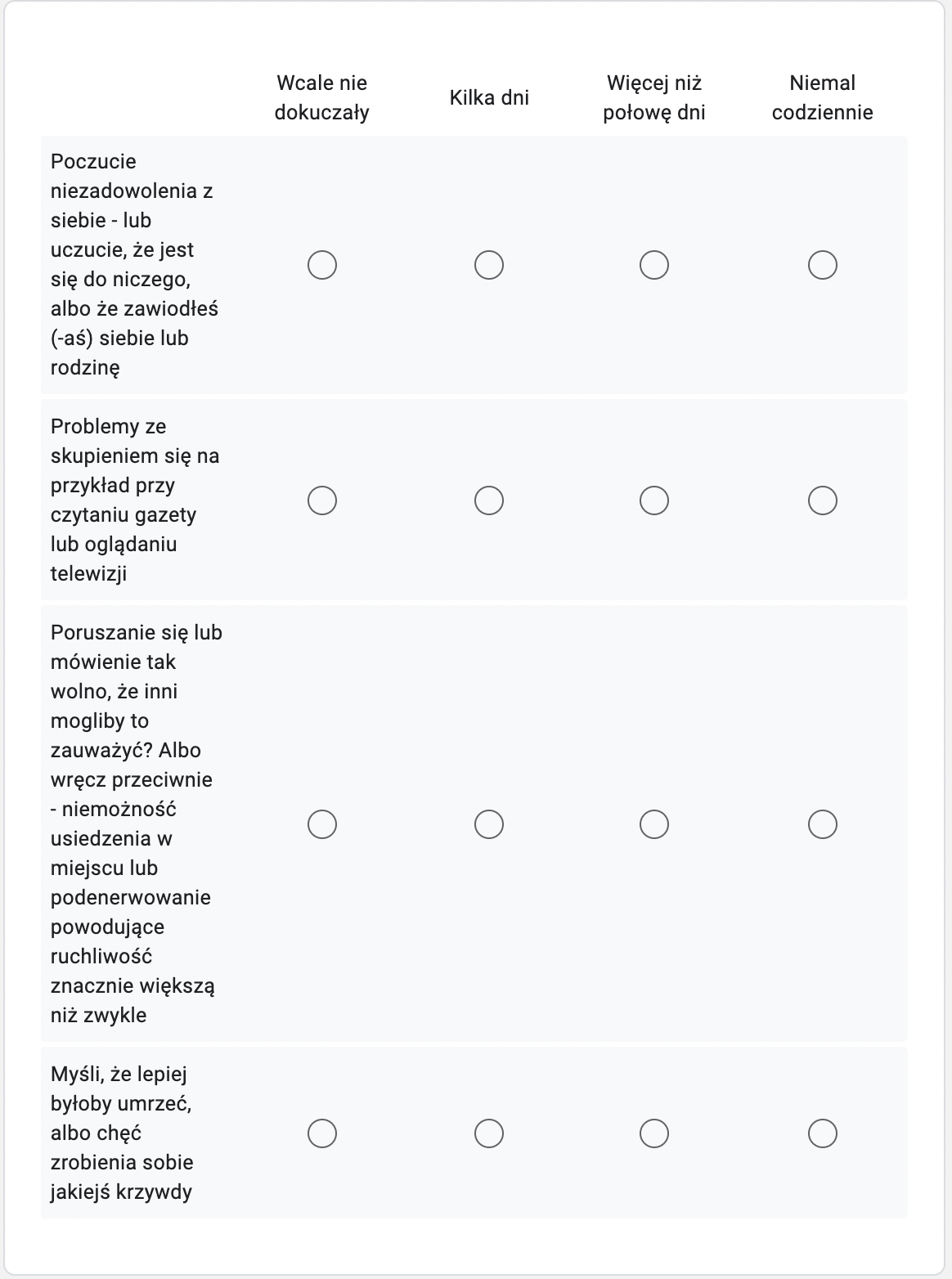


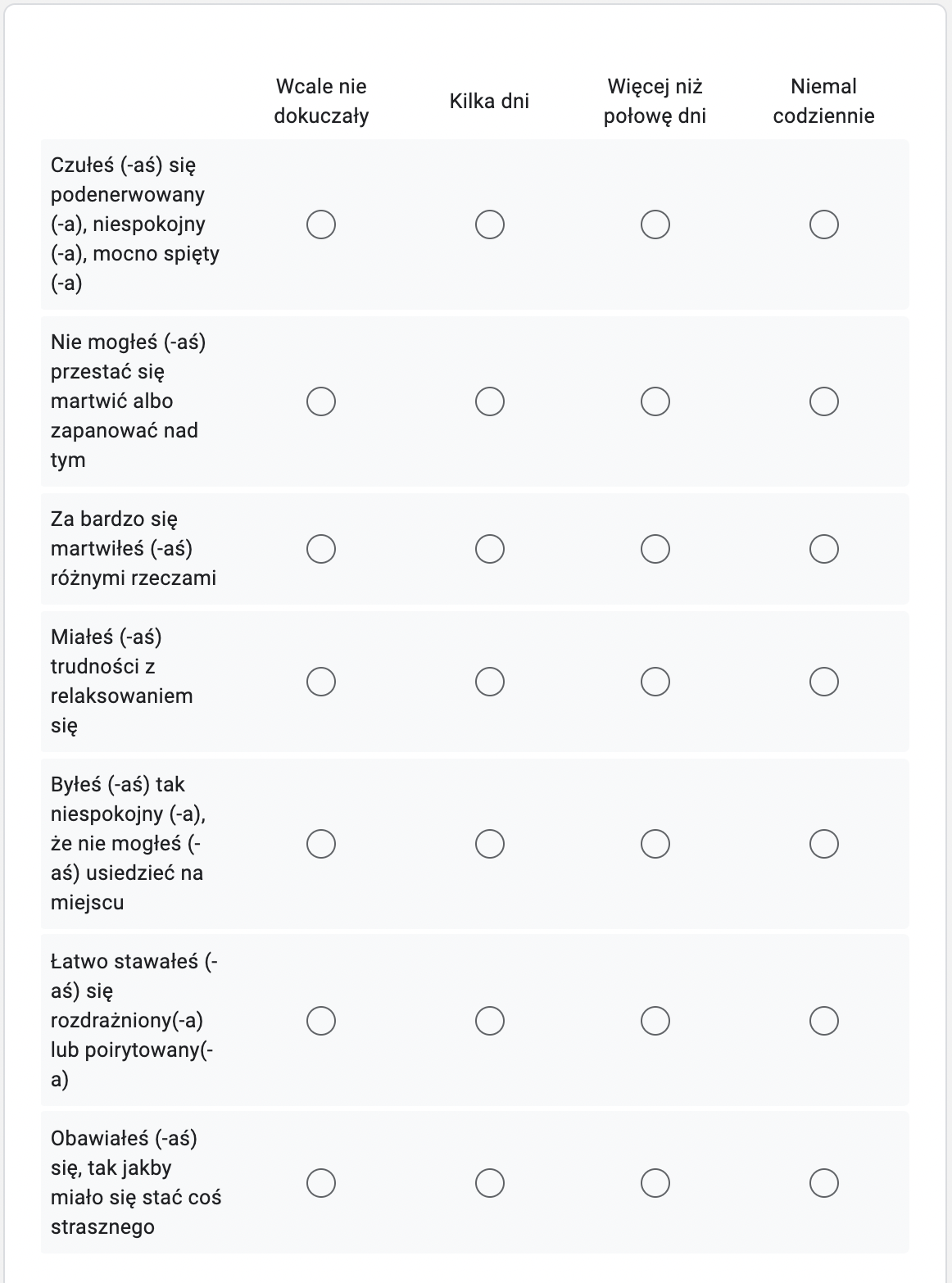


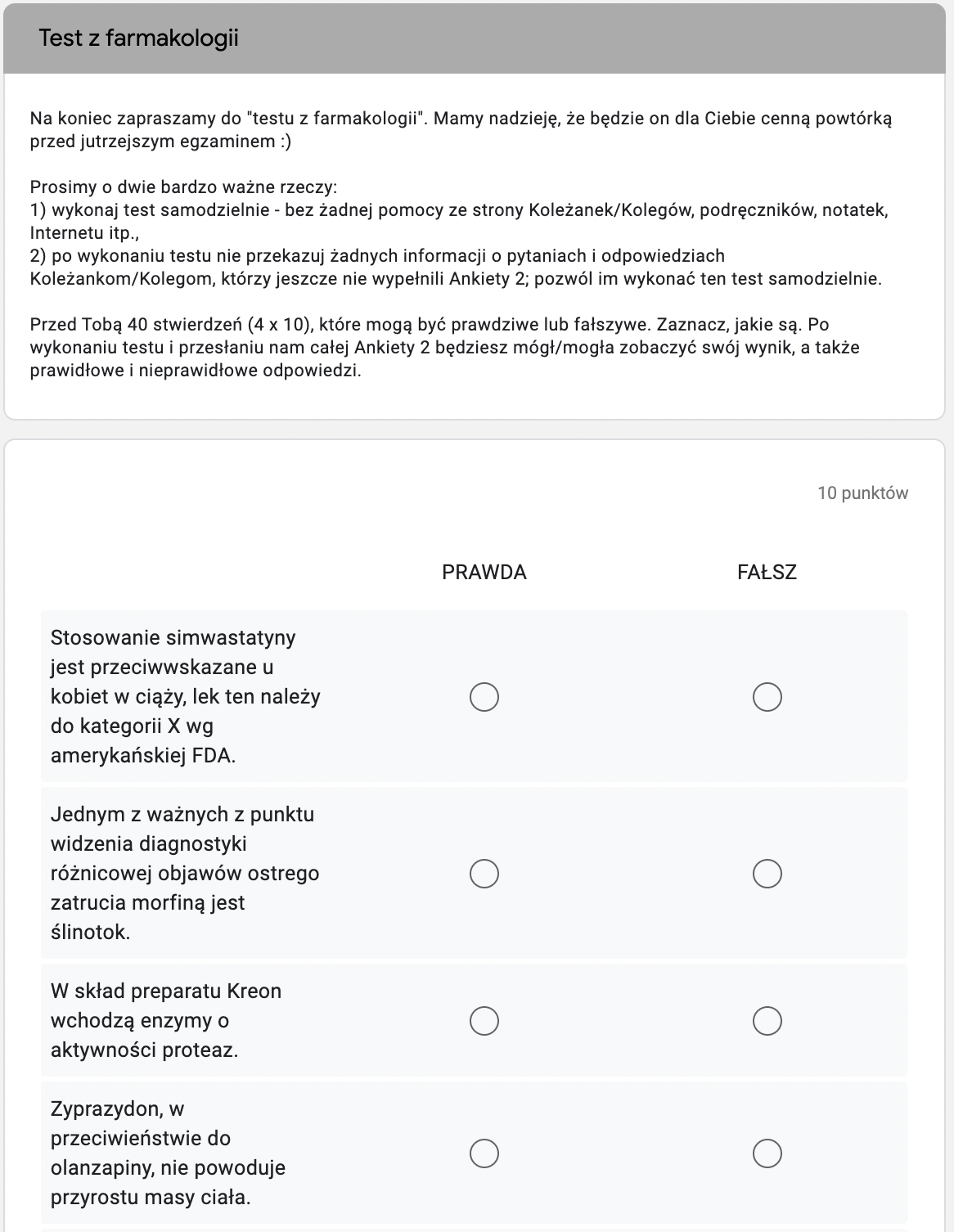


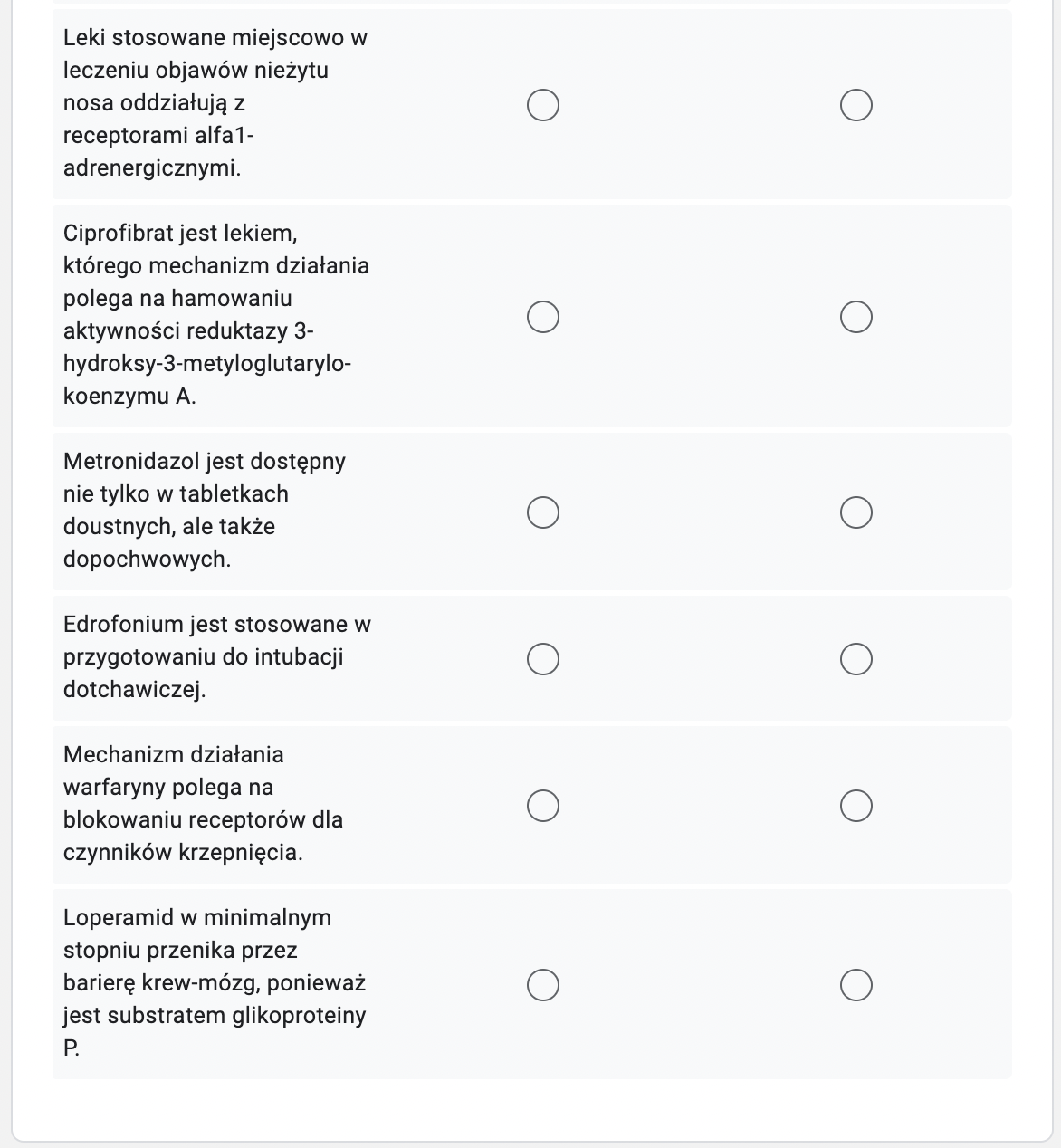


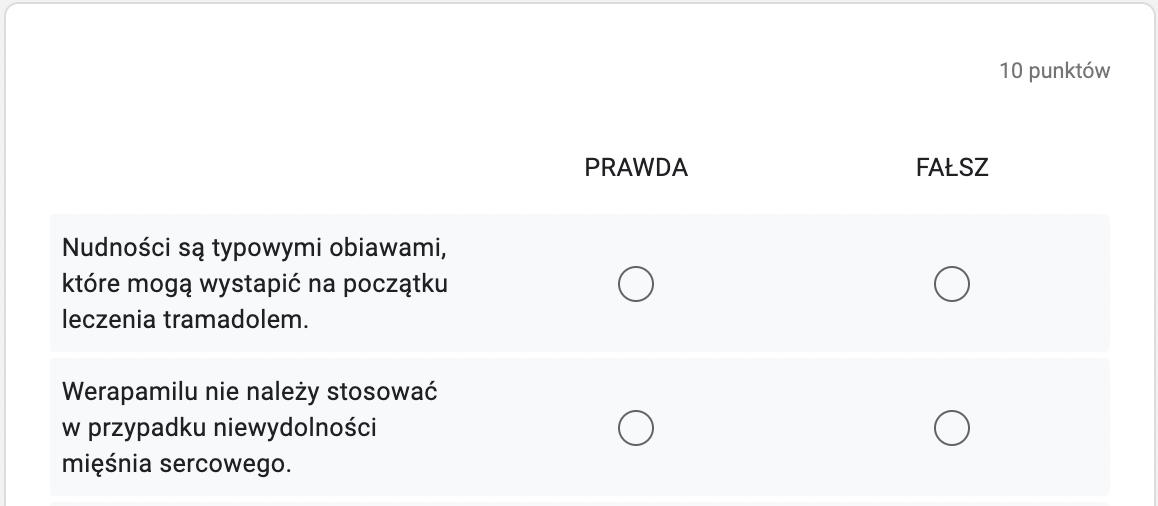


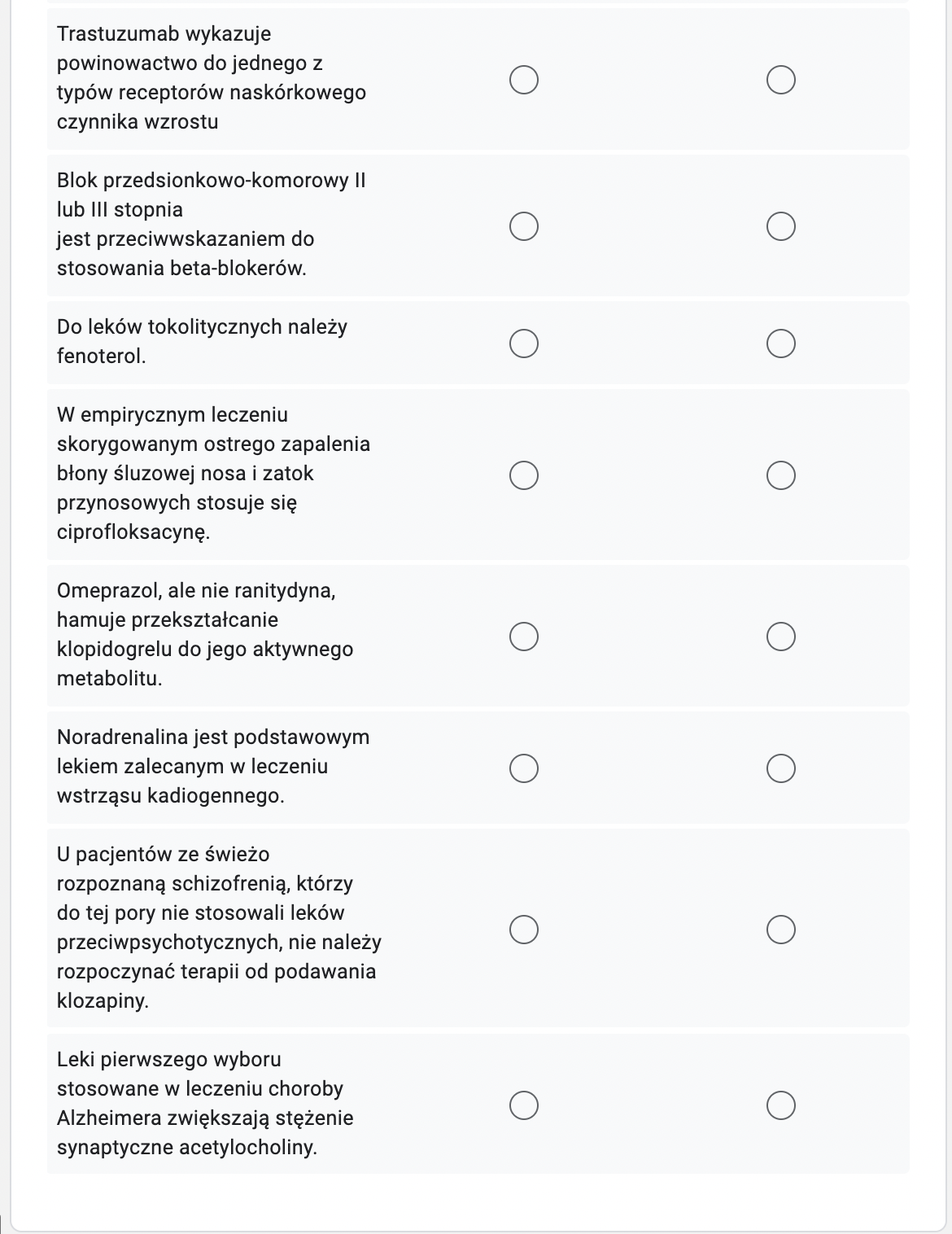


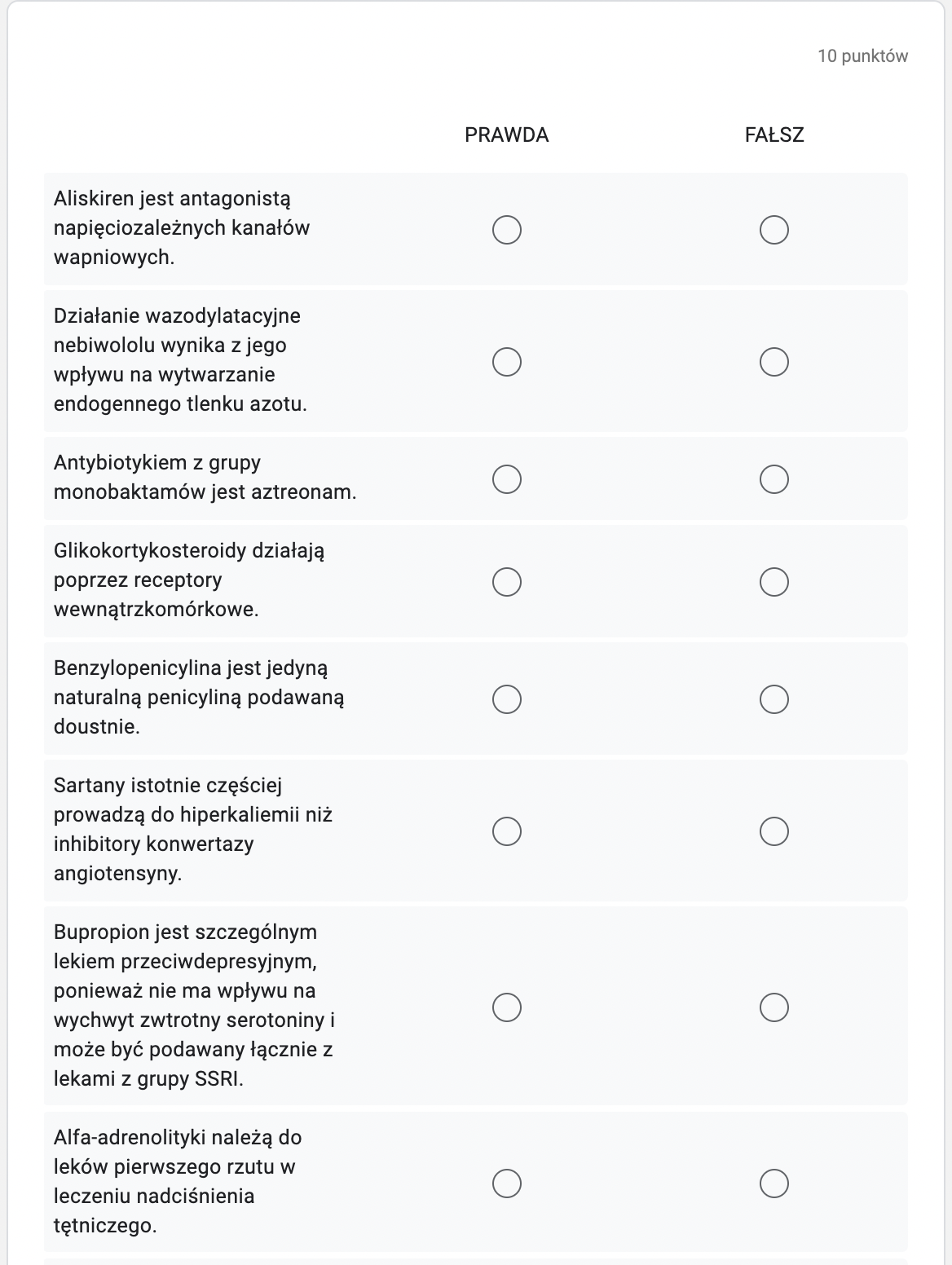

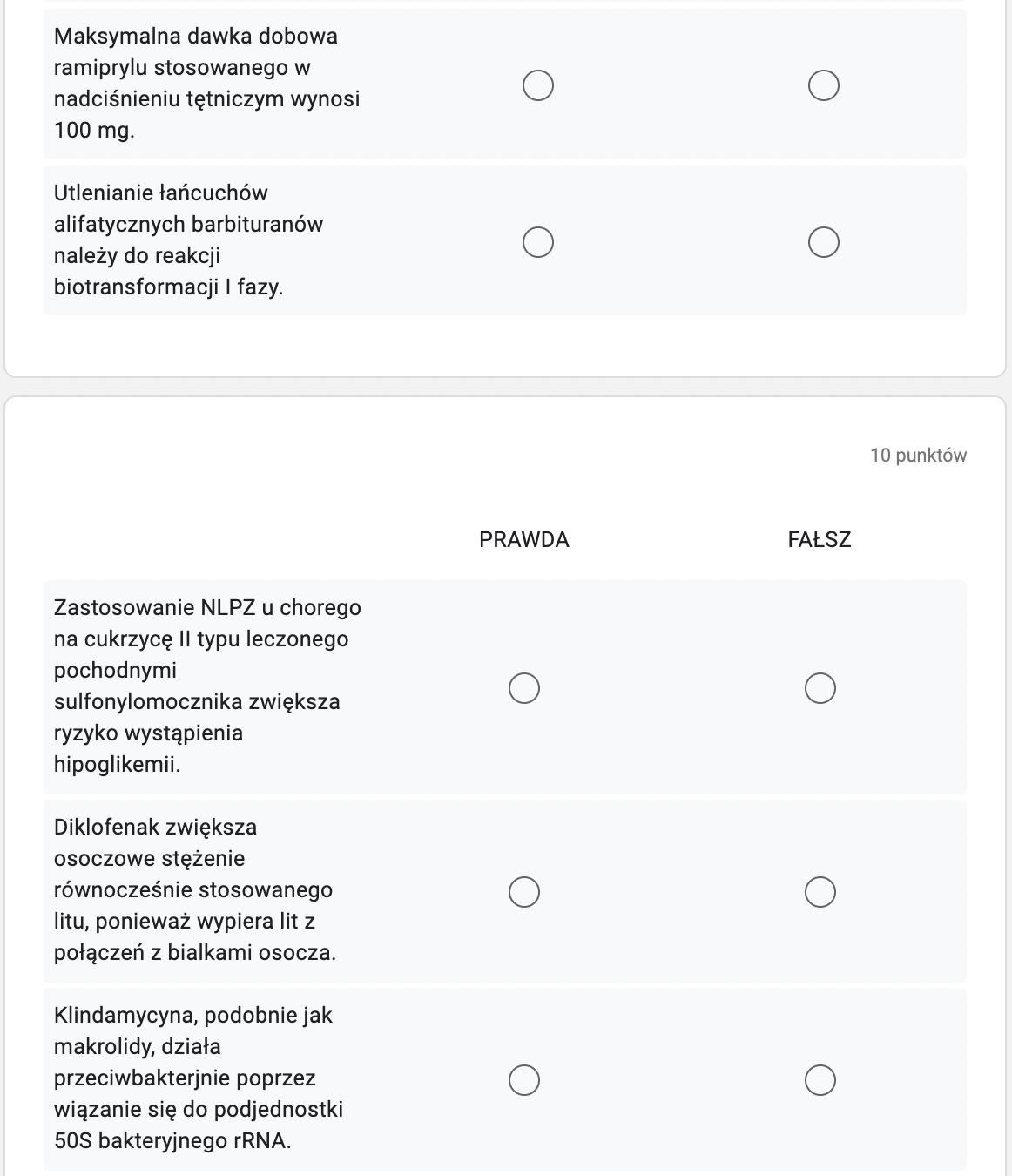


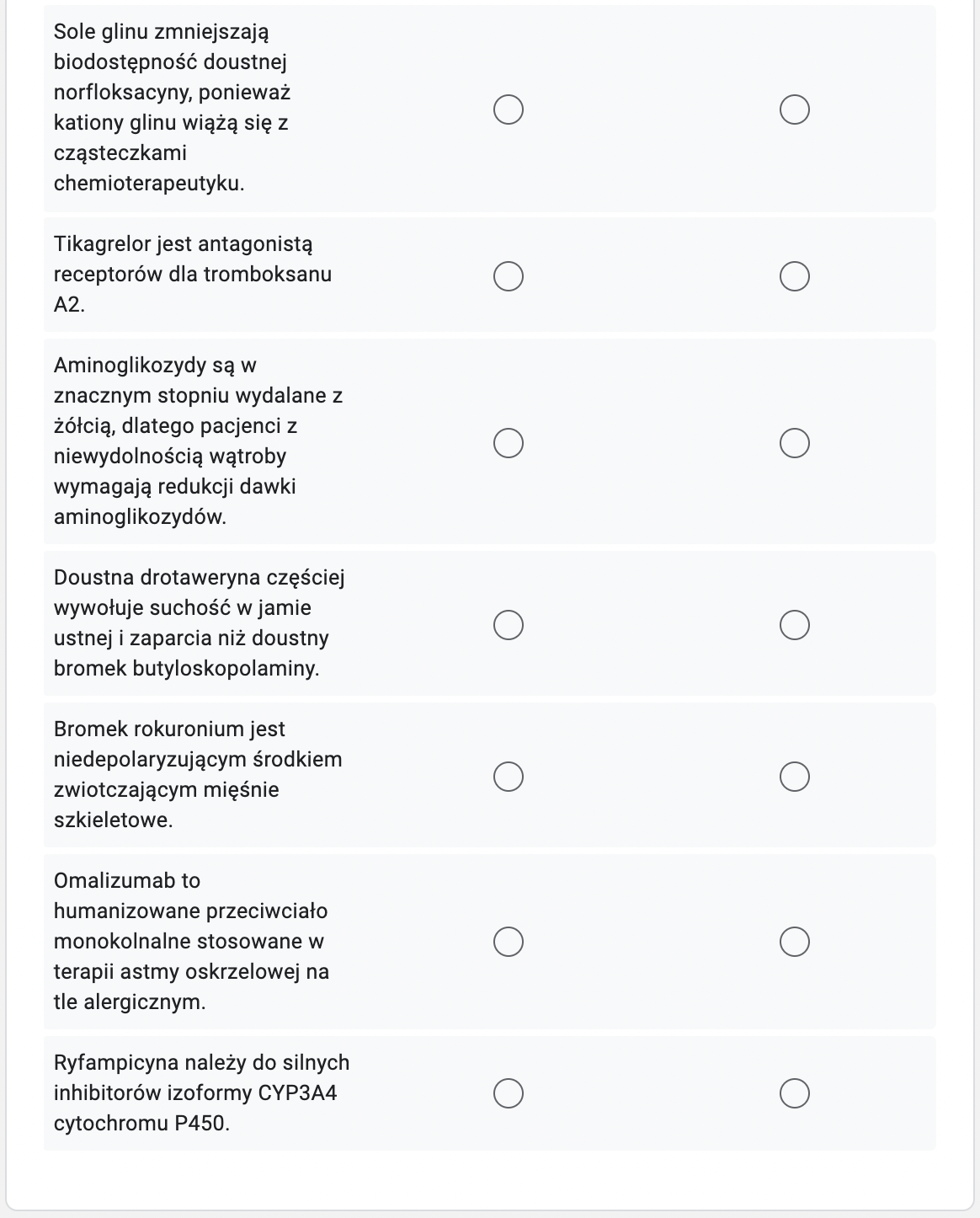


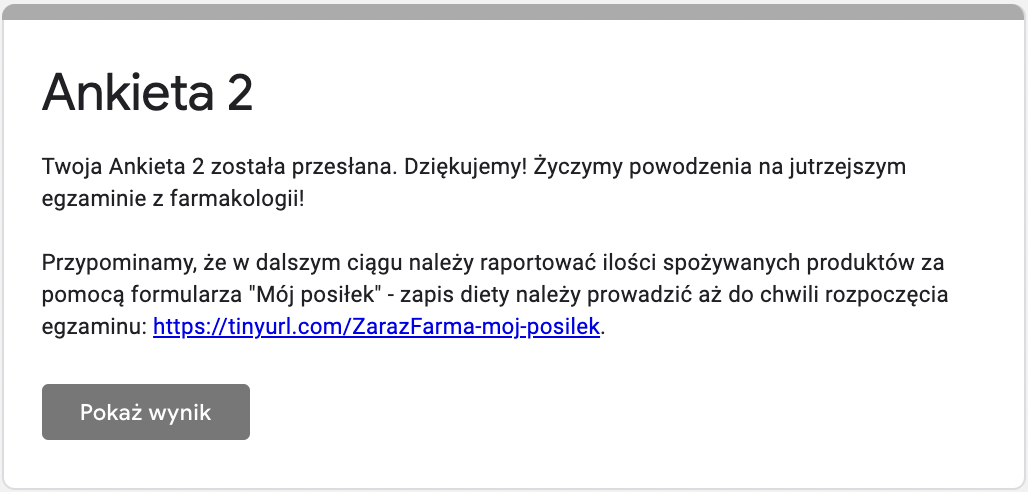


**English language translation of the original survey**

(The survey in the English language version is the free translations of the authors, it has not been validated for research use.)

**Survey 2**

Dear Students,

We welcome you to the next part of the study “Association between lifestyle factors and cognitive performance under stress” (ZarazFarma)”. In this survey we will ask you about the further information concerning your lifestyle, especially diet and everyday problems. We will also provide you with a pharmacology knowledge test, which – hopefully – will be a perfect recap one day before the final exam.

Filling out the survey will take around 10-15 minutes. Please feel free to start!

**Student ID number and faculty**

Please, write the number of your student ID.

Your faculty

- Faculty of Medicine
- Faculty of Military Medicine

**Diet**

Over the past seven days:

How many times did you eat fast food meals or snacks?

- Less than 1
- 1-2
- 3 or more

How many servings of fruit did you eat each day?

- 1 or less
- 2-3
- 4 or more

How many servings of vegetables did you eat each day?

- 2 or less
- 3-4
- 5 or more

How many glasses of carbonated drinks (such as cola, orangeade, energy drink) or glasses of sweet tea did you drink each day?

- less than 1
- around 1
- more than 1

How many times did you eat leguminous vegetables, chicken or fish?

- once or less
- 2-3
- 4 or more

How many times did you eat regular snack chips or crackers (not the low-fat kind)?

- once or less
- 2-3
- 4 or more

How much margarine, butter, or meat fat did you use to season vegetables or put on potatoes or bread?

- very little
- some
- a lot

What kind of meals have you eaten in the past seven days?

| I have eaten only street food or food bought in bars, restaurants or highly-processed food and snacks. | 1----2----3----4----5----6----7 | I have eaten only products cooked on my own at home. |
| --- | --- | --- |

**Did you take dietary supplements described below over the past seven days? If yes, in what amounts?**

Probiotics – how many capsules (or other single doses) did you take over the past seven days?

- 0
- 1
- 2
- 3
- 4
- 5
- 6
- 7
- more than 7

Prebiotics (such as insulin, fructooligosaccharides) – how many capsules (or other single doses) did you take over the past seven days?

- 0
- 1
- 2
- 3
- 4
- 5
- 6
- 7
- more than 7

Omega-3 fatty acids – how many capsules (or other single doses) did you take over the past seven days?

- 0
- 1
- 2
- 3
- 4
- 5
- 6
- 7
- more than 7

Other dietary supplements – if you took them – please, write the name of them and how much you took over the past seven days.

Did you consume asparagus, dandelion leaves, chicory roots, globe artichokes or Jerusalem artichokes over the past seven days?

- No
- Yes

*Conditional section (appeared only if a participant reported any consumption of the abovementioned vegetables):*

You marked that you had eaten asparagus, dandelion leaves, chicory roots (which is present for example in chicory coffee), globe artichokes or Jerusalem artichokes. How much of each did you eat over the past seven days?

If you did not eat some of the vegetables over the past seven days, leave a question without any answer. Please, describe the amount of the vegetable you ate in any units, for example: “a handful”, “a full big plate”, “one piece” etc.

Asparagus…

Dandelion leaves…

Chicory root (which is present for example in chicory coffee)…

Globe artichokes…

Jerusalem artichokes…

--- *the end of the conditional section* ---

**Seven-day-long diet record**

Please, assess how accurately you recorded the food you ate in the form “My meal”.

For how many days did you fill out the form “My meal” DIRECTLY AFTER EVERY meal/snack? (The number of records = the number of meals/snacks)

- 1 day or less
- 2-3 days
- 4 days or more

For how many days did you make NOTES AFTER EVERY meal/snack and fill out the form “My meal” COLLECTIVELY after a few meals/snacks? (The number of records < the number of meals/snacks)

- 1 day or less
- 2-3 days
- 4 days or more

For how many days did you fill out the form “My meal” collectively after a few meals/snacks, WITHOUT MAKING NOTES BEFORE (records “from memory”)?

- 1 day or less
- 2-3 days
- 4 days or more

For how many days did you fill out the form “My meal” INACCURATELY AND ROUGHLY, so you recorded only SOME meals, which should have been recorded?

(Even if you did, tell us about it. Your HONEST answer matters a lot for the research. Do not worry! Your answer will not cause you to be rejected from the research!)

- 1 day or less
- 2-3 days
- 4 days or more

How did you assess the mass of the food you ate while filling out the form “My meal”? Choose one option for each statement.

(In this case also do not worry that it would have negative consequences. Your HONEST answer matters a lot for us.)

|  | Very rarely or never | Rarely | Often | Very often or always |
| --- | --- | --- | --- | --- |
| I did not use any special measures, I assessed it approximately |  |  |  |  |
| I based on the photos in the form “My meal” |  |  |  |  |
| I based it on <http://www.ilewazy.pl> or a similar tool |  |  |  |  |
| I used a kitchen scales |  |  |  |  |

What part of the food you ate you did not record in the form “My meal”, even though it should have been done?

(Again, please answer honestly without suffering consequences.)

- Less than 10%
- 10-20%
- 20-30%
- 30-40%
- 40-50%
- More than 50%

**Additional questions**

Assess your physical activity over the past seven days.

| I did not exercise at all. | 1-----2-----3-----4-----5 | I did sports intensively five times a week. |
| --- | --- | --- |

Did you take antibiotics within the last 30 days before participating in the research “ZarazFarma” or during the time of the research?

- No
- Yes

Were you hospitalized within the last 30 days before participating in the research “ZarazFarma” or during the time of the research?

- No
- Yes

**Everyday problems over the past seven days**

Over the last seven days, how often have you been bothered by the following problems?

(Please, for each statement choose one out of four options.)

|  | Not at all | Several days | More than half the days | Nearly every day |
| --- | --- | --- | --- | --- |
| Little interest or pleasure in doing things |  |  |  |  |
| Feeling down, depressed,  or hopeless |  |  |  |  |
| Trouble falling or staying asleep, or sleeping too much |  |  |  |  |
| Feeling tired or having little energy |  |  |  |  |
| Poor appetite or overeating |  |  |  |  |
| Feeling bad about yourself -  or that you are a failure or have let yourself or your family down |  |  |  |  |
| Trouble concentrating on things, such as reading the newspaper  or watching television |  |  |  |  |
| Moving or speaking so slowly that other people could have noticed?  Or the opposite - being so fidgety or restless that you have been moving around a lot more than usual |  |  |  |  |
| Thoughts that you would be better off dead, or of hurting yourself  in some way |  |  |  |  |
| Feeling nervous, anxious,  or on edge |  |  |  |  |
| Not being able to stop or control worrying |  |  |  |  |
| Worrying too much about different things |  |  |  |  |
| Trouble relaxing |  |  |  |  |
| Being so restless that it is hard to sit still |  |  |  |  |
| Becoming easily annoyed  or irritable |  |  |  |  |
| Feeling afraid, as if something awful might happen |  |  |  |  |

**Pharmacology knowledge test**

At the end, we welcome you to the “pharmacology knowledge test”. We hope that it will be a perfect recap before tomorrow’s exam :)

We ask you about two very important things:

1. take a test on your own, which means without any help from your Colleagues, books, notes, the Internet etc.
2. after taking a test, do not share any information about questions and answers with your Colleagues, who do not fill out The Second Survey; let them take this test on their own.

In front of you there are 40 statements, which might be true or false. Rate them. After finishing the test and sending us the whole Second Survey, you will receive your result with correct and incorrect answers marked.

|  | True | False |
| --- | --- | --- |
| Taking simvastatin is contraindicated among pregnant women; this drug is rated as an X Category drug by the U.S. FDA. |  |  |
| In view of differential diagnosis, one of the most important symptoms of acute morphine overdose is salivation. |  |  |
| “Kreon” is a medication which contains protease enzymes. |  |  |
| Ziprasidone, unlike olanzapine, does not cause increase in body mass. |  |  |
| Topical medications against symptoms of rhinorrhea impact alpha1-adrenergic receptors. |  |  |
| Ciprofibrate is a drug, whose mechanism of action bases on inhibition of the enzyme 3-hydroxy-3-methylglutaryl coenzyme A (HMG-CoA) reductase. |  |  |
| Metronidazole is available not only in oral tablets, but also in vaginal forms. |  |  |
| Edrophonium is used in preparation for tracheal intubation. |  |  |
| Warfarin’s mechanism of action is based on blocking receptors for coagulation factors. |  |  |
| Loperamide only minimally crosses the blood-brain barrier, because it is a substrate for P-glycoprotein. |  |  |
| Nausea is a typical symptom which might occur at the beginning of using tramadol. |  |  |
| Verapamil is contraindicated in the case of heart failure. |  |  |
| Trastuzumab has affinity to one of the types of the epidermal growth factor receptors. |  |  |
| Second-degree or third-degree atrioventricular block is a contraindication to using beta-blockers. |  |  |
| Fenoterol belongs to tocolytics agents. |  |  |
| Ciprofloxacin is used in corrected empirical treatment of acute rhinitis and sinusitis. |  |  |
| Omeprazole, unlike ranitidine, suppresses transformation of clopidogrel to its active metabolite. |  |  |
| Norepinephrine is a first line drug used in cardiogenic shock. |  |  |
| Among patients recently diagnosed with schizophrenia, who have not been treated with antipsychotics agents so far, pharmacotherapy should not be started with clozapine. |  |  |
| Drugs used as an initial treatment for Alzheimer’s disease increase the synaptic concentration of acetylcholine. |  |  |
| Aliskiren is an antagonist to voltage-dependent calcium channels. |  |  |
| Nebivolol acts as a vasodilator, because of its influence on the production of endogenous nitric oxide. |  |  |
| Aztreonam is an antibiotic which belongs to the group of monobactams. |  |  |
| Glucocorticoids work by intracellular receptors. |  |  |
| Benzylpenicillin is the only natural penicillin which is administered orally. |  |  |
| Sartans significantly more frequently lead to hyperkalemia in comparison to angiotensin-converting-enzyme inhibitors. |  |  |
| Bupropion is a particular antidepresive drug, because it has no influence on the serotonin reuptake and it might be administered with SSRIs. |  |  |
| Alfa-adrenolytics are drugs used in first line therapy of hypertension. |  |  |
| The maximum daily dose of ramipril used in hypertension is 100 mg. |  |  |
| Oxidation of barbituran’s aliphatic chains belongs to the I phase of biotransformation. |  |  |
| Using NSAIDs in a patient suffering from diabetes type 2, who is treated with sulphonylureas, increases the risk of hypoglycemia. |  |  |
| Diclofenac increases the plasma concentration of simultaneously used lithium, because it displaces lithium from plasma protein binding sites. |  |  |
| Clindamycin, similarly to macrolides, works as an antibacterial agent because of binding to the 50S subunit of bacterial RNA. |  |  |
| Aluminium salts increase bioavailability of oral norfloxacin, because aluminium cations bind to the molecules of this chemotherapeutic. |  |  |
| Ticagrelor is an antagonist to thromboxane A2 receptors. |  |  |
| Aminoglycosides are mainly excreted with bile, so patients with liver failure need a reduction of aminoglycosides dose. |  |  |
| Oral drotaverine more often causes dry mouth and constipation than oral butylscopolamine bromide. |  |  |
| Rocuronium bromide is a non-depolarizing muscle relaxant. |  |  |
| Omalizumab is an humanized monoclonal antibody used in treatment of allergy-originated asthma. |  |  |
| Rifampicin belongs to strong inhibitors of CYP3A4 isoform of cytochrome P450. |  |  |

**Survey 2**

Survey 2 was sent. Thank you! We wish you good luck in tomorrow’s pharmacology exam!

We kindly remind you that the food you eat should still be recorded in the form “My meal” - the record of the food you eat should be sent by the time when the exam starts.
