# Supplementary material for: Association Between Consumption of Fermented Food and Food-Derived Prebiotics With Cognitive Performance, Depressive, and Anxiety Symptoms in Psychiatrically Healthy Medical Students Under Psychological Stress: A Prospective Cohort Study
Source: Front Nutr. 2022 Mar 3;9:850249. doi: 10.3389/fnut.2022.850249 (PMC8929173; doi:10.3389/fnut.2022.850249)
Supplement: Supplementary file 6 [file Data_Sheet_6.DOCX]

***Supplementary Material 6***

***Association between consumption of fermented food and food-derived prebiotics with cognitive performance under stress, depressive and anxiety symptoms – sensitivity analysis***

A) The results presented herein are based on the subgroup of participants with high adherence to food recording.

Table. Association between consumption of fermented food and food-derived prebiotics with cognitive performance under stress, depressive and anxiety symptoms – a subgroup missing no single day of the food recording (n=313).

| Product consumed | β (95% CI), p-value | |
| --- | --- | --- |
|  | Raw analysis | Adjusted analysis |
| Cognitive performance under stress | | |
| Fermented food | 0.04 (-0.06 to 0.13), *p*=0.46 | 0.03 (-0.06 to 0.13), *p*=0.49 |
| Food-derived prebiotics | 0.00 (-0.09 to 0.10), *p*=0.93 | -0.01 (-0.11 to 0.09), *p*=0.84 |
| Pre-exam depressive symptoms | | |
| Fermented food | 0.14 (0.03 to 0.25), *p*=0.015 | 0.14 (0.04 to 0.25), *p*=0.0075 |
| Food-derived prebiotics | -0.05 (-0.16 to 0.06), *p*=0.37 | 0.05 (-0.06 to 0.16), *p*=0.38 |
| Pre-exam anxiety symptoms | | |
| Fermented food | 0.15 (0.04 to 0.26), *p*=0.0089 | 0.16 (0.06 to 0.26), *p*=0.0018 |
| Food-derived prebiotics | -0.08 (-0.19 to 0.03), *p*=0.14 | 0.02 (-0.09 to 0.12), *p*=0.74 |

Table. Association between consumption of fermented food and food-derived prebiotics with cognitive performance under stress, depressive and anxiety symptoms – a subgroup declaring to miss no more than 10% of consumed foodstuffs in their food records (n=289)

| Product consumed | β (95% CI), p-value | |
| --- | --- | --- |
|  | Raw analysis | Adjusted analysis |
| Cognitive performance under stress | | |
| Fermented food | 0.03 (-0.08 to 0.13), *p*=0.62 | 0.04 (-0.06 to 0.15), *p*=0.43 |
| Food-derived prebiotics | 0.03 (-0.07 to 0.14), *p*=0.51 | 0.04 (-0.07 to 0.16), *p*=0.43 |
| Pre-exam depressive symptoms | | |
| Fermented food | 0.13 (0.01 to 0.24), *p*=0.030 | 0.16 (0.05 to 0.27), *p*=0.0036 |
| Food-derived prebiotics | -0.04 (-0.16 to 0.08), *p*=0.50 | 0.05 (-0.06 to 0.16), *p*=0.39 |
| Pre-exam anxiety symptoms | | |
| Fermented food | 0.08 (-0.03 to 0.20), *p*=0.16 | 0.13 (0.02 to 0.23), *p*=0.015 |
| Food-derived prebiotics | -0.12 (-0.23 to -0.00), *p*=0.045 | -0.03 (-0.14 to 0.08), *p*=0.57 |

B) The results presented herein are based on the 7-day consumption of fermented food calculated with exclusion of probiotic dietary supplements and medicinal products

Table. Association between 7-day consumption of fermented food (not including probiotic dietary supplements and medicinal products) with cognitive performance under stress, depressive and anxiety symptoms – a total sample (n=372).

| Product consumed | β (95% CI), p-value | |
| --- | --- | --- |
|  | Raw analysis | Adjusted analysis |
| Cognitive performance under stress | | |
| Fermented food | 0.01 (-0.08 to 0.10), *p*=0.84 | 0.01 (-0.08 to 0.10), *p*=0.76 |
| Pre-exam depressive symptoms | | |
| Fermented food | 0.09 (-0.01 to 0.20), *p*=0.072 | 0.12 (0.02 to 0.21), *p*=0.018 |
| Pre-exam anxiety symptoms | | |
| Fermented food | 0.10 (-0.01 to 0.20), *p*=0.063 | 0.14 (0.05 to 0.23), *p*=0.0035 |

C) The results presented herein are based on the 3-day consumption of fermented food and food-derived prebiotics.

Table. Association between 3-day consumption of fermented food and food-derived prebiotics with cognitive performance under stress, depressive and anxiety symptoms – a total sample (n=372).

| Product consumed | β (95% CI), p-value | |
| --- | --- | --- |
|  | Raw analysis | Adjusted analysis |
| Cognitive performance under stress | | |
| Fermented food | -0.00 (-0.09 to 0.08), *p*=0.96 | 0.00 (-0.09 to 0.09), *p*=0.97 |
| Food-derived prebiotics | -0.07 (-0.16 to 0.01), *p*=0.10 | -0.08 (-0.17 to 0.01), *p*=0.089 |
| Pre-exam depressive symptoms | | |
| Fermented food | 0.06 (-0.04 to 0.16), *p*=0.25 | 0.09 (-0.01 to 0.18), *p*=0.080 |
| Food-derived prebiotics | -0.06 (-0.16 to 0.04), *p*=0.23 | -0.00 (-0.10 to 0.10), *p*=0.95 |
| Pre-exam anxiety symptoms | | |
| Fermented food | 0.09 (-0.01 to 0.19), *p*=0.094 | 0.12 (0.03 to 0.22), *p*=0.0075 |
| Food-derived prebiotics | -0.09 (-0.19 to 0.01), *p*=0.087 | -0.01 (-0.10 to 0.09), *p*=0.89 |

Correlation between the 7-day fermented food consumption and that of 3-day:

r=0.86, *p*<0.0001

Correlation between the 7-day food-derived prebiotic consumption and that of 3-day:

r=0.81, *p*<0.0001
