# Supplementary material for: Association Between Consumption of Fermented Food and Food-Derived Prebiotics With Cognitive Performance, Depressive, and Anxiety Symptoms in Psychiatrically Healthy Medical Students Under Psychological Stress: A Prospective Cohort Study
Source: Front Nutr. 2022 Mar 3;9:850249. doi: 10.3389/fnut.2022.850249 (PMC8929173; doi:10.3389/fnut.2022.850249)
Supplement: Supplementary file 7 [file Data_Sheet_7.DOCX]

***Supplementary Material 7***

***Stability of the associations between consumption of fermented food and food-derived prebiotics with cognitive performance under stress, depressive and anxiety symptoms across multiple participants’ characteristics – interaction analysis***

The results of interactions between the selected covariates (sex, BMI, physical activity, quality of diet, smoking, personality traits, depressive and anxiety symptoms, or subject knowledge in pharmacology) and consumption of fermented food and food-derived prebiotics in predicting cognitive performance under stress, depressive and anxiety symptoms. Significant interaction may be interpreted as different effect size for the association of interest in subgroups of different level of a covariate.

COGNITIVE PERFORMANCE

Table. Association between **cognitive performance under stress** and consumption of fermented food and food derived prebiotics – the effect of interaction with a covariate

| Covariate | β (95% CI), *p*-value | |
| --- | --- | --- |
|  | Raw analysis | Adjusted analysis |
| **Fermented food** | | |
| Sex (female=0, male=1) | 0.01 (-0.16 to 0.18), *p=*0.93 | -0.00 (-0.17 to 0.17), *p=*0.98 |
| BMI | -0.32 (-1.01 to 0.38), *p=*0.37 | -0.28 (-0.99 to 0.43), *p=*0.44 |
| Smoking | -0.01 (-0.15 to 0.14), *p=*0.92 | 0.01 (-0.15 to 0.16), *p=*0.94 |
| General diet quality | -0.03 (-0.31 to 0.24), *p=*0.81 | -0.06 (-0.34 to 0.22), *p=*0.68 |
| Pre-exam physical activity | 0.11 (-0.11 to 0.34), *p=*0.31 | 0.17 (-0.06 to 0.40), *p=*0.14 |
| Neuroticism | -0.02 (-0.32 to 0.29), *p=*0.92 | 0.01 (-0.30 to 0.33), *p=*0.93 |
| Extraversion | 0.05 (-0.23 to 0.34), *p=*0.72 | 0.03 (-0.26 to 0.32), *p=*0.83 |
| Openness | 0.30 (0.07 to 0.67), *p=*0.11 | 0.28 (-0.10 to 0.67), *p=*0.15 |
| Agreeableness | -0.12 (-0.53 to 0.28), *p=*0.55 | -0.18 (-0.61 to 0.24), *p=*0.39 |
| Conscientiousness | -0.15 (-0.54 to 0.24), *p=*0.45 | -0.11 (-0.51 to 0.29), *p=*0.60 |
| Pre-exam depressive symptoms | -0.05 (-0.24 to 0.15), *p=*0.65 | -0.07 (-0.27 to 0.14), *p=*0.54 |
| Pre-exam anxiety symptoms | 0.02 (-0.17 to 0.21), *p=*0.85 | -0.00 (-0.20 to 0.19), *p=*0.98 |
| **Food-derived prebiotics** | | |
| Sex (female=0, male=1) | -0.15 (-0.31 to 0.02), *p=*0.091 | -0.16 (-0.33 to 0.02), *p=*0.074 |
| BMI | -0.20 (-0.84 to 0.43), *p=*0.53 | -0.20 (-0.85 to 0.45), *p=*0.54 |
| Smoking | -0.10 (-0.25 to 0.04), *p=*0.16 | -0.12 (-0.27 to 0.02), *p=*0.098 |
| General diet quality | 0.04 (-0.22 to 0.30), *p=*0.76 | 0.01 (-0.26 to 0.27), *p=*0.96 |
| Pre-exam physical activity | -0.07 (-0.31 to 0.16), *p=*0.54 | -0.13 (-0.37 to 0.12), *p=*0.30 |
| Neuroticism | -0.14 (-0.45 to 0.17), *p=*0.36 | -0.10 (-0.42 to 0.21), *p=*0.52 |
| Extraversion | -0.05 (-0.38 to 0.28), *p=*0.76 | -0.01 (-0.35 to 0.32), *p=*0.93 |
| Openness | -0.07 (-0.47 to 0.33), *p=*0.73 | -0.04 (-0.45 to 0.37), *p=*0.85 |
| Agreeableness | -0.19 (-0.62 to 0.24), *p=*0.39 | -0.17 (-0.60 to 0.27), *p=*0.46 |
| Conscientiousness | -0.27 (-0.72 to 0.18), *p=*0.23 | -0.30 (-0.76 to 0.15), *p=*0.19 |
| Pre-exam depressive symptoms | 0.10 (-0.10 to 0.30), *p=*0.33 | 0.13 (-0.07 to 0.33), *p=*0.21 |
| Pre-exam anxiety symptoms | 0.05 (-0.14 to 0.24), *p=*0.57 | 0.06 (-0.14 to 0.25), *p=*0.57 |

DEPRESSIVE SYMPTOMS

Table. Association between **depressive symptoms** and consumption of fermented food and food derived prebiotics – the effect of interaction with a covariate

| Covariate | β (95% CI), *p*-value | |
| --- | --- | --- |
|  | Raw analysis | Adjusted analysis |
| **Fermented food** | | |
| Sex (female=0, male=1) | 0.16 (-0.04 to 0.35), *p=*0.11 | 0.15 (-0.03 to 0.33), *p=*0.10 |
| BMI | 0.05 (-0.75 to 0.84), *p=*0.91 | 0.23 (-0.52 to 0.97), *p=*0.55 |
| Smoking | -0.22 (-0.39 to -0.05), *p=*0.012 | -0.21 (-0.37 to -0.05), *p=*0.011 |
| General diet quality | 0.11 (-0.21 to 0.43), *p=*0.50 | 0.11 (-0.19 to 0.42), *p=*0.48 |
| Pre-exam physical activity | -0.21 (-0.46 to 0.04), *p=*0.10 | -0.30 (-0.55 to -0.05), *p=*0.018 |
| Neuroticism | 0.09 (-0.25 to 0.43), *p=*0.60 | 0.13 (-0.21 to 0.47), *p=*0.44 |
| Extraversion | 0.25 (-0.07 to 0.58), *p=*0.13 | 0.23 (-0.08 to 0.54), *p=*0.15 |
| Openness | -0.42 (-0.85 to 0.01), *p=*0.057 | -0.47 (-0.88 to -0.05), *p=*0.027 |
| Agreeableness | 0.31 (-0.15 to 0.78), *p=*0.19 | 0.40 (-0.05 to 0.85), *p=*0.080 |
| Conscientiousness | 0.18 (-0.27 to 0.63), *p=*0.43 | 0.18 (-0.25 to 0.62), *p=*0.40 |
| Pre-exam anxiety symptoms | -0.02 (-0.16 to 0.12), *p=*0.77 | -0.01 (-0.15 to 0.14), *p=*0.93 |
| Subject knowledge (exam points adjusted to exam round) | -0.45 (-1.17 to 0.26), *p=*0.21 | -0.38 (-1.05 to 0.29), *p=*0.27 |
| **Food-derived prebiotics** | | |
| Sex (female=0, male=1) | 0.15 (-0.05 to 0.35), *p=*0.13 | 0.11 (-0.07 to 0.30), *p=*0.23 |
| BMI | 0.06 (-0.68 to 0.81), *p=*0.87 | -0.23 (-0.94 to 0.47), *p=*0.51 |
| Smoking | -0.04 (-0.21 to 0.13), *p=*0.63 | -0.02 (-0.18 to 0.14), *p=*0.85 |
| General diet quality | -0.00 (-0.31 to 0.30), *p=*0.98 | 0.02 (-0.27 to 0.30), *p=*0.92 |
| Pre-exam physical activity | -0.11 (-0.38 to 0.17), *p=*0.44 | -0.17 (-0.43 to 0.10), *p=*0.21 |
| Neuroticism | -0.07 (-0.42 to 0.27), *p=*0.68 | -0.03 (-0.37 to 0.32), *p=*0.88 |
| Extraversion | 0.20 (-0.18 to 0.58), *p=*0.31 | 0.03 (-0.33 to 0.40), *p=*0.86 |
| Openness | 0.11 (-0.36 to 0.58), *p=*0.65 | -0.14 (-0.60 to 0.30), *p=*0.52 |
| Agreeableness | 0.48 (-0.02 to 0.98), *p=*0.057 | 0.28 (-0.20 to 0.75), *p=*0.25 |
| Conscientiousness | -0.18 (-0.70 to 0.34), *p=*0.49 | -0.25 (-0.74 to 0.25), *p=*0.32 |
| Pre-exam anxiety symptoms | -0.07 (-0.22 to 0.07), *p=*0.33 | -0.07 (-0.21 to 0.08), *p=*0.37 |
| Subject knowledge (exam points adjusted to exam round) | 0.18 (-0.54 to 0.90), *p=*0.62 | 0.28 (-0.40 to 0.96), *p=*0.41 |

Table. Association between consumption of **fermented food** and **depressive symptoms** in subgroups revealed by the interaction analyses

| Subgroup | β (95% CI), *p*-value (adjusted analyses) |
| --- | --- |
| Smoking | |
| Non-smokers (n=347) | 0.14 (0.04 to 0.24), *p*=0.0071 |
| Smokers (n=25) | -0.44 (-1.09 to 0.21), *p*=0.16 |
| Physical activity | |
| No physical activity at all (n=129) | 0.20 (0.03 to 0.37), *p*=0.019 |
| Physical activity higher than median (n=152) | 0.07 (-0.10 to 0.25), *p*=0.39 |
| Personality | |
| Openness lower than median (n=175) | 0.23 (0.08 to 0.37), *p*=0.0026 |
| Openness higher than median (n=161) | 0.01 (-0.15 to 0.16), *p*=0.91 |

ANXIETY SYMPTOMS

Table. Association between **anxiety symptoms** and consumption of fermented food and food derived prebiotics – the effect of interaction with a covariate

| Covariate | β (95% CI), *p*-value | |
| --- | --- | --- |
|  | Raw analysis | Adjusted analysis |
| **Fermented food** | | |
| Sex (female=0, male=1) | 0.13 (-0.06 to 0.32), *p=*0.19 | 0.10 (-0.07 to 0.27), *p=*0.25 |
| BMI | -0.00 (-0.79 to 0.79), p>0.99 | 0.26 (-0.44 to 0.96), *p=*0.47 |
| Smoking | -0.22 (-0.39 to -0.05), *p=*0.010 | -0.20 (-0.35 to -0.05), *p=*0.011 |
| General diet quality | 0.12 (-0.20 to 0.43), *p=*0.47 | 0.14 (-0.14 to 0.43), *p=*0.33 |
| Pre-exam physical activity | -0.17 (-0.42 to 0.07), *p=*0.17 | -0.25 (-0.48 to -0.01), *p=*0.039 |
| Neuroticism | -0.10 (-0.42 to 0.23), *p=*0.56 | -0.03 (-0.35 to 0.29), *p=*0.84 |
| Extraversion | 0.15 (-0.18 to 0.48), *p=*0.37 | 0.11 (-0.19 to 0.40), *p=*0.47 |
| Openness | -0.25 (-0.69 to 0.18), *p=*0.25 | -0.28 (-0.67 to 0.11), *p=*0.16 |
| Agreeableness | 0.27 (-0.19 to 0.74), *p=*0.25 | 0.35 (-0.07 to 0.78), *p=*0.10 |
| Conscientiousness | 0.38 (-0.08 to 0.83), *p=*0.10 | 0.38 (-0.03 to 0.79), *p=*0.066 |
| Pre-exam depressive symptoms | 0.04 (-0.10 to 0.19), *p=*0.56 | 0.04 (-0.10 to 0.19), *p=*0.57 |
| Subject knowledge (exam points adjusted to exam round) | -0.19 (-0.91 to 0.53), *p=*0.60 | -0.22 (-0.86 to 0.43), *p=*0.51 |
| **Food-derived prebiotics** | | |
| Sex (female=0, male=1) | 0.12 (-0.08 to 0.31), *p=*0.24 | 0.08 (-0.10 to 0.26), *p=*0.38 |
| BMI | 0.26 (-0.47 to 1.00), *p=*0.48 | -0.05 (-0.73 to 0.62), *p=*0.87 |
| Smoking | -0.08 (-0.25 to 0.10), *p=*0.39 | -0.06 (-0.21 to 0.10), *p=*0.47 |
| General diet quality | -0.08 (-0.38 to 0.22), *p=*0.58 | -0.10 (-0.37 to 0.17), *p=*0.45 |
| Pre-exam physical activity | 0.04 (-0.24 to 0.31), *p=*0.79 | -0.03 (-0.29 to 0.22), *p=*0.79 |
| Neuroticism | -0.16 (-0.50 to 0.17), *p=*0.33 | -0.11 (-0.44 to 0.22), *p=*0.51 |
| Extraversion | 0.24 (-0.15 to 0.62), *p=*0.23 | 0.08 (-0.27 to 0.42), *p=*0.65 |
| Openness | 0.08 (-0.39 to 0.55), *p=*0.74 | -0.18 (-0.60 to 0.24), *p=*0.41 |
| Agreeableness | 0.16 (-0.34 to 0.65), *p=*0.54 | -0.09 (-0.54 to 0.36), *p=*0.70 |
| Conscientiousness | 0.08 (-0.45 to 0.60), *p=*0.78 | -0.01 (-0.48 to 0.46), *p=*0.96 |
| Pre-exam depressive symptoms | -0.07 (-0.22 to 0.08), *p=*0.37 | -0.05 (-0.19 to 0.10), *p=*0.54 |
| Subject knowledge (exam points adjusted to exam round) | -0.01 (-0.74 to 0.71), *p=*0.97 | 0.02 (-0.63 to 0.68), *p=*0.94 |

Table. Association between consumption of **fermented food** and **anxiety symptoms** in subgroups revealed by the interaction analyses

| Subgroup | β (95% CI), *p*-value (adjusted analyses) |
| --- | --- |
| Smoking | |
| Non-smokers (n=347) | 0.16 (0.06 to 0.25), *p*=0.0011 |
| Smokers (n=25) | -0.30 (-1.04 to 0.43), *p*=0.37 |
| Physical activity | |
| No physical activity at all (n=129) | 0.26 (0.10 to 0.41), *p*=0.0018 |
| Physical activity higher than median (n=152) | 0.09 (-0.07 to 0.26), *p*=0.27 |
