# Supplementary material for: Association Between Consumption of Fermented Food and Food-Derived Prebiotics With Cognitive Performance, Depressive, and Anxiety Symptoms in Psychiatrically Healthy Medical Students Under Psychological Stress: A Prospective Cohort Study
Source: Front Nutr. 2022 Mar 3;9:850249. doi: 10.3389/fnut.2022.850249 (PMC8929173; doi:10.3389/fnut.2022.850249)
Supplement: Supplementary file 8 [file Data_Sheet_8.DOCX]

***Supplementary Material 8***

***Associations between consumption of each fermented and prebiotic-containing foodstuff with cognitive performance under stress, depressive and anxiety symptoms***

COGNITIVE PERFORMANCE

Table. Association between **cognitive performance under stress** and consumption of each fermented and prebiotic-containing food product

| Food product | β (95% CI), *p*-value | |
| --- | --- | --- |
|  | Raw analysis | Adjusted analysis |
| **Fermented food products** | | |
| Cheese | -0.02 (-0.10 to 0.07), *p=*0.70 | -0.01 (-0.10 to 0.08), *p=*0.88 |
| Yogurt, kefir, soured milk | 0.02 (-0.07 to 0.11), *p=*0.68 | 0.02 (-0.07 to 0.11), *p=*0.65 |
| Kvass and unpasteurised beer | 0.01 (-0.08 to 0.10), *p=*0.82 | 0.01 (-0.08 to 0.10), *p=*0.84 |
| Pickled cucumber and pickling juice | 0.01 (-0.08 to 0.10), *p=*0.85 | 0.00 (-0.09 to 0.09), *p=*0.99 |
| Sauerkraut and pickling juice | 0.01 (-0.08 to 0.10), *p=*0.79 | 0.03 (-0.06 to 0.12), *p=*0.47 |
| Other fermented vegetables and their pickling juice | -0.05 (-0.14 to 0.03), *p=*0.22 | -0.04 (-0.13 to 0.05), *p=*0.39 |
| Probiotic dietary supplements or medicinal products | 0.04 (-0.05 to 0.12), *p=*0.42 | 0.02 (-0.07 to 0.12), *p=*0.61 |
| **Food-derived prebiotics** | | |
| Wholemeal bread, graham | 0.00 (-0.09 to 0.09), *p=*0.99 | -0.01 (-0.11 to 0.08), *p=*0.75 |
| Cereal, groats, whole grain noodle | 0.04 (-0.04 to 0.13), *p=*0.32 | 0.04 (-0.05 to 0.13), *p=*0.37 |
| Muesli | -0.03 (-0.12 to 0.06), *p=*0.55 | -0.02 (-0.11 to 0.07), *p=*0.61 |
| Wholemeal flour | -0.04 (-0.12 to 0.05), *p=*0.41 | -0.03 (-0.12 to 0.06), *p=*0.47 |
| Onion, leek, garlic | 0.00 (-0.08 to 0.09), *p=*0.94 | 0.02 (-0.07 to 0.11), *p=*0.71 |
| Bananas | -0.04 (-0.13 to 0.05), *p=*0.36 | -0.05 (-0.14 to 0.04), *p=*0.27 |
| Asparagus, chicory root, dandelion leaves, globe artichoke and Jerusalem artichoke | -0.03 (-0.12 to 0.05), *p=*0.45 | -0.03 (-0.12 to 0.06), *p=*0.55 |
| Prebiotic dietary supplements or medicinal products | 0.02 (-0.07 to 0.11), *p=*0.65 | 0.01 (-0.08 to 0.10), *p=*0.80 |

DEPRESSIVE SYMPTOMS

Table. Association between **depressive symptoms** and consumption of each fermented and prebiotic-containing food product

| Food product | β (95% CI), *p*-value | |
| --- | --- | --- |
|  | Raw analysis | Adjusted analysis |
| **Fermented food products** | | |
| Cheese | 0.11 (0.01 to 0.21), *p=*0.036 | 0.08 (-0.02 to 0.18), *p=*0.10 |
| Yogurt, kefir, soured milk | 0.03 (-0.07 to 0.13), *p=*0.58 | 0.04 (-0.06 to 0.14), *p=*0.42 |
| Kvass and unpasteurised beer | 0.09 (-0.01 to 0.19), *p=*0.084 | 0.11 (0.02 to 0.21), *p=*0.023 |
| Pickled cucumber and pickling juice | -0.02 (-0.12 to 0.09), *p=*0.75 | 0.03 (-0.07 to 0.12), *p=*0.59 |
| Sauerkraut and pickling juice | 0.03 (-0.07 to 0.14), *p=*0.50 | 0.08 (-0.01 to 0.18), *p=*0.090 |
| Other fermented vegetables and their pickling juice | 0.03 (-0.08 to 0.13), *p=*0.61 | 0.00 (-0.10 to 0.10), *p=*0.98 |
| Probiotic dietary supplements or medicinal products | -0.07 (-0.17 to 0.04), *p=*0.20 | -0.03 (-0.13 to 0.07), *p=*0.56 |
| **Food-derived prebiotics** | | |
| Wholemeal bread, graham | -0.09 (-0.19 to 0.01), *p=*0.090 | -0.02 (-0.12 to 0.08), *p=*0.70 |
| Cereal, groats, whole grain noodle | -0.10 (-0.20 to 0.00), *p=*0.057 | -0.03 (-0.12 to 0.07), *p=*0.61 |
| Muesli | 0.01 (-0.10 to 0.11), *p=*0.90 | 0.05 (-0.04 to 0.15), *p=*0.29 |
| Wholemeal flour | 0.06 (-0.04 to 0.16), *p=*0.23 | 0.07 (-0.03 to 0.17), *p=*0.16 |
| Onion, leek, garlic | 0.02 (-0.09 to 0.12), *p=*0.74 | 0.04 (-0.06 to 0.13), *p=*0.47 |
| Bananas | -0.11 (-0.21 to -0.01), *p=*0.033 | -0.06 (-0.16 to 0.04), *p=*0.21 |
| Asparagus, chicory root, dandelion leaves, globe artichoke and Jerusalem artichoke | 0.03 (-0.07 to 0.14), *p=*0.50 | 0.03 (-0.07 to 0.13), *p=*0.53 |
| Prebiotic dietary supplements or medicinal products | 0.06 (-0.04 to 0.17), *p=*0.22 | 0.07 (-0.03 to 0.17), *p=*0.15 |

Table. Association of consumption of fermented food (estimated with exclusion of “kvass and unpasteurised beer”) with depressive symptoms

| Consumption of fermented food (estimated with exclusion of “kvass and unpasteurised beer”) | β (95% CI), *p*-value (adjusted analyses) |
| --- | --- |
|  | 0.08 (0.02 to 0.18), *p=*0.11 |

ANXIETY SYMPTOMS

Table. Association between **anxiety symptoms** and consumption of each fermented and prebiotic-containing food product

| Food product | β (95% CI), *p*-value | |
| --- | --- | --- |
|  | Raw analysis | Adjusted analysis |
| **Fermented food products** | | |
| Cheese | 0.08 (-0.02 to 0.19), *p=*0.10 | 0.06 (-0.03 to 0.15), *p=*0.20 |
| Yogurt, kefir, soured milk | 0.05 (-0.05 to 0.16), *p=*0.30 | 0.08 (-0.01 to 0.17), *p=*0.084 |
| Kvass and unpasteurised beer | 0.06 (-0.04 to 0.17), *p=*0.21 | 0.10 (0.01 to 0.19), *p=*0.030 |
| Pickled cucumber and pickling juice | -0.02 (-0.13 to 0.08), *p=*0.63 | 0.01 (-0.08 to 0.10), *p=*0.84 |
| Sauerkraut and pickling juice | 0.01 (-0.10 to 0.11), *p=*0.90 | 0.08 (-0.01 to 0.17), *p=*0.097 |
| Other fermented vegetables and their pickling juice | 0.04 (-0.07 to 0.14), *p=*0.48 | 0.01 (-0.08 to 0.11), *p=*0.77 |
| Probiotic dietary supplements or medicinal products | -0.07 (-0.17 to 0.04), *p=*0.20 | -0.03 (-0.12 to 0.07), *p=*0.55 |
| **Food-derived prebiotics** | | |
| Wholemeal bread, graham | -0.11 (-0.22 to -0.01), *p=*0.028 | -0.04 (-0.14 to 0.05), *p=*0.38 |
| Cereal, groats, whole grain noodle | -0.10 (-0.20 to -0.00), *p=*0.047 | -0.02 (-0.12 to 0.07), *p=*0.63 |
| Muesli | -0.01 (-0.12 to 0.09), *p=*0.79 | 0.05 (-0.04 to 0.15), *p=*0.24 |
| Wholemeal flour | 0.01 (-0.09 to 0.11), *p=*0.90 | 0.03 (-0.06 to 0.13), *p=*0.47 |
| Onion, leek, garlic | -0.05 (-0.15 to 0.05), *p=*0.34 | -0.03 (-0.12 to 0.06), *p=*0.56 |
| Bananas | -0.15 (-0.25 to -0.05), *p=*0.0034 | -0.07 (-0.17 to -0.02), *p=*0.12 |
| Asparagus, chicory root, dandelion leaves, globe artichoke and Jerusalem artichoke | 0.10 (0.00 to 0.21), *p=*0.040 | 0.10 (0.01 to 0.20), *p=*0.028 |
| Prebiotic dietary supplements or medicinal products | 0.04 (-0.06 to 0.15), *p=*0.39 | 0.05 (-0.04 to 0.14), *p=*0.28 |

Table. Association of consumption of fermented food (estimated with exclusion of “kvass and unpasteurised beer”) with anxiety symptoms

| Consumption of fermented food (estimated with exclusion of “kvass and unpasteurised beer”) | β (95% CI), *p*-value (adjusted analyses) |
| --- | --- |
|  | 0.10 (0.01 to 0.19), *p=*0.029 |
